# Isolation and characterization of a multifunctional flavonoid glycosyltransferase from *Ornithogalum caudatum* with glycosidase activity

**DOI:** 10.1038/s41598-018-24277-3

**Published:** 2018-04-12

**Authors:** Shuai Yuan, Sen Yin, Ming Liu, Jian-Qiang Kong

**Affiliations:** Institute of Materia Medica, Chinese Academy of Medical Sciences & Peking Union Medical College (State Key Laboratory of Bioactive Substance and Function of Natural Medicines & Ministry of Health Key Laboratory of Biosynthesis of Natural Products), Beijing, 100050 China

## Abstract

Glycosyltransferases (GTs) are bidirectional biocatalysts catalyzing the glycosylation of diverse molecules. However, the extensive applications of GTs in glycosides formation are limited due to their requirements of expensive nucleotide diphosphate (NDP)-sugars or NDP as the substrates. Here, in an effort to characterize flexible GTs for glycodiversification of natural products, we isolated a cDNA, designated as *OcUGT1* from *Ornithogalum caudatum*, which encoded a flavonoid GT that was able to catalyze the trans-glycosylation reactions, allowing the formation of glycosides without the additions of NDP-sugars or NDP. In addition, OcUGT1 was observed to exhibit additional five types of functions, including classical sugar transfer reaction and three reversible reactions namely NDP-sugar synthesis, sugars exchange and aglycons exchange reactions, as well as enzymatic hydrolysis reaction, suggesting OcUGT1 displays both glycosyltransferase and glycosidase activities. Expression profiles revealed that the expression of OcUGT1 was development-dependent and affected by environmental factors. The unusual multifunctionality of OcUGT1 broadens the applicability of OcUGT1, thereby generating diverse carbohydrate-containing structures.

## Introduction

**Figure 1:**
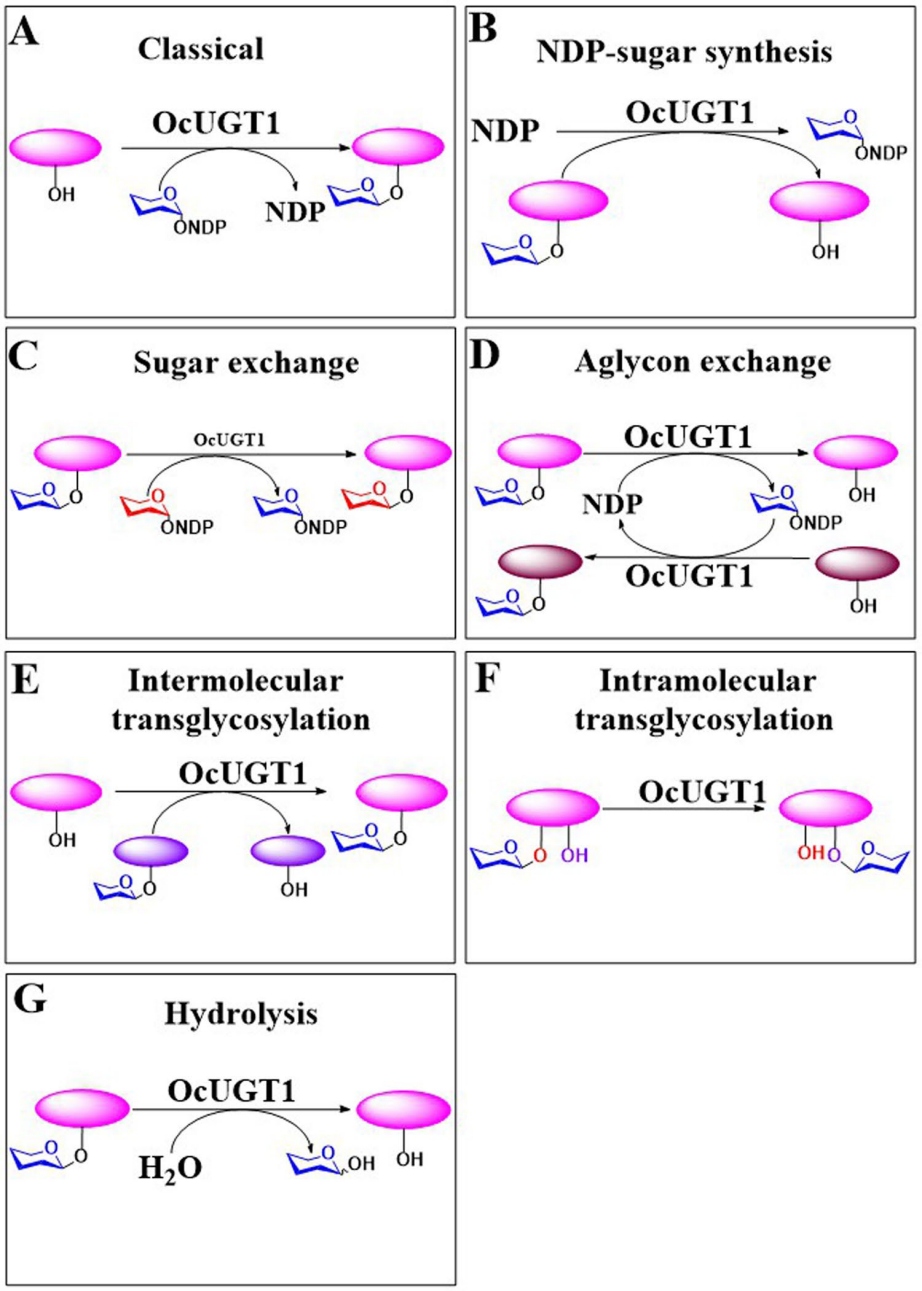
Schematic of OcUGT1-catalyzed reactions. (**A**) The classical sugar transfer, (**B**) NDP-sugar synthesis, (**C**) sugar exchange, (**D**) aglycon exchange, (**E**) Intermolecular trans-glycosylation, (**F**) intramolecular trans-glycosylation, (**G**) hydrolysis.

Glycosylation is one of the most important modification reactions, in which sugar moieties are transferred to small molecular acceptors thereby leading to the formation of diverse glycosides with therapeutical potentials^1^. The enzyme-based glycosylations are mainly performed by two types of carbohydrate-active enzymes (CAZymes), glycosyltransferases (GTs, EC 2.4.x.y) and glycosidases (glycoside hydrolases; EC 3.2.x.y)^2^. Glycosidases are naturally hydrolytic enzymes that normally cleave glycosidic bonds^3^. However, glycosidase-assisted glycosylations are able to be achieved by trans-glycosylation reactions under certain controlled conditions^4^. The majority of glycosylation reactions in nature are performed in GT-catalyzed fashion, in which the carbohydrate moieties from activated NDP-sugars are transferred to acceptor molecules^5^. Also, GT-catalyzed glycosylations were accomplished by running the GT reactions in reverse^6^. New glycosides can be generated by reversible sugar or aglycon exchange reactions catalyzed by GTs with the addition of excess of NDP^6,7^. It is therefore obvious that GTs are generally perceived as bidirectional biocatalysts used in glycosylation of small molecules^1,8,9^. Yet, despite the validated potential of GTs in glycosides production, the extensive applications of GTs in industry remained limited due to the requirement of expensive NDP-sugars or NDP in GT-catalyzed forward or reversible reactions. Here, we report that a flavonoid GT, designated as OcUGT1, catalyzes the trans-glycosylation reactions, allowing the formation of glycosides without the additions of NDP-sugars or NDP. Specifically, in a screen of novel biocatalysts for flavonoid glycosylations from medicinal plant *Ornithogalum caudatum*, we found a multifunctional flavonoid GT OcUGT1 exhibiting at least six types of functions, including classical sugar transfer reaction and three reversible reactions namely NDP-sugar synthesis, sugars exchange and aglycons exchange reactions, as well as glycosidase specific hydrolysis and trans-glycosylation reactions. These evidences indicated that OcUGT1 shows both glycosyltransferase and glycosidase activities (Fig. 1). The unusual multifunctionality of OcUGT1 broadens its applicability, thereby enhancing the glycoconjugates diversity.

## Results and Discussion

### Retrieve of unigenes encoding GTs from *O*. *caudatum* RNA-Seq dataset

Flavonoid glycosides have been shown to exert diverse pharmacological activities^10–12^. Many of these flavonoid glycosides were hence developed to clinical drugs, including rutin^13–15^, puerarin^16^ and scutellarin^17^. The growing interest for the therapeutic potential of flavonoid glycosides has spurred increasing efforts in enzymatic glycodiversification of flavonoids. Glycodiversification had long been known to be monitored mainly by GTs^18^. The discovery of GTs with expanded promiscuity is therefore a necessary prerequisite for diversifying natural products glycosylation in practice. *O*. *caudatum*, also named *O*. *longibracteatum*, is known in Chinese folk medicine to have anticancer, antimicrobial and anti-inflammatory activities^19^. Prior studies revealed that varied flavonoid glycosides, including flavonoid glucosides, galactosides, arabinosides and rutinosides were present in *O*. *caudatum*^19–21^, suggesting the existence of corresponding flavonoid GTs. Thus, *O*. *caudatum* was chosen as the plant template for flavonoid GTs isolation in the present work.

A transcriptome dataset of *O*. *caudatum* was reported previously^22–24^. The assembled sequences (104,180 unigenes) were thus aligned by Blast X to protein databases like nr, Swiss-Prot, KEGG and COG (e-value < 0.00001) with the aim of retrieving unigenes sharing the high sequence similarity with flavonoid GTs. Unigene 26797 was thus retrieved from the transcriptome database for further investigation due to its high identity with UDP-glucose-dependent flavonoid glucosyltransferase. ORF Finder analysis revealed that unigene 26797 is 1926 bp long and contains a full-length open reading frame (ORF) of 1440 bp encoding a putative protein of 379 amino acids. Moreover, unigene 26797 consisted of a partial 5′-UTR (untranslated region) of 126 bp and 3′-UTR of 360 bp. The ORF sequence of unigene 26797 was tentatively designated OcUGT1.

### cDNA isolation and sequence analysis of OcUGT1

To validate the authenticity of unigene 26797, a nested PCR assay was applied to isolate OcUGT1 from *O*. *caudatum* mRNA using gene-specific primers (Supplementary Table S1). An expected band with ca. 1.4 kb was generated and then inserted to *pEASY*^®^-Blunt to yield *pEASY*-OcUGT1 for sequencing (Supplementary Table S2). Sequence analysis showed its identity with the assembled unigene 26797, suggesting this sequence is a *bona fide* gene in *O*. *caudatum* genome. The sequence of unigene 26797 was then deposited in the GenBank database under accession number of MF621962. After sequence verification, comparison of predicted amino acid sequence of OcUGT1 with that of other GTs revealed identities of 57% with *Crocus sativus* UDP-glucose-dependent flavonoid glucosyltransferase (AIF79773.1), 56% with *Musa acuminata* subsp. *malaccensis* scopoletin glucosyltransferase-like (XP_009410978.1) and 55% with *Elaeis guineensis* scopoletin glucosyltransferase-like (XP_010925717.1). Sequence analysis revealed the presence of the Plant Secondary Product Glucosyltransferase (PSPG) signature sequence at the C-terminus of the predicted OcUGT1 protein, suggesting its involvement in secondary metabolism (Supplementary Figure S1A)^25,26^.

The phylogenetic tree containing the deduced amino acid sequence encoded by OcUGT1 and other reported microbial, plant and human GTs was then constructed. The phylogenetic tree was generated by the neighbor-joining method of the MEGA 5.0 program, using 1,000 bootstrap replications. The GenBank accession numbers of the sequences used in the phylogenetic analysis are listed in Supplementary Figure S1B. As shown in Supplementary Figure S1B, the GTs used in this study can be divided into two clades, flavonoid GTs and steroid GTs. Moreover, the flavonoid GTs were further grouped into three clusters. The first cluster contained flavonoid 3-*O*-glycosyltransferase (3-*O*-GT), the second cluster consisted of flavonoid 5-*O*-glycosyltransfrerase (5-*O*-GT) and the third group harbored flavonoid 7-*O*-glycosyltransferase (7-*O*-GT). OcUGT1 was grouped into the third cluster, displaying predicted 7-*O*-GT activity. Moreover, many GTs belonging to 7-*O*-GT cluster were reported to have the ability to glycosylated compounds with diverse structures, thereby suggesting OcUGT1 is likely to be catalytic promiscuity^27–29^.

### Heterologous expression and purification of OcUGT1

OcUGT1 was ligated with *Eco*RI/*Sal*I linearized pET-28a (+) to yield a recombinant plasmid pET28a-OcUGT1 for heterologous expression in *Escherichia coli* strain *Trans*etta (DE3) (TransGen, Beijing, China). After isopropyl-β-D-thiogalactoside (IPTG) induction, almost all of the recombinant OcUGT1 protein was found in an inclusion body form, suggesting the inappropriate aggregation of the recombinant OcUGT1 protein.

Molecular chaperones have long been known to function in preventing polypeptides from aggregation^30–32^. In order to improve the soluble expression of OcUGT1, the co-expression of pET28a-OcUGT1 and chaperone plasmid pKJE7 (Takara, Dalian, China) was performed in strain BL21 (DE3). The plasmid pKJE7 contained three genes, *dnaK*, *dnaJ* and *grpE*, each encoding a chaperone protein. Under the synergistic actions of these chaperone proteins, OcUGT1 was induced to express in *E*. *coli* in the soluble form (Supplementary Figure S2A). As shown in SDS-PAGE analysis, a specific OcUGT1 band with 53 kDa was present in the supernatant fraction of total protein (Supplementary Figure S2A). The expressed OcUGT1 protein was then purified to near homogeneity by affinity chromatography (Supplementary Figure S2B). The concentration of purified OcUGT1 was determined as 0.238 mg/ml using the procedure as described by Yin *et al*.^33^.

### OcUGT1-catalyzed glycosylation activities

**Figure 2:**
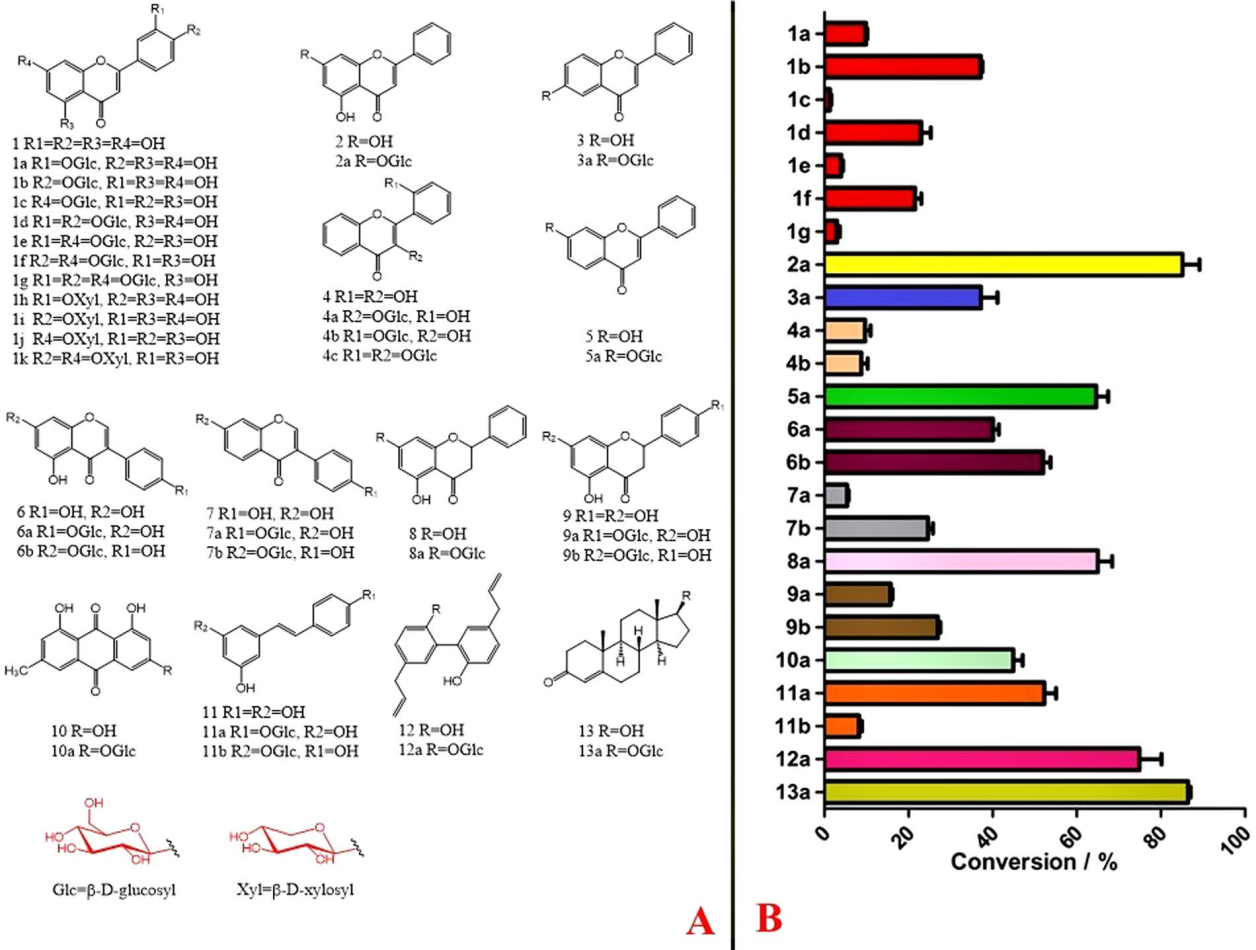
The catalytic promiscuity of OcUGT1-assisted glycosylation. (**A**) Chemical structures of sugar acceptors and corresponding glycosylated products; (**B**) Percent yields of glycosylated products catalyzed by OcUGT1. Data are expressed as the mean ± SE (standard error) of n = 3 independent determinations. All the reactions were conducted at 50 °C for 2 h. The HPLC conditions were provided in Supplementary Table S3.

**Figure 3:**
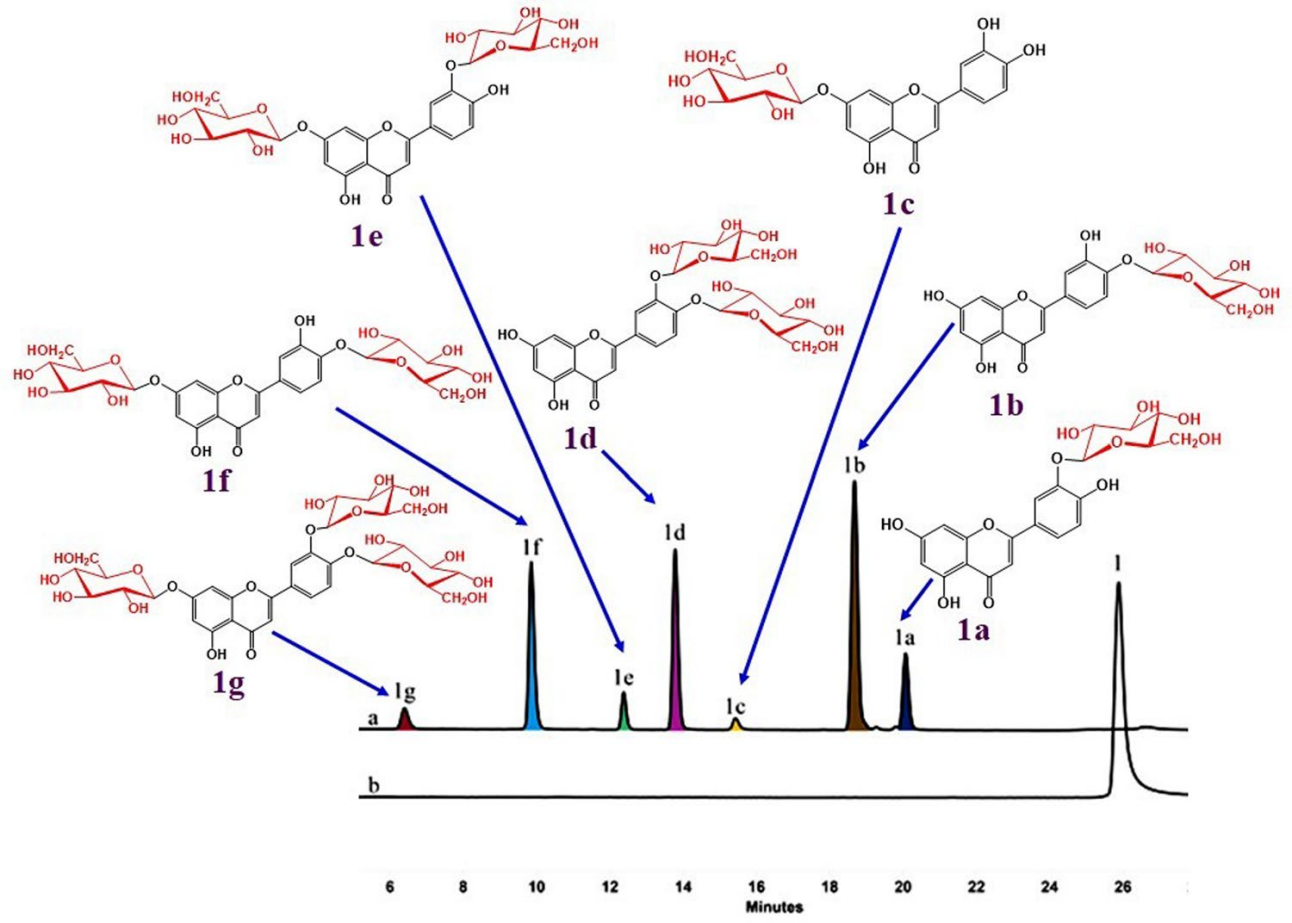
HPLC-profile of glycosylated products of luteolin (**1**) in the presence (**a**) or absence (**b**) of purified OcUGT1.

A total of 13 compounds with varied structures were used as sugar acceptors for glycosylation reactions (Fig. 2). OcUGT1 was predicted to display 7-*O*-GT activity. Chrysin (**2**) having two hydroxyl groups locating at the C-7 and C-5 positions was therefore used as the flavonoid substrate to test the glycosylation activity of OcUGT1 firstly. After incubation of chrysin (**2**) with the purified OcUGT1 protein for 2 h at 50 °C with a continue shaking, a new peak (marked with **2a**) was present but in contrast, the control showed no any conversion of chrysin (**2**), indicating the newly formed peak is a catalytic product of OcUGT1 (Supplementary result section I). The newly formed product was then analyzed by electrospray ionization-time of flight-mass spectrometry (ESI-TOF-MS) method. The ESI-MS spectrum of compound **2a** showed [M + HCOOH-H]^−^ peak at *m/z* 461.07, suggesting the molecular mass of this compound was 416, which was consistent with the introduction of a glucosyl group to the parent skeleton (Supplementary result section I). To further confirm the exact structure, 2 mg of this monoglucosylated chrysin was collected using HPLC and then used for NMR analysis. The metabolite was assigned to chrysin 7-glucoside (**2a**) based on ^1^H- and ^13^C-NMR spectra data (Supplementary result section I). The kinetic studies of OcUGT1-catalyzed glycosylation of chrysin (**2**) and UDP-Glc were then carried out. As shown in Supplementary Figure S3, the pH and temperature optimum were determined to pH 8 and 50 °C. The glycosylations of other substrates were thus performed under the optimal pH and temperature afterwards. Moreover, metal cations were observed to inhibit OcUGT1-catalyzed glycosylation reactions (Supplementary Figure S3C). The *K*_m_ and *V*_max_ values for chrysin (**2**) were 0.1506 ± 0.0139 mM and 0.2789 ± 0.0073 mM/h, respectively. The data clearly showed that OcUGT1 did encode a flavonoid 7-*O*-glucosyltransferase, regioselectively attacking the hydroxyl group at C-7 position. This notion was further verified by the glycosylation of luteolin (**1**) (Fig. 3), a flavonoid with multiple hydroxyl groups including a free 7-OH. The conversion of luteolin (**1**) showed seven newly formed metabolites **1a-1g (**Fig. 3**)**. The ESI-MS spectrum of compounds **1a**-**1c** showed [M-H]^−^ peak at *m/z* 447.1 (∆*m/z* of 162), indicating mono-glycosylated products (Supplementary result section II). In addition, three products (**1d**-**1f**) with [M-H]^−^ value of 609.2 revealed their di-glycosylated forms (∆*m/z* of 324) (Supplementary result section II). The minor product **1 g** showed a [M + HCOOH]^−^ value of 817.4 (Supplementary result section II), thus suggesting tri-glycosylation (∆*m/z* of 486). These metabolites were then assigned to luteolin 3′-*O*-glucoside(**1a**), luteolin 4′-*O*-glucoside (**1b**), luteolin 7-*O*-glucoside (**1c**), luteolin 3′,4′-*O*-diglucoside (**1d**), luteolin 7,3′-*O*-diglucoside (**1e**), luteolin 7,4′-*O*-diglucoside (**1 f**) and luteolin 7,3′,4′-*O*-triglucoside (**1 g**) based on their NMR spectra data, which were summarized in the Figures S4-13 and Tables S1-2 (Supplementary result section II). The formation of seven glucosides (**1a-1g**) indicated that in addition to the C-7 hydroxyl group, OcUGT1 was able to attach sugar to C-3′ and C-4′ hydroxyl groups of flavonoids, but not attack the hydroxyl group at C-5 position, consistent with the catalytic behavior towards chrysin (**2**). The catalytic results of OcUGT1 towards genistein (**6**) (Supplementary result section III) and daidzein (**7**) (Supplementary result section IV) further attested the catalytic preference of C-4′ and C-7 positions. Under the action of OcUGT1, both genistein (**6**) and daidzein (**7**) were glycosylated to yield a 4′-monoglucoside and a 7-monoglucoside, respectively (Supplementary result sections III and IV).

Also, OcUGT1 was observed to attack the hydroxyl group at C-6 position, catalyzing 6-hydroxyflavone (**3**) to form 6-hydroxyflavone 6-*O*-glucoside (**3a**, Supplementary result section V). Moreover, OcUGT1 could catalyze 2′-hydroxyflavonol (**4**) to generate three glucosides, including two monoglucosides (**4a**-**4b**, **[*M*** −** H]**^**−**^** = 415**.**2**) and a diglucoside (**4c**, **[*M* + H]**^**+**^** = 579**.**2)** (Supplementary result section VI). The compounds **4a** and **4b** were assigned as 2′-hydroxyflavonol 3-glucoside (**4a**) and 2′-hydroxyflavonol 2′-glucoside (**4b**) based on their respective NMR data (Supplementary result section VI). No exact structural identification of the diglucoside **4c** was carried out due to its trace amount (Supplementary result section VI). However, considering the attack preference of OcUGT1 towards the hydroxyl groups at C-2′ and C-3 positions of 2′-hydroxyflavonol (**4**), the compound **4c** was reasonably deduced to be 2′-hydroxyflavonol 3,2′-diglucoside (**4c**). The GTs targeting C-2′ position of flavonoids had never been documented^10^. OcUGT1 was therefore regarded as the first GT glycosylating C-2′ position of flavonoids. Overall, OcUGT1 was able to attach sugars to C-2′, C-3′, C-4′, C-6 and C-7 positions of flavones. Therefore, the monoglycosylated products of 7-hydroxyflavone (**5**), pinocembrin (**8**) and naringenin (**9**) catalyzed by OcUGT1 were reasonably deduced to 7-hydroxyflavone 7-*O*-glucoside (**5a**) (Supplementary result section VII), pinocembrin 7-*O*-glucoside (**8a**) (Supplementary result section VII), naringenin 4′-*O*-glucoside (**9a**) and naringenin 7-*O*-glucoside (**9b**) (Supplementary result section VII) according to the catalytic behavior of OcUGT1 toward flavones, as well as the corresponding HPLC profiles and LC-MS results (Supplementary result section VII). Besides flavonoids, OcUGT1 glycosylated other compounds, such as emodin (**10**) (Supplementary result section VIII), resveratrol (**11**) (Supplementary result section IX), magnolol (**12**) (Supplementary result section X) and testosterone (**13**) (Supplementary result section XI), suggesting the acceptor promiscuity of OcUGT1 (Fig. 2). The percentage yields of OcUGT1 towards the 13 compounds were thus calculated (Fig. 2B). As shown in Fig. 2B, OcUGT1 had the highest glycosylation efficiency for **2** and **13**, approaching 90% (Fig. 2B).

**Figure 4:**
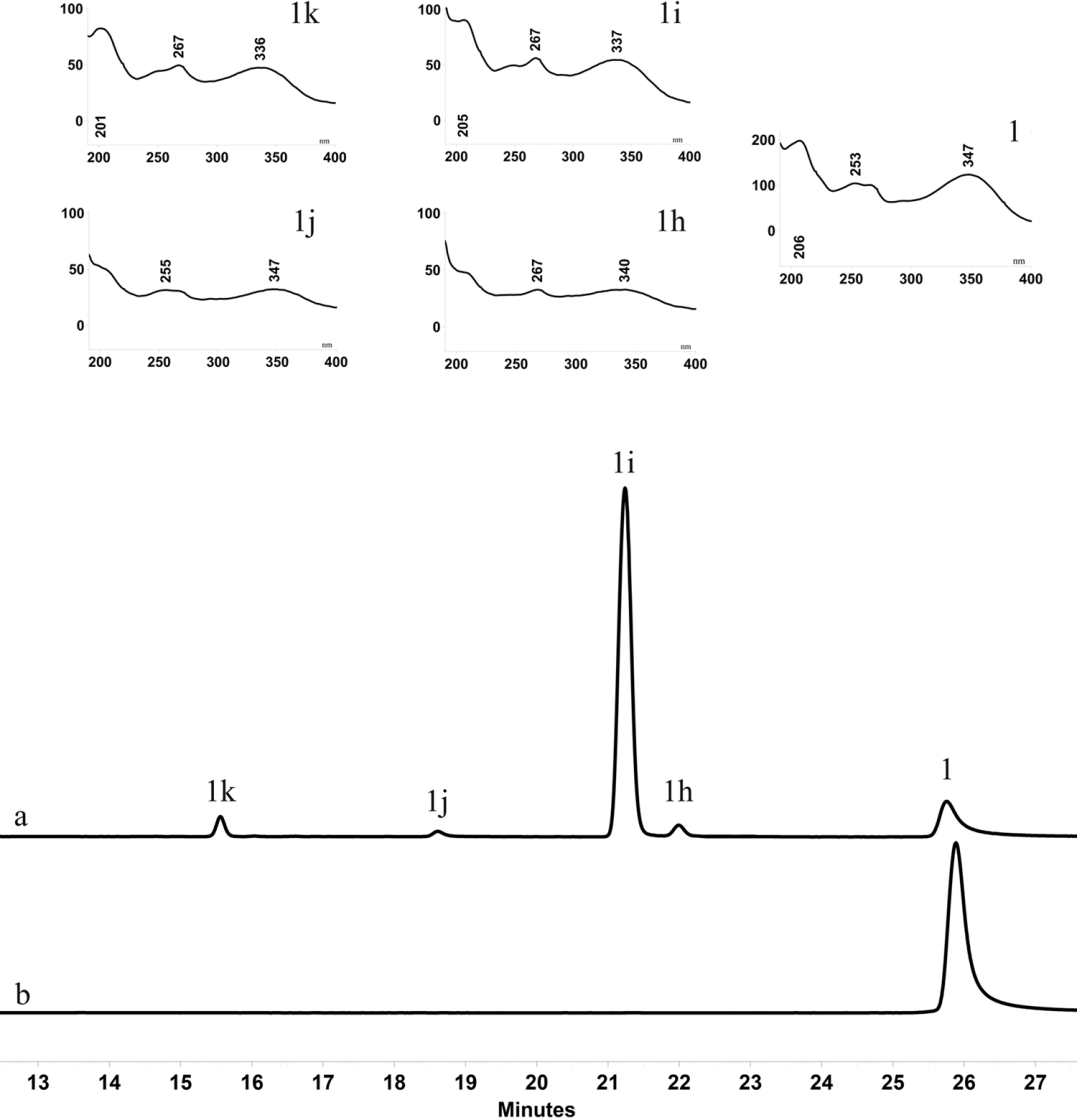
HPLC chromatogram of xylosylated metabolites (**1h-1k**) of luteolin (**1**) with OcUGT1 (**a**) or without OcUGT1 (**b**). The UV absorption spectrum of **1h-1k** are similar to that of **1**. All of them are marked in the top panels.

Besides the diversity of sugar acceptors, OcUGT1 displayed sugar donor promiscuity. As shown in Fig. 4, luteolin (**1**) was xylosylated to form four luteolin derivatives, including three monoxylosylated (**1h-1j**, each with [M − H]^−^ = 417.2) and one dixylosylated metabolites (**1k**, [M − H]^−^ = 549.3) (Supplementary result section II). The four metabolites were then assigned to luteolin 3′-*O*-xyloside(**1 h**), luteolin 4′-*O*-xyloside (**1i**), luteolin 7-*O*-xyloside (**1j**) and luteolin 7,4′-*O*-dixyloside (**1k**) according to their NMR data (Supplementary result section II). Overall, OcUGT1 is a glycosyltransferase with catalytic promiscuity, attaching sugars to hydroxyl groups of varied compounds.

### Reversible reactions of GT-catalyzed glycosylations

The reversibility of GT-catalyzed glycosylations was attested previously^6^. It has been well accepted that GTs can catalyze three reversible reactions, namely NDP-sugar synthesis, sugar exchange and aglycon exchange^6^. Therefore, we also tested whether OcUGT1 could perform three reversible reactions in this study, using the compounds listed in Supplementary result section XII as the substrates.

### NDP-sugar synthesis

**Figure 5:**
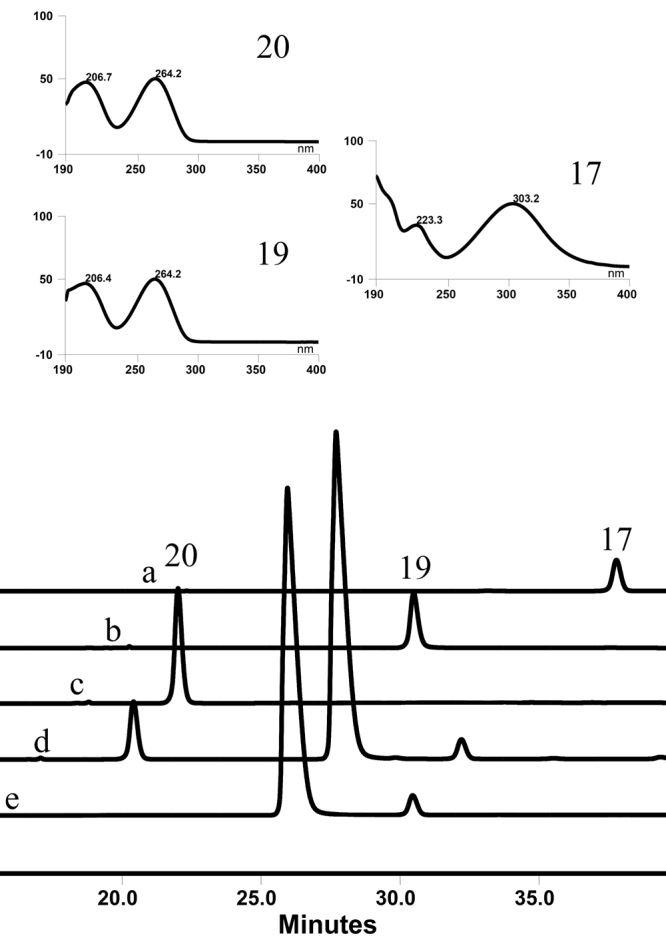
OcUGT1-catalyzed UDP-Glc (**20**) synthesis using *p*NP-β-Glc (**17**) and UDP (**19**) as the substrates. (**a**) *p*NP-β-Glc (**17**) standard (1 mM); (**b**) UDP (**19**) standard (0.2 mM); (**c**) UDP-Glc (**20**) standard (0.5 mM); (**d**) reaction mixture with OcUGT1 (**e**) the control reaction without OcUGT1. The UV absorption spectra of **17**, **19** and **20** are marked in the top panels.

As shown in Fig. 5, when *p*-Nitrophenyl-β-D-glucopyranoside (*p*NP-β-Glc, **17**) and UDP (**19**) were incubated with OcUGT1, the glucosyl group from *p*NP-β-Glc (**17**) was transferred to UDP (**19**), thereby forming UDP-Glc (**20**) under the action of OcUGT1 (Fig. 5). On the other hand, if no OcUGT1 was mixed with *p*NP-β-Glc (**17**) and UDP (**19**), no any new peaks were produced (Fig. 5e). These evidences indicated that OcUGT1 did catalyze the synthesis of UDP-Glc (**20**). Moreover, OcUGT1 were observed to be promiscuous towards glycosyl donors, as exemplified in Supplementary result section XII, in which the glycosyl group from *o*-Nitrophenyl-β-D-glucopyranoside (*o*NP-β-Glc, **16**) or *o*-Nitrophenyl-β-D-xylopyranoside (*o*NP-β-Xyl, **15**) was transferred to UDP(**19**), leading to the synthesis of corresponding UDP-sugars, namely UDP-Glc (**20**) and UDP-Xyl (**21**) (Supplementary result section XII).

### Sugar exchange

**Figure 6:**
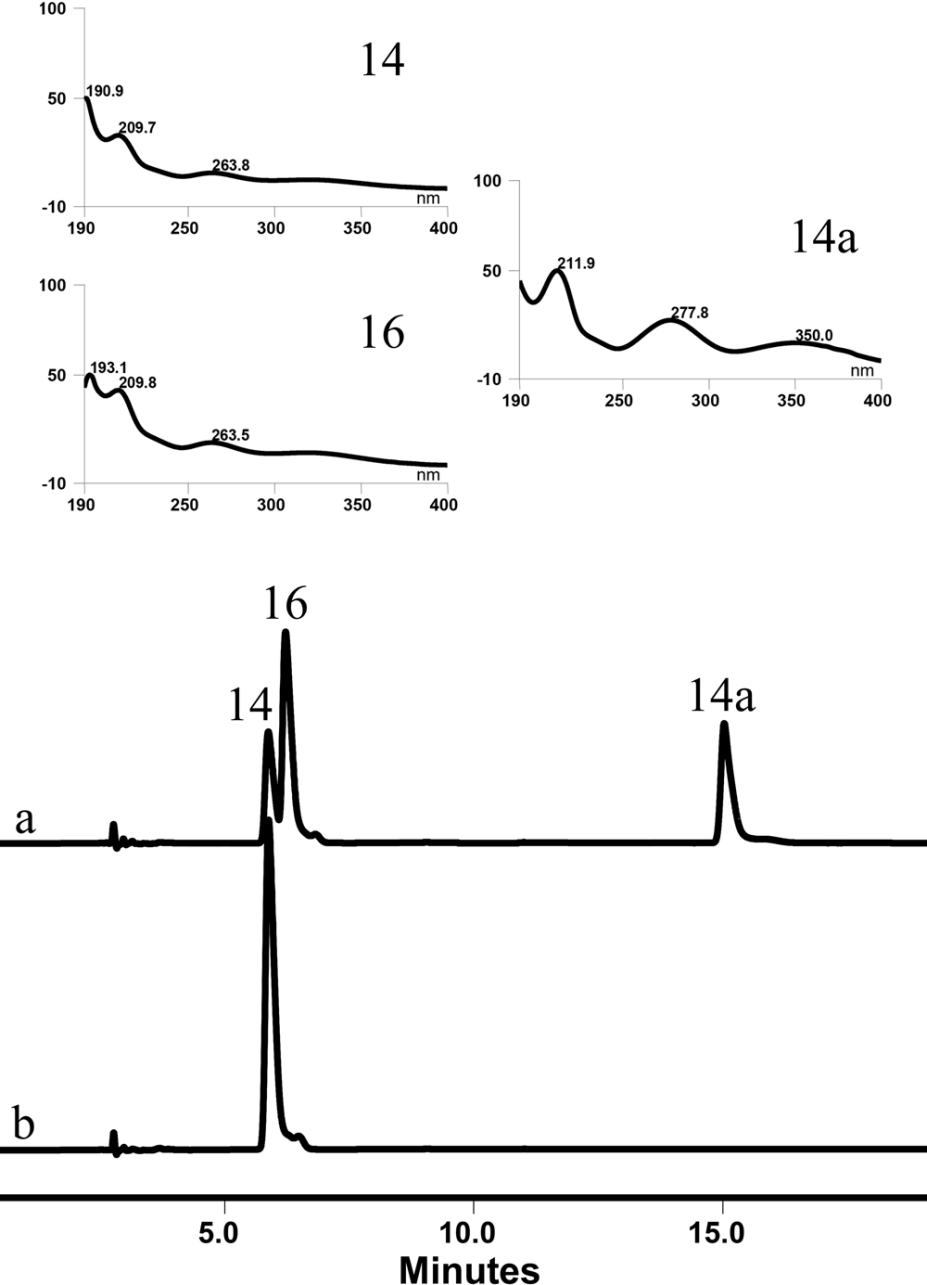
Sugar exchange between *o*NP-β-Gal (**14**) and UDP-Glc (**20**) catalyzed by OcUGT1 (**a**) or no OcUGT1 (**b**). The UV absorption spectra of **14**, **14a** and **16** are marked in the top panels.

OcUGT1 performs sugar exchange reactions, as illustrated in Fig. 6. In this sugar exchange reaction, the galactosyl group from *o*-Nitrophenyl-β-D-galactopyranoside (*o*NP-β-Gal, **14**) was exchanged with the glucosyl group on UDP-Glc (**20**), forming *o*NP-β-Glc (**16**) (Fig. 6). Moreover, a new peak representing *o*NP (**14a**) was present in this sugar exchange reaction, suggesting the hydrolysis of *o*NP-β-Gal (**14**) occurred simultaneously during the sugar transfer reaction (Fig. 6). Also, OcUGT1-catalyzed sugar exchange occurred between *o*NP-β-Xyl (**15**) and UDP-Glc (**20**), generating *o*NP-β-Glc(**16**) (Supplementary result section XII).

### Aglycon exchange

**Figure 7:**
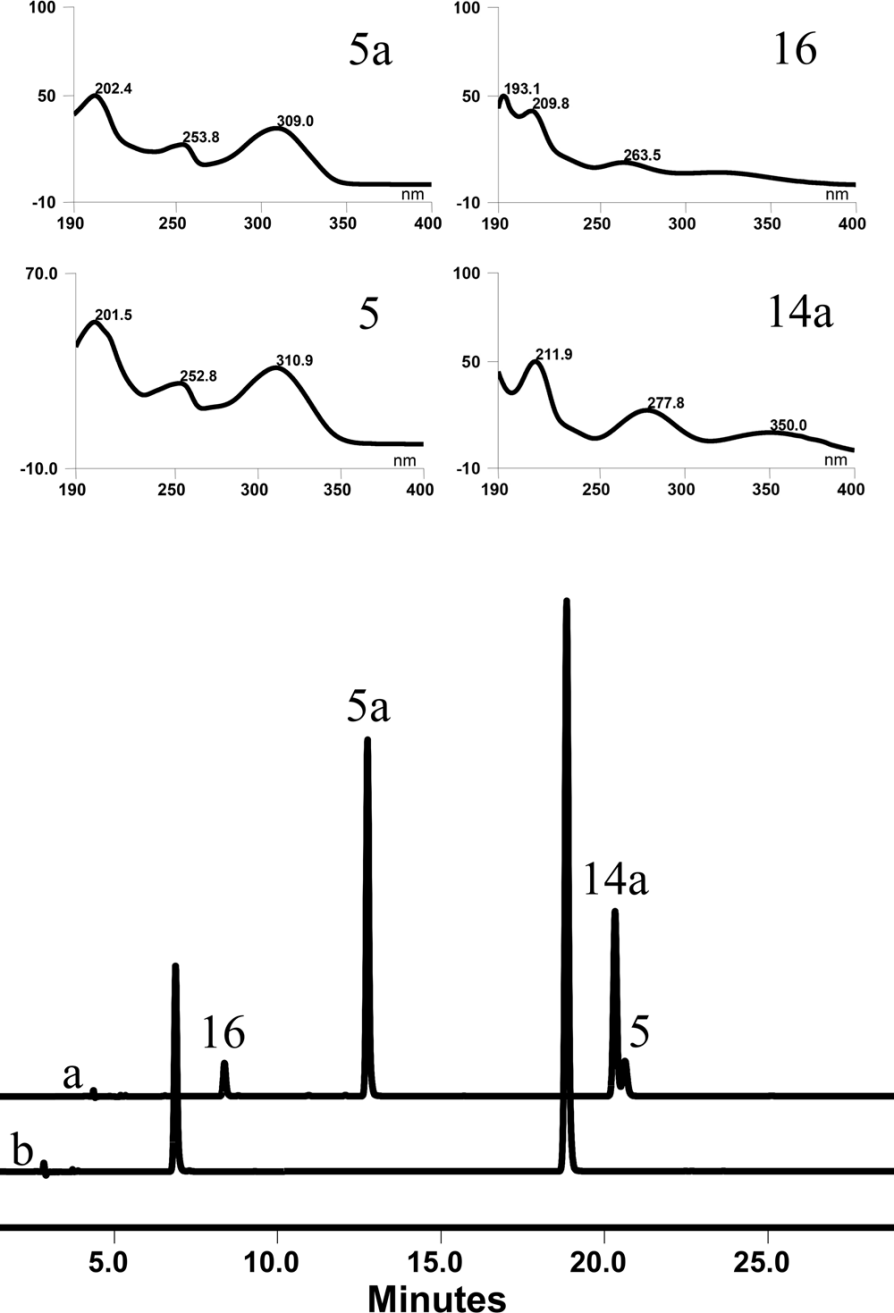
Aglycon exchange between *o*NP-β-Glc (**16**) and 7-hydroxyflavone (**5**) catalyzed by OcUGT1 (**a**) or no OcUGT1 (**b**). The UV absorption spectra of **5**, **5a**, **16** and **14a** are marked in the top panels.

The catalytic ability of OcUGT1-assisted aglycon exchange was also verified. When the reaction mixture containing *o*NP-β-Glc (**16**) and 7-hydroxyflavone (**5**) (Fig. 7) was incubated with OcUGT1 at 37 °C for 2 h, new peaks representing *o*NP (**14a**) and 7-hydroxyflavone 7-*O*-glucoside (**5a**) were present in the reaction mixtures. On the contrary, no any new peaks were present in the control mixture having no purified OcUGT1. These data indicated aglycon exchange between *o*NP-β-Glc (**16**) and 7-hydroxyflavone (**5**) occurred under the action of OcUGT1 (Fig. 7). Also, OcUGT1-assisted aglycon exchange can occur between *p*NP-β-Glc (**17**) and 7-hydroxyflavone (**5**), generating two new products *p*NP (**17a**) and 7-hydroxyflavone 7-*O*-glucoside (**5a**) (Supplementary result section XII).

### Glycosidase activity of OcUGT1

Besides monoglycosides (**1a-1c**), OcUGT1 converted luteolin (**1**) into di-glycosides (**1d-1f**) and a tri-glycoside (**1 g**), suggesting OcUGT1 had the ability to accept glycosylated metabolites for further sugar attachments. To probe this notion, seven luteolin glucosides (**1a-1g**) were applied as the substrate of OcUGT1 to react with UDP-Glc in phosphate buffer (10 mM, pH 8.0) at 50 °C for 2 h. As expected, each mono-glucoside (**1a**, **1b** or **1c**) was further glycosylated to form two di-glucosides and one tri-glucoside, while all of the three di-glucosides (**1d-1f**) were further glycosylated to yield the tri-glycoside **1 g** (Supplementary result section XII). Surprisingly, in addition to glycosylation, we observed OcUGT1 could catalyze two other reactions, namely hydrolysis and trans-glycosylation reactions. As shown in Supplementary result section XII, the luteolin tri-glucoside **1 g** was hydrolyzed to generate two di-glucosides **1d** and **1e**. Likewise, di-glucoside **1d** could be hydrolyzed to produce mono-glucoside **1a**, while another di-glucoside **1 f** was hydrolyzed to form a mono-glucoside **1b**. Moreover, OcUGT1 was found to display trans-glycosylation activity. The glycosyl residue at position 4′ of **1b** was transferred to position 3′ or *O*7 by intra-molecular trans-glycosylation, thereby forming luteolin 3′-*O*-glucoside (**1a**) or luteolin 7-*O*-glucoside (**1c**). Likewise, OcUGT1-catalyzed trans-glycosylation from **1c** to **1b** or from **1d** to **1e** was also observable (Supplementary result section XII). These evidences, together with OcUGT1-assisted hydrolysis of *o*NP-β-Gal (**14**) as illustrated in Fig. 6, revealed that OcUGT1 displayed glycosidase activity, performing hydrolysis and trans-glycosylation reactions.

### Hydrolytic activity of OcUGT1

**Figure 8:**
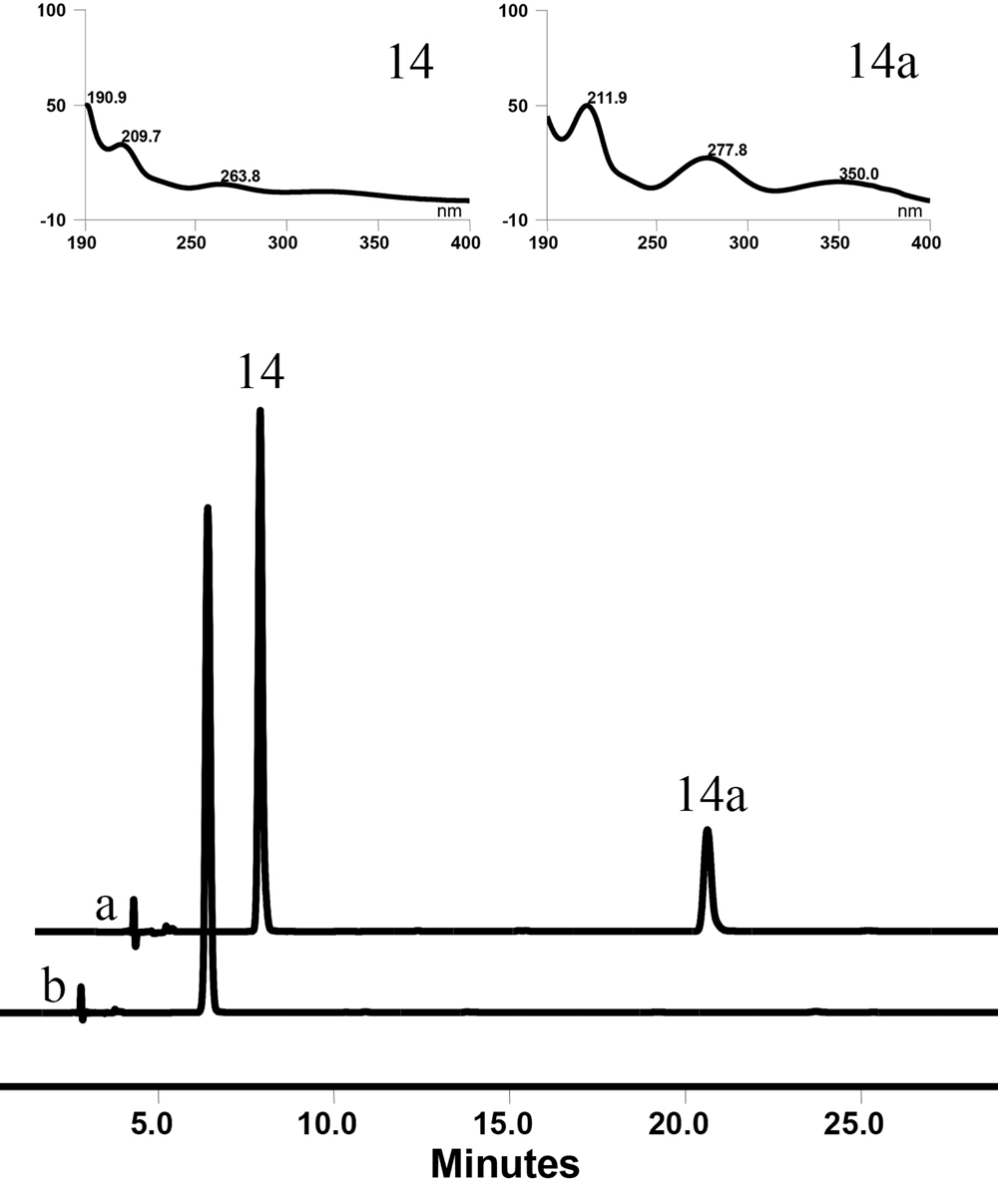
OcUGT1-assisted hydrolysis toward *o*NP-β-Gal (**14**). (**a**) HPLC chromatogram of OcUGT1-catalyzed hydrolysis from *o*NP-β-Gal (**14**) to *o*NP (**14a**). (**b**) HPLC chromatogram of the standard 14. UV spectra of **14** and its hydrolytic product **14a** are shown in upper panels.

To further verify the hydrolytic action of OcUGT1, more evidences were provided. Firstly, *o*NP-β-Gal (**14**) was used as the hydrolytic substrate to react with purified OcUGT1. After incubation at 37 °C for 2 h, *o*NP-β-Gal (**14**) was hydrolyzed to aglycon *o*NP (**14a**) (Fig. 8). The optimal pH and temperature for hydrolysis were thus determined to pH 7 and 37 °C (Supplementary Figures S3D and S3E). Also, the kinetic parameters *K*_m_ and *V*_max_ of OcUGT1 toward **14** were calculated to 6.897 ± 1.995 mM and 3.332 ± 0.862 mM/h. Moreover, OcUGT1 was found to be catalytic flexibility, hydrolyzing varied glycosides including chrysin-7-glucoside (**2a**), *o*NP-β-Xyl (**15**), *o*NP-β-Glc (**16**), *p*NP-β-Glc(**17**) and *p*-Nitrophenyl-α-D-glucopyranoside (*p*NP-α-Glc, **18**) (Supplementary result section XII) to corresponding aglycon and monosaccharide.

### Trans-glycosylation activity of OcUGT1

**Figure 9:**
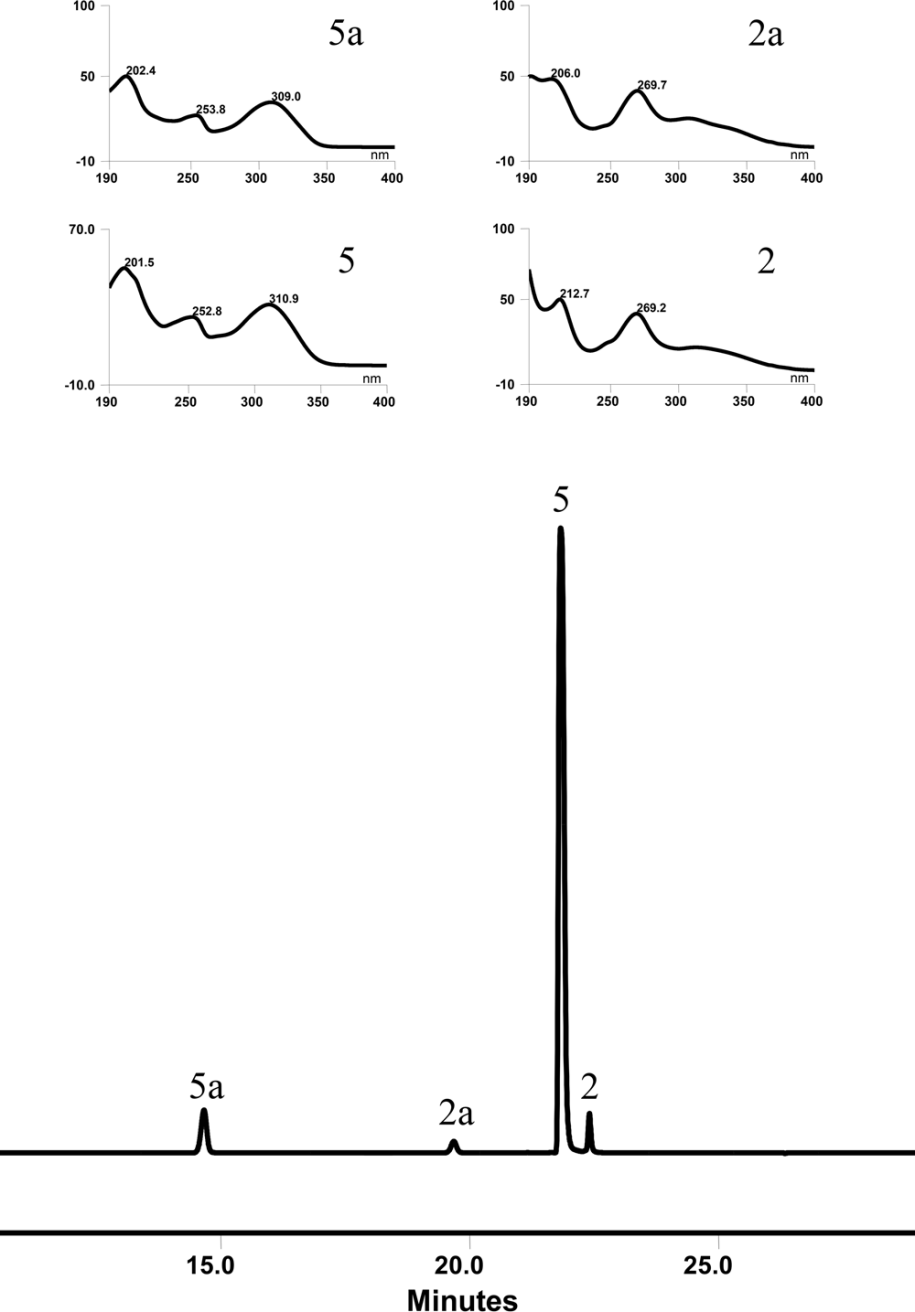
OcUGT1-catalyzed intermolecular transglycosylation between chrysin-7-glucoside (**2a**) and 7-hydroxyflavone (**5**). UV spectrum of substrates **2a** and **5** and products **2** and **5a** are shown in upper panels.

It was generally accepted that glycosidases possessed at least two types of trans-glycosylation actions, namely intra-molecular^34^ and intermolecular trans-glycosylation^35^. The intermolecular trans-glycosylation activity of OcUGT1 was thus firstly investigated using chrysin-7-*O*-glucoside (**2a**) as sugar donor and 7-hydroxyflavone (**5**) as the acceptor. As shown in Fig. 9, OcUGT1 catalyzed an intermolecular trans-glycosylation reaction by transferring a glycosyl moiety from chrysin-7-glucoside (**2a**) to the acceptor 7-hydroxyflavone (**5**), thereby forming two new products chrysin (**2**) and 7-hydroxyflavone 7-*O*-glucoside (**5a**) (Fig. 9). The effects of pH, temperature and metal ions on trans-glycosylation activity of OcUGT1 toward chrysin-7-*O*-glucoside (**2a**) were studied. As shown in Supplementary Figure S3, OcUGT1 exhibited substantial activity at temperatures from 0 to 70 °C and displayed the highest activity at 37 °C. OcUGT1 showed a wide pH tolerance and was optimally active at pH 6.0. EDTA, Ca^2+^ or Li^+^ had a significantly stimulatory effect on transglycosylation activity. On the other hand, when 5 mM Mn^2+^, Mg^2+^, Zn^2+^, Co^2+^ or Cu^2+^ were included in reaction mixtures, the inhibitory action on OcUGT1 activity was observable (Supplementary Figure S3).

Moreover, OcUGT1 displayed catalytic versatility, catalyzing intermolecular trans-glycosylations from *o*NP-β-Glc (**16**) to 6-hydroxyflavone (**3**), from *p*NP-β-Glc(**17)** to 6-hydroxyflavone(**3**), from chrysin-7-glucoside (**2a**) to 6-hydroxyflavone (**3**), from genistein-4′-glucoside (**6a**) to 7-hydroxyflavone (**5**) or from genistein-7-glucoside (**6b**) to 7-hydroxyflavone (**5**) (Supplementary result section XII). Surprisingly, in verifying the intermolecular trans-glycosylation from genistein-7-glucoside (**6b**) to 7-hydroxyflavone (**5**), we observed the intramolecular trans-glycosylation of genistein-7-glucoside (**6b**). As illustrated in Supplementary result section XII, the glucosyl group at position *O*7 of genistein-7-glucoside (**6b**) was transferred to position 4′, generating genistein-4′-glucoside (**6a**). It was therefore obvious that there were three metabolites, **6**, **6b** and **5a**, in the reaction mixtures (Supplementary result section XII). These evidences unambiguously indicated that OcUGT1 could catalyze trans-glycosylations, forming new glycosides without the addition of NDP-sugars.

### Expression profile of OcUGT1 in *O. caudatum*

To explore the *in vivo* activities of OcUGT1, the expression profile of OcUGT1 was determined by quantitative real-time reverse transcription PCR (qRT-PCR). As shown in Supplementary Figure S4, transcripts of OcUGT1 were detected in all tissues tested. OcUGT1 was most expressed in bulbs followed by flowers, alabastrums, bulblets, roots, sterile bulbs and leaves. The transcripts in bulbs were approximately 10-fold higher than that in leaves of *O*. *caudatum* (Supplementary Figure S4). The bulblet is a small bulb. The transcripts of OcUGT1 in bulbs and bulblets varied differentially, suggesting the *in vivo* expression of OcUGT1 was development-dependent (Supplementary Figure S4). Sterile bulbs are bulbs cultivated in sterile environment. Significant variation in mRNA expression of OcUGT1 was observed between bulbs and sterile bulbs, providing a clue that the expression of OcUGT1 was highly affected by environmental factors (Supplementary Figure S4).

## Conclusions

GTs and glycosidases have long been thought to be two classes of proteins. Herein, in an effort to characterize flexible GTs, we unexpectedly found a flavonoid GT, designated as OcUGT1 from *O*. *caudatum*, with glycosidase activity (Fig. 1).

*In vitro* enzymatic results show that OcUGT1 is a multifunctional enzyme that catalyzes at least seven reactions, namely classical sugar transfer reaction, NDP-sugar synthesis, sugars exchange, aglycons exchange, intra-molecular trans-glycosylation and intermolecular trans-glycosylation and hydrolysis reactions. OcUGT1-catalyzed trans-glycosylations allow the formation of glycosides without the additions of expensive NDP-sugars or NDP. Undoubtedly, the unusual multifunctionality of OcUGT1 broadens its applicability.

Further results indicate that OcUGT1 is a substrate-promiscuous glycosyltransferase, acting on diverse substrates in each reaction. Moreover, OcUGT1 was verified as the first GT glycosylating C-2′ position of flavonoids. The availability of substrate-promiscuous glycosyltransferases was thus crucial to the success of glycodiversification of natural products.

Expression profile revealed that OcUGT1 was expressed in a development-dependent manner. Moreover, the expression of OcUGT1 was influenced by the environmental factors.

## Experimental

### Plant materials

Plant cultivation of *O*. *caudatum* was conducted as usual. RNA samples, isolated from fresh tissues including root, bulb, leaf, flowers, bulblet, alabastrum and sterile bulb, were used as the template for qRT-PCR. Moreover, the sterile bulbs of *O*. *caudatum* were inoculated on 6, 7-V medium were used as the start material for total RNA isolation, thereby providing templates for cDNA isolation and qRT-PCR analysis towards OcUGT1^22,23,33,36^.

### RNA-Seq data analysis and retrieval of unigenes encoding glycosyltransferase

The *O*. *caudatum* transcriptome was sequenced by use of Illumina HiSeq™ 2000 sequencing platform previously in our laboratory^24,37–39^. The unigenes sharing high identity with flavonoids GTs and having a complete ORF were thus retrieved from the transcriptome dataset, as described previously^22,23^. A bioinformatics analysis on candidate unigenes was then performed as introduced by Li *et al*.^23^ and Yin *et al*.^22^.

### cDNA isolation, heterologous expression and protein purification

To verify the authenticity of candidate unigenes, nested PCR using the gene-specific primers (Supplementary Table S1) was performed to isolate cDNA from *O*. *caudatum* total RNA. The subsequent sequence verification, pET-28a (+) derived vector construction, protein expression and purification were carried out as reported previously^22,33,36,40,41^. The plasmids and strains used in this study were summarized in Supplementary Table S2.

### OcUGT1-catalyzed glycosylation

The uridine diphosphate (UDP)-D-glucose (UDP-Glc), UDP-D-glucuronic acid (UDP-GlcA), UDP-D-galactose (UDP-Gal), UDP-D-xylose (UDP-Xyl) and UDP-N-acetyl-D-glucosamine (UDP-GlcNAc) were applied as sugar donors. UDP-Glc, UDP-GlcA, UDP-Gal and UDP-GlcNAc were purchased from Sigma–Aldrich. UDP-Xyl was enzymatically prepared from UDP-GlcA using recombinant xylose synthase (GenBank accession number, KT757272) as the catalyst^40^.

The reaction mixture for glycosylation contained 10 mM phosphate buffer (pH 8.0), 2 μg purified OcUGT1, 1 mM sugar donor and 1 mM sugar acceptor in a total volume of 100 μl. All the GT activity assays were conducted at 50 °C for 2 h and then quenched by adding 100 μl methanol and 10 μl acetic acid. The glycosylated products were monitored by HPLC chromatography (Supplementary Table S3). The exact structure of these glycosylated products was further elucidated by combinational usage of HPLC-UV, HPLC-MS and NMR, as described previously^23,37,40,42^.

The effects of pH, temperature and metal ions on OcUGT1 activity were examined under the assay conditions. The temperature optima of the recombinant enzymes were determined by incubating reaction mixtures in phosphate buffer (pH 6–9) at different temperatures ranging from 0 to 80 °C with 10 °C intervals. The pH varied between 4–11 by using sodium acetate-acetic acid buffer (0.1 M, pH 4–6), phosphate buffer (0.1 M, pH 6–9) and Tris-HCl buffer (0.1 M, pH 9–11). The influence of metal ion was also investigated using the standard reaction mixture in the presence of 5 mM EDTA, MnCl_2_, LiCl, MgCl_2_, CaCl_2_, CoCl_2_, ZnCl_2_ and CuCl_2_. The kinetic constants (*K*_m_ and *V*_max_ values) for chrysin (**2**) (varied between 0 and 1 mM) at a fixed concentration of 1 mM UDP-Glc were determined in phosphate buffer (pH 8.0) at 50 °C. The apparent *K*_m_ and *V*_max_ values were estimated from Lineweaver-Burk plots.

### Reversible reactions of OcUGT1-catalyzed glycosylation

Three GT-catalyzed reversible reactions, namely NDP-sugars synthesis, sugar exchange and aglycon exchange, were reported previously^6^. In this contribution, OcUGT1-catalyzed reversible reactions were measured in the reaction mixture of 100 μl, which contained 0.1 M phosphate buffer (pH 6.0), 2 μg purified OcUGT1 and 1 mM each substrate. The reaction mixture was incubated at 37 °C for 2 h and then terminated by adding 100 μl methanol and 10 μl acetic acid.

### Hydrolysis assay of OcUGT1

The hydrolysis activity was initially developed using *o*NP-β-Gal (**14**) as the substrate. The reaction mixture with a total volume of 100 μl containing 10 μl phosphate buffer (0.1 M, pH 6.0), 2 μg purified OcUGT1, 10 μl *o*NP-β-Gal (10 mM) were incubated at 37 °C for 2 h. The reaction was stopped by adding 100 μl methanol and 10 μl acetic acid. The hydrolytic activity was determined by measuring *o*-nitrophenol (*o*NP, **14a**) release using HPLC. The effects of pH, temperature and metal ions on hydrolytic capacity were examined as described above, in the pH range from 4 to 11 and in the temperature range 0–70 °C. The kinetic parameters for hydrolytic activity were calculated as mentioned above. Moreover, the catalytic flexibility of OcUGT1 using other glycosides (Supplementary Figure S1) as the substrates was investigated at optimal pH and temperature, as described above.

### Trans-glycosylation assay of OcUGT1

The trans-glycosylation activities of OcUGT1 were assayed in 100 μl of 10 mM phosphate buffer (pH 6.0), 2 μg purified OcUGT1, 1 mM glycoside and 1 mM aglycon. The reaction mixture was incubated at 37 °C for 2 h and then 100 μl methanol and 10 μl acetic acid were added to terminate the trans-glycosylation reaction. The resulted products were analyzed by HPLC. HPLC conditions were provided in Supplementary Table S3. The enzymatic properties of OcUGT1 towards trans-glycosylation action, such as optimal pH and temperature, the influence of metal ions and the kinetic constants, were evaluated as reported above.

### Tissue-specific expression analysis of OcUGT1 in *O. caudatum*

The distribution of OcUGT1 transcripts in various tissues, including root, bulb, leaf, flowers, bulblet, alabastrum and sterile bulb, was examined by qRT-PCR as described previously^22,23^. The transcript of GAPDH2 (GenBank accession no. KM370884) was used as an internal control to normalize the expression levels. The relative expression of OcUGT1 was calculated by the 2^−ΔΔCT^ method as described by Yin *et al*.^22^ and Li *et al*.^23^. All qRT-PCR experiments were performed in triplicate. Primers suitable for qRT-PCR analysis of OcUGT1 are listed in Supplementary Table S1.

## Electronic supplementary material

Supplementary information
