# Supplementary material for: Isolation and characterization of a multifunctional flavonoid glycosyltransferase from Ornithogalum caudatum with glycosidase activity
Source: Sci Rep. 2018 Apr 12;8:5886. doi: 10.1038/s41598-018-24277-3 (PMC5897352; doi:10.1038/s41598-018-24277-3)
Supplement: Supplementary file 1 — Supplementary information [file 41598_2018_24277_MOESM1_ESM.pdf]

Isolation and characterization of a multifunctional flavonoid glycosyltransferase from *Ornithogalum caudatum* with glycosidase activity

Shuai Yuan[1], Sen Yin[1], Ming Liu[1], Jian-Qiang Kong[1,*]

[1]Institute of Materia Medica, Chinese Academy of Medical Sciences & Peking Union Medical College (State Key Laboratory of Bioactive Substance and Function of Natural Medicines & Ministry of Health Key Laboratory of Biosynthesis of Natural Products), Beijing, 100050, China

*, Author to whom correspondence should be addressed.

E-mail: jianqiangk@imm.ac.cn

Supplementary result section I

Section I is a database presenting HPLC chromatogram (Figure S1), UV spectrum (Figure S1), mass spectrum (Figure S1) and NMR spectrum (Figure S2 and Table S1) for compound **2a**.

Table S1 ^{1}H NMR (600 MHz) and ^{13}C NMR (150 MHz) spectroscopic data (δ in ppm) for **2a** in DMSO-d_6

Figure S1 HPLC chromatogram of glycosylated metabolite (**2a**) of chrysin (**2**) with OcUGT1 (a) or without OcUGT1 (b). The UV absorption spectrum of **2a** is similar to that of **2**. Both are marked in the top panels. The mass spectrum of **2a** analyzed on ESI-TOF MS, as shown in the red panel.

Figure S2 ^{1}H NMR spectrum (600 MHz, DMSO-d_6) (A) and ^{13}C NMR spectrum of **2a** (150 MHz, DMSO-d_6) (B)

Table S1

Position	δC	δH
2	163.7, C	
3	105.6, CH	7.08, 1H, s
4	182.2, C	
5	161.1, C	
6	99.7, CH	6.48, 1H, d(2.1Hz)
7	163.2, C	
8	95.0, CH	6.89, 1H, d(2.1Hz)
9	157.1, C	
10	105.5, C	
1'	130.6, C	
2'	126.5, CH	8.12, 1H, overlapped
3'	129.2, CH	7.64, 1H, m
4'	132.2, CH	7.64, 1H, m
5'	129.2, CH	7.64, 1H, m
6'	126.5, CH	8.12, 1H, overlapped
1"	99.9, CH	5.09, 1H, d(7.4Hz)
2"	73.1, CH	
3"	76.4, CH	
4"	69.5, CH	
5"	77.2, CH	
6"	60.6, CH_2	6"α 3.72, 1H, d(10.0Hz)
		H of Sugar 3.10-3.50

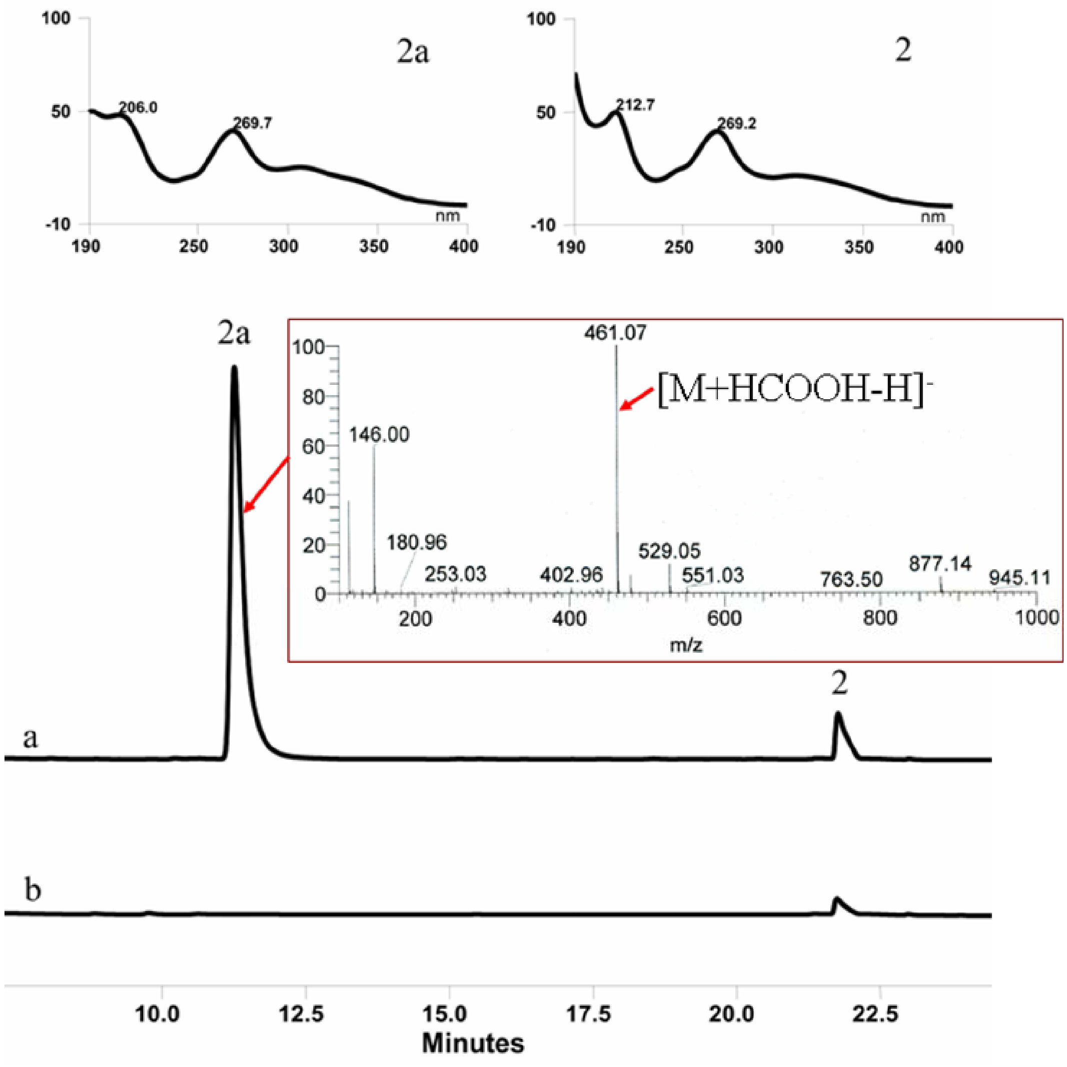
2a
206.0
269.7
nm
2
212.7
269.2
nm
461.07
[M+HCOOH-H]-
146.00
180.96
253.03
402.96
529.05
551.03
763.50
877.14
945.11
m/z
a
b
Minutes

Figure S1

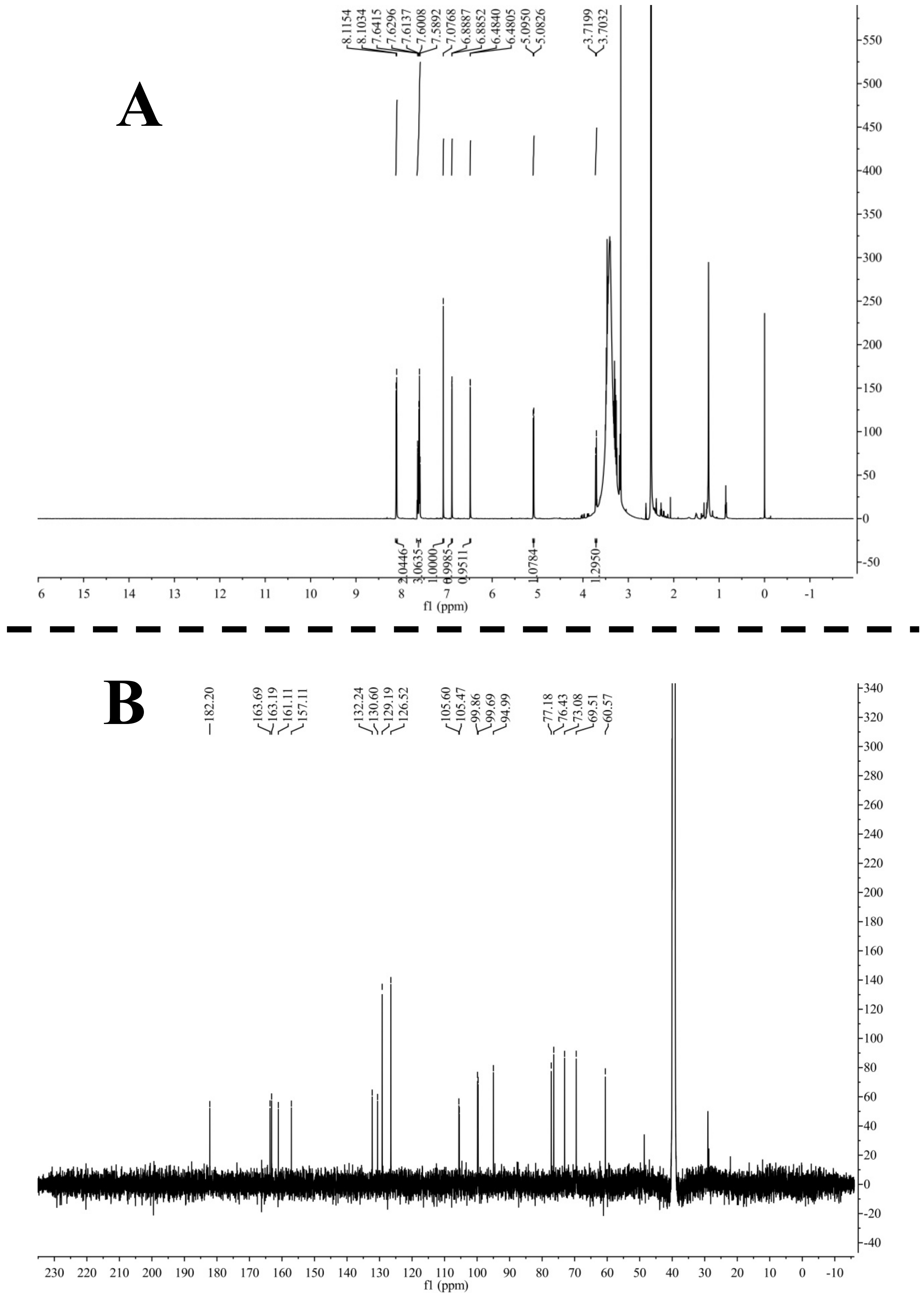
A
8.1154
8.1034
7.6415
7.6296
7.6137
7.6008
7.5892
7.0768
6.8887
6.8852
6.4840
6.4805
5.0950
5.0826
3.7199
3.7032
2.0446
3.0635
1.0000
0.9985
0.9511
1.0784
1.2950
f1 (ppm)
B
182.20
163.69
163.19
161.11
157.11
132.24
130.60
129.19
126.52
105.60
105.47
99.86
99.69
94.99
77.18
76.43
73.08
69.51
60.57
f1 (ppm)

Figure S2

Supplementary result section II

Section II is a database presenting mass spectrum (Figures S1-4) and NMR spectrum (Figures S5-20 and Tables S1-4) for compounds **1a−1k**.

Table S1 ^{1}H NMR (600 MHz) spectroscopic data (*δ* in ppm, *J* in Hz) for compounds **1a-1g** in DMSO-d_6

Table S2 ^{13}C NMR (150 MHz) spectroscopic data (*δ* in ppm) for compounds **1a−1g** in DMSO-d_6

Table S3 ^{1}H NMR (600 MHz) spectroscopic data (*δ* in ppm, *J* in Hz) for compounds **1h−1k** in DMSO-d_6

Table S4 ^{13}C NMR (150 MHz) spectroscopic data (*δ* in ppm) for compounds **1h−1k** in DMSO-d_6

Figure S1 The mass spectra of **1a** (**A**), **1b** (**B**) and **1c** (C) analyzed on ESI-TOF MS

Figure S2 The mass spectra of **1d (A), 1e (B)** and **1f (C)** analyzed on ESI-TOF MS

Figure S3 The mass spectra of **1g** analyzed on ESI-TOF MS

Figure S4 The mass spectra of **1h (A), 1i (B), 1j (C)** and **1k** (**D**) analyzed on ESI-TOF MS

Figure S5 ^{1}H NMR spectrum (600 MHz, DMSO-d_6) (**A**) and ^{13}C NMR spectrum of **1a** (150 MHz, DMSO-d_6) (**B**)

Figure S6 ^{1}H NMR spectrum (600 MHz, DMSO-d_6) (**A**) and ^{13}C NMR spectrum of **1b** (150 MHz, DMSO-d_6) (**B**)

Figure S7 ^{1}H NMR spectrum (600 MHz, DMSO-d_6) (**A**) and ^{13}C NMR spectrum of **1c** (150 MHz, DMSO-d_6) (**B**)

Figure S8 ^{1}H NMR spectrum (600 MHz, DMSO-d_6) (**A**) and ^{13}C NMR spectrum of **1d** (150 MHz, DMSO-d_6) (**B**)

Figure S9 HMBC spectrum of **1d**

Figure S10 ^{1}H NMR spectrum (600 MHz, DMSO-d_6) (**A**) and ^{13}C NMR spectrum of **1e** (150 MHz, DMSO-d_6) (**B**)

Figure S11 HMBC spectrum of **1e**

Figure S12 ^{1}H NMR spectrum (600 MHz, DMSO-d_6) (**A**) and ^{13}C NMR spectrum of **1f** (150 MHz, DMSO-d_6) (**B**)

Figure S13 HMBC spectrum of **1f**

Figure S14 ^{1}H NMR spectrum (600 MHz, DMSO-d_6) (**A**) and ^{13}C NMR spectrum of **1g** (150 MHz, DMSO-d_6) (**B**)

Figure S15 ^{1}H NMR spectrum (600 MHz, DMSO-d_6) (**A**) and ^{13}C NMR spectrum of **1h** (150 MHz, DMSO-d_6) (**B**)

Figure S16 ^{1}H NMR spectrum (600 MHz, DMSO-d_6) (**A**) and ^{13}C NMR spectrum of **1i** (150 MHz, DMSO-d_6) (**B**)

Figure S17 HMBC spectrum of **1i**

Figure S18 ^{1}H NMR spectrum (600 MHz, DMSO-d_6) (**A**) and ^{13}C NMR spectrum of **1j** (150 MHz, DMSO-d_6) (**B**)

Figure S19 HMBC spectrum of **1j**

Figure S20 ^{1}H NMR spectrum (600 MHz, DMSO-d_6) (**A**) and ^{13}C NMR spectrum of **1k** (150 MHz, DMSO-d_6) (**B**)

Table S1

Position	**1a**	**1b**	**1c**	**1d**	**1e**	**1f**	**1g**
3	6.81, s	6.82, s	6.75, s	6.90, s	6.89, s	6.90, s	7.01, s
5-OH	12.95, s	12.90, s	12.97, s	12.88, s	12.94, s	12.89, s	
6	6.19, d(2.0)	6.20, d(2.0)	6.44, d(2.1)	6.21, d(1.9)	6.43, d(2.0)	6.45, d(2.1)	6.46, d(2.0)
8	6.53, d(2.0)	6.50, d(2.0)	6.79, d(2.2)	6.55, d(1.9)	6.89, d(2.1)	6.86, d(2.1)	6.90, d(2.3)
2'	7.79, d(2.2)	7.50, d(2.2)	7.42, d(2.2)	7.77, d(1.9)	7.85, d(2.0)	7.53, d(2.2)	7.84, d(2.2)
5'	6.98, d(8.5)	7.25, d(8.6)	6.91, d(8.3)	7.29, d(8.8)	6.99, d(8.4)	7.26, d(8.6)	7.31, d(8.6)
6'	7.66,dd(8.5,2.1)	7.52,dd(8.5,2.3)	7.45,dd(8.3,2.2)	7.71,dd(8.6,1.9)	7.69,dd(8.5,2.0)	7.56,dd(8.5,2.2)	7.77,dd(8.3,2.2)
	Glc	Glc	Glc	Glc	Glc	Glc	Glc
1''	4.91, d(7.3)	4.89, d(7.3)	5.07, d(7.6)	5.29, d(4.7)	5.06, d(7.4)	5.08, d(7.6)	4.9-5.2
1'''				5.26, d(4.3)	4.88, d(7.3)	4.89, d(7.4)	4.9-5.2
1''''							4.9-5.2
H of sugar	3.1-3.8	3.1-3.8	3.1-3.8	3.1-3.8	3.1-3.8	3.1-3.8	3.1-3.8

Table S2

Position	**1a**	**1b**	**1c**	**1d**	**1e**	**1f**	**1g**
2	163.9,C	163.2,C	164.6,C	163.0,C	164.0,C	163.8, C	163.4,C
3	103.7,CH	103.8,CH	103.2,CH	104.3,CH	103.9,CH	104.3,CH	104.4,CH
4	182.2,C	181.8,C	182.0,C	181.9,C	182.1,C	182.1,C	182.1,C
5	161.8,C	161.4,C	161.2,C	161.5,C	161.1,C	161.1,C	161.1,C
6	99.3,CH	98.9,CH	99.6,CH	99.0,CH	99.6,CH	99.3,CH	99.5,CH
7	164.6,C	164.3,C	163.0,C	164.4,C	163.0,C	163.1,C	163.0,C
8	94.6,CH	94.0,CH	94.8,CH	94.3,CH	95.0,CH	94.9,CH	95.0,CH
9	157.8,C	157.3,C	157.0,C	157.5,C	157.1,C	157.1,C	157.0,C
10	104.2,C	104.0,C	105.4,C	103.9,C	105.4,C	105.5,C	105.4,C
1'	122.2,C	124.7,C	121.4,C	124.5,C	121.6,C	124.6,C	124.6,C
2'	114.8,CH	113.6,CH	113.6,CH	115.1,CH	114.5,CH	113.8,CH	115.0,CH
3'	146.1,C	146.9,C	145.9,C	147.1,C	145.8,C	147.0,C	147.1,C
4'	151.0,C	148.5,C	150.0,C	150.2,C	150.8,C	148.8,C	150.3,C
5’	116.9,CH	116.0,CH	116.1,CH	116.9,CH	116.6,CH	116.0,CH	116.8,CH
6'	122.4,CH	118.5,CH	119.3,CH	121.4,CH	122.2,CH	118.8,CH	121.4,CH
	Glc	Glc	Glc	Glc	Glc	Glc	
1''	102.3,CH	101.2,CH	100.0,CH	101.0,CH	99.8,CH	99.9,CH	99.8,CH
2''	73.8,CH	72.5,CH	73.2,CH	73.4,CH	73.2,CH	73.3,CH	73.3,CH
3''	76.4,CH	75.9,CH	76.4,CH	76.6,CH	76.5,CH	76.5,CH	76.6,CH
4''	70.5,CH	69.8,CH	69.6,CH	69.9,CH	69.4,CH	69.9,CH	70.0,CH
5''	77.8,CH	77.3,CH	77.2,CH	77.2,CH	77.1,CH	77.4,CH	77.2,CH
6''	61.3,CH_2	60.7,CH_2	60.7,CH_2	60.9,CH_2	60.5,CH_2	60.8,CH_2	61.0,CH_2
				Glc	Glc	Glc	
1'''				100.4,CH	102.0,CH	101.2,CH	101.0,CH
2'''				73.3,CH	73.4,CH	73.2,CH	73.2,CH
3'''				76.5,CH	76.0,CH	75.9,CH	76.5,CH
4'''				69.7,CH	70.3,CH	69.6,CH	69.6,CH
5'''				77.2,CH	77.6,CH	77.2,CH	77.1,CH
6'''				60.7,CH_2	61.2,CH_2	60.7,CH_2	60.6,CH_2
							Glc
1''''							100.3,CH
2''''							73.1,CH
3''''							76.4,CH
4''''							69.4,CH
5''''							77.0,CH
6''''							60.5,CH_2

Table S3

Position	**1k**	**1j**	**1i**	**1h**
3	6.89, s	6.75, s	6.79, s	6.85, s
5-OH	12.89, s	12.97, s	12.89, s	12.94, s
6	6.43, d(2.2)	6.41, d(2.1)	6.19, d(1.7)	6.20, d(2.0)
8	6.85, d(2.2)	6.79, d(2.2)	6.48, d(1.6)	6.49, d(2.0)
2'	7.56, overlapped	7.44, d(2.3)	7.48, d(2.0)	7.65, overlapped
5'	7.19, d(8.9)	6.91, d(8.4)	7.17, d(8.5)	6.99, d(9.0)
6'	7.56, overlapped	7.47, dd(8.3,2.3)	7.51, dd(8.8,1.9)	7.65, overlapped
	Xyl	Xyl	Xyl	Xyl
1''	4.91, d(7.5)	5.09, d(7.3)	4.90, d(7.5)	5.02, d(7.4)
1'''	5.10, m			
H of sugar	3.1-3.8	3.1-3.5	3.1-3.5	3.1-3.5

Table S4

Position	**1k**	**1j**	**1i**	**1h**
2	163.8,C	164.5,C	163.2,C	163.3,C
3	104.3,CH	103.1,CH	103.8,CH	103.3,CH
4	182.1,C	181.9,C	181.8,C	181.8,C
5	161.1,C	161.1,C	161.5,C	161.5,C
6	99.6,CH	99.5,CH	99.0,CH	98.9,CH
7	162.8,C	162.7,C	164.6,C	164.2,C
8	94.7,CH	94.6,CH	94.1,CH	94.0,CH
9	157.0,C	157.0,C	157.4,C	157.3,C
10	105.5,C	105.4,C	104.1,C	103.7,C
1'	124.7,C	121.4,C	124.9,C	121.6,C
2'	113.9,CH	113.6,CH	113.8,CH	114.4,CH
3'	147.1,C	145.8,C	147.1,C	145.2,C
4'	148.4,C	149.9,C	148.3,C	151.1,C
5’	116.0,CH	116.0,CH	116.1,CH	116.6,CH
6'	118.7,CH	119.2,CH	118.5,CH	122.0,CH
	Xyl	Xyl	Xyl	Xyl
1''	100.2,CH	100.2,CH	101.7,CH	101.8,CH
2''	72.9,CH	72.9,CH	73.1,CH	73.1,CH
3''	76.3,CH	76.3,CH	75.8,CH	75.9,CH
4''	69.2,CH	69.2,CH	69.4,CH	69.4,CH
5''	65.8,CH_2	65.8,CH_2	65.8, CH_2	65.7,CH_2
	Xyl			
1'''	101.7,CH			
2'''	73.0,CH			
3'''	75.7,CH			
4'''	69.4,CH			
5'''	65.8,CH			

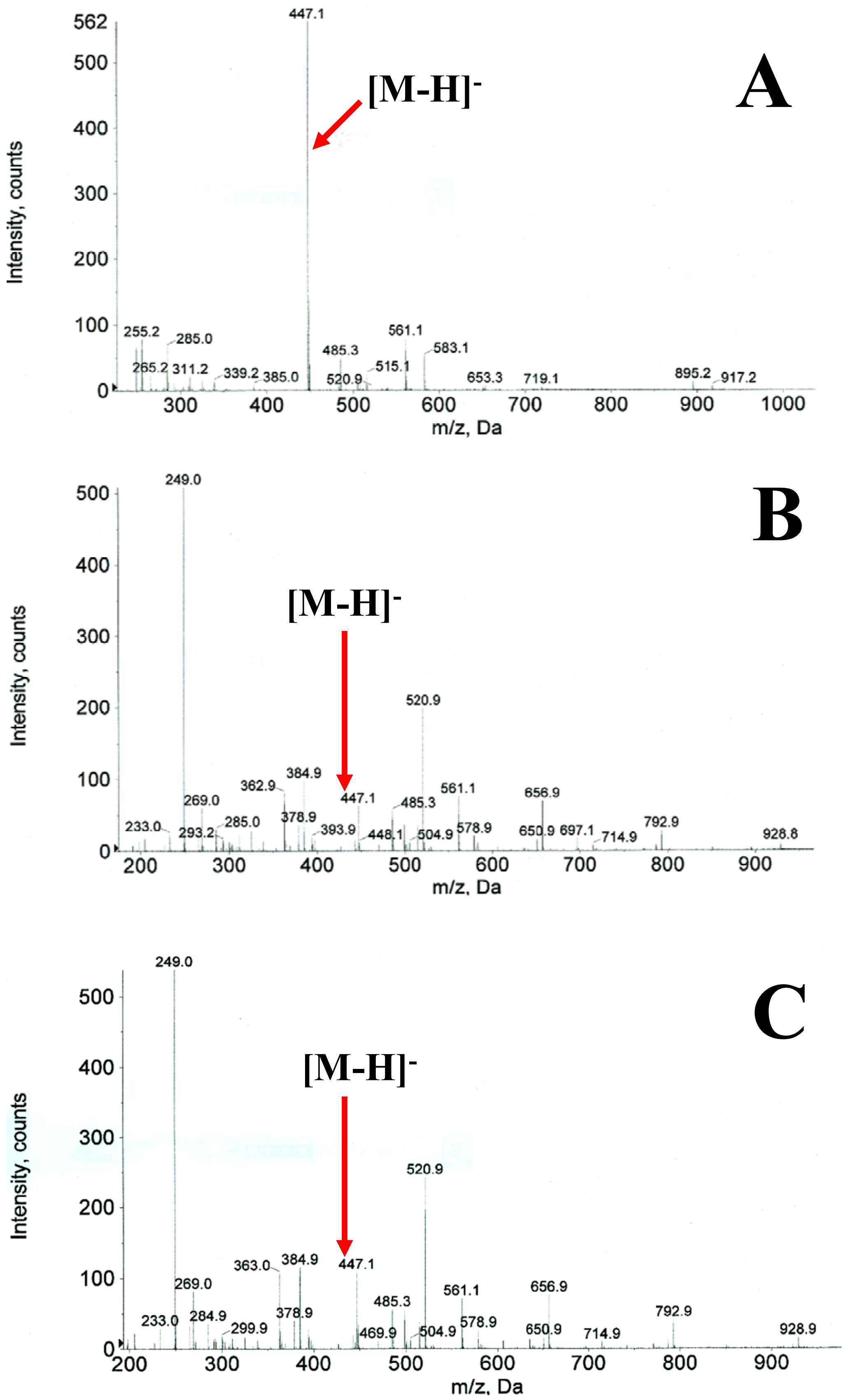
A
B
C
[M-H]-
Intensity, counts
m/z, Da
447.1
255.2
285.0
265.2
311.2
339.2
385.0
485.3
520.9
515.1
561.1
583.1
653.3
719.1
895.2
917.2
249.0
520.9
384.9
362.9
269.0
447.1
485.3
561.1
656.9
378.9
233.0
293.2
285.0
393.9
448.1
504.9
578.9
650.9
697.1
714.9
792.9
928.8
363.0
284.9
299.9
469.9
650.9
928.9

Figure S1

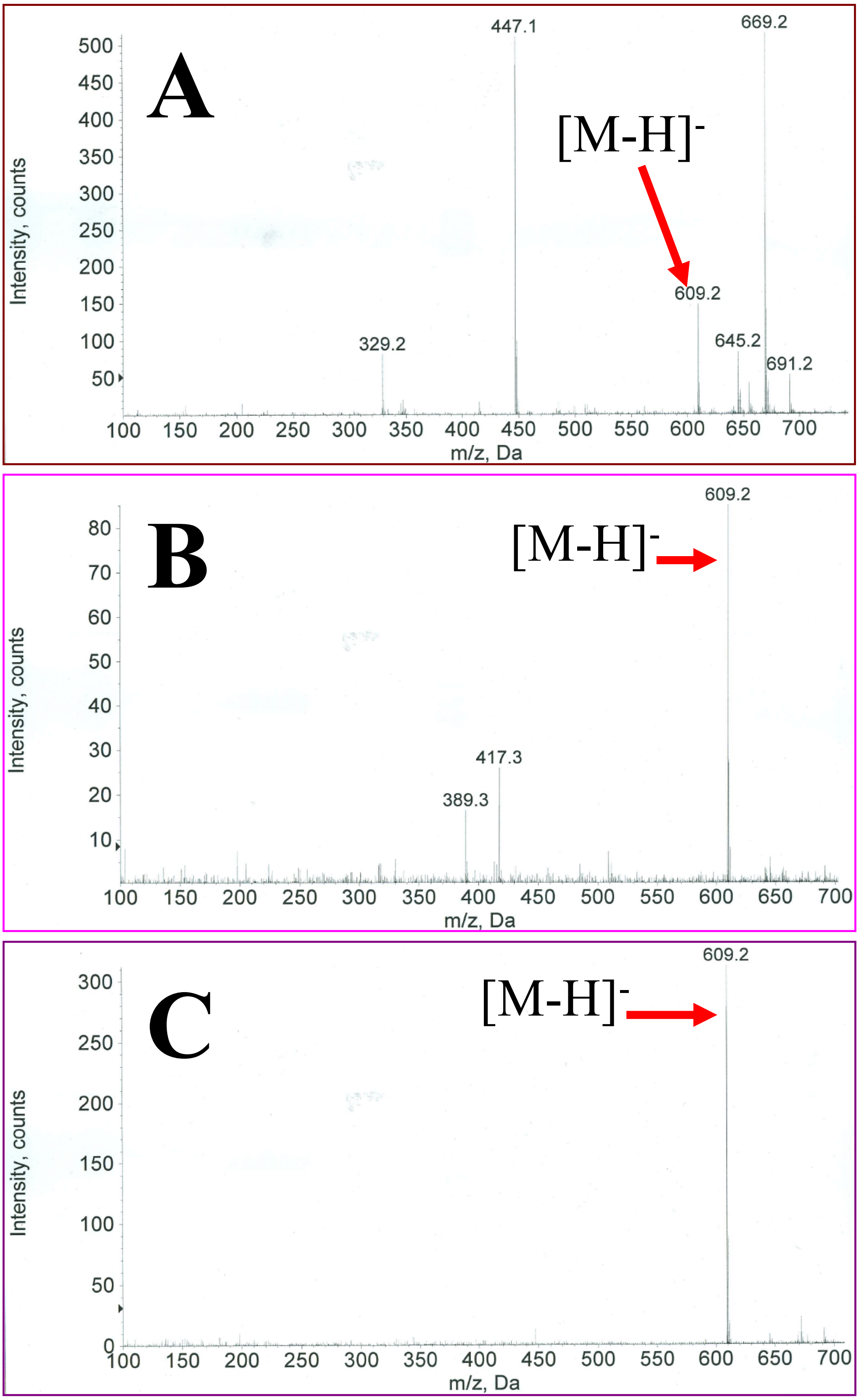
A
B
C
[M-H]-
Intensity, counts
m/z, Da
447.1
669.2
609.2
329.2
645.2
691.2
417.3
389.3

Figure S2

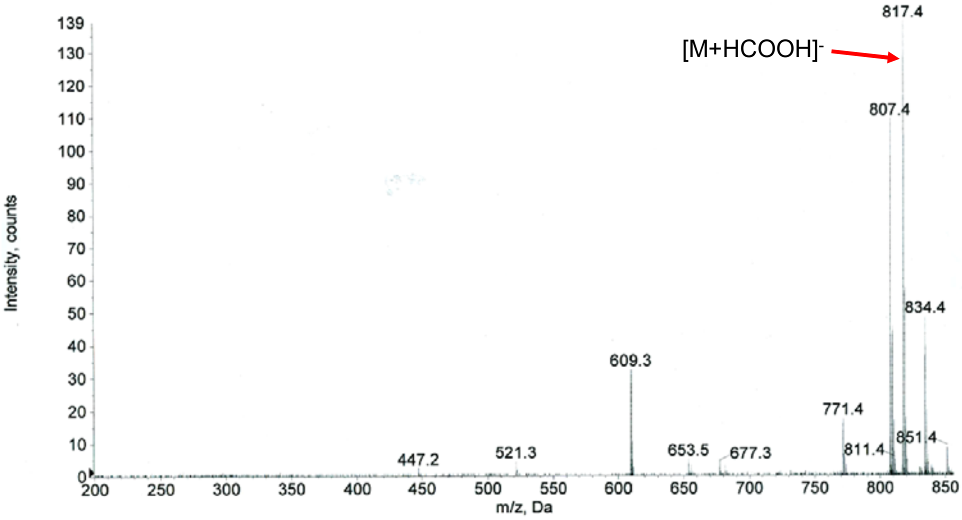

Figure S3

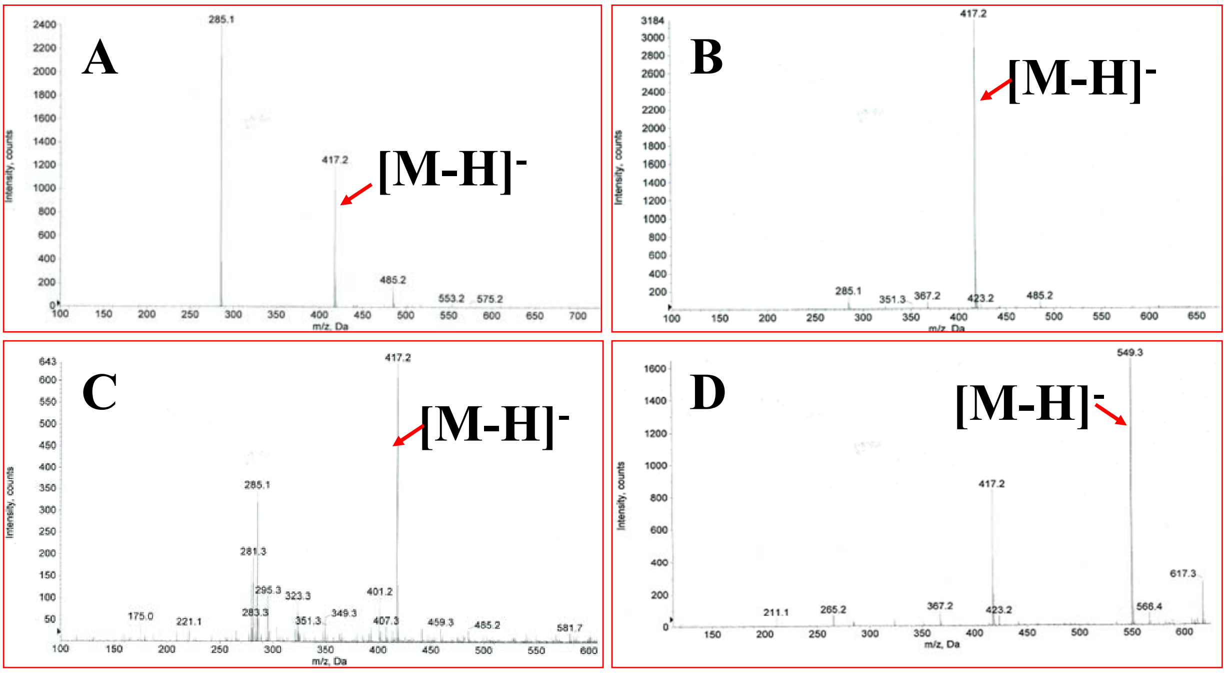

Figure S4

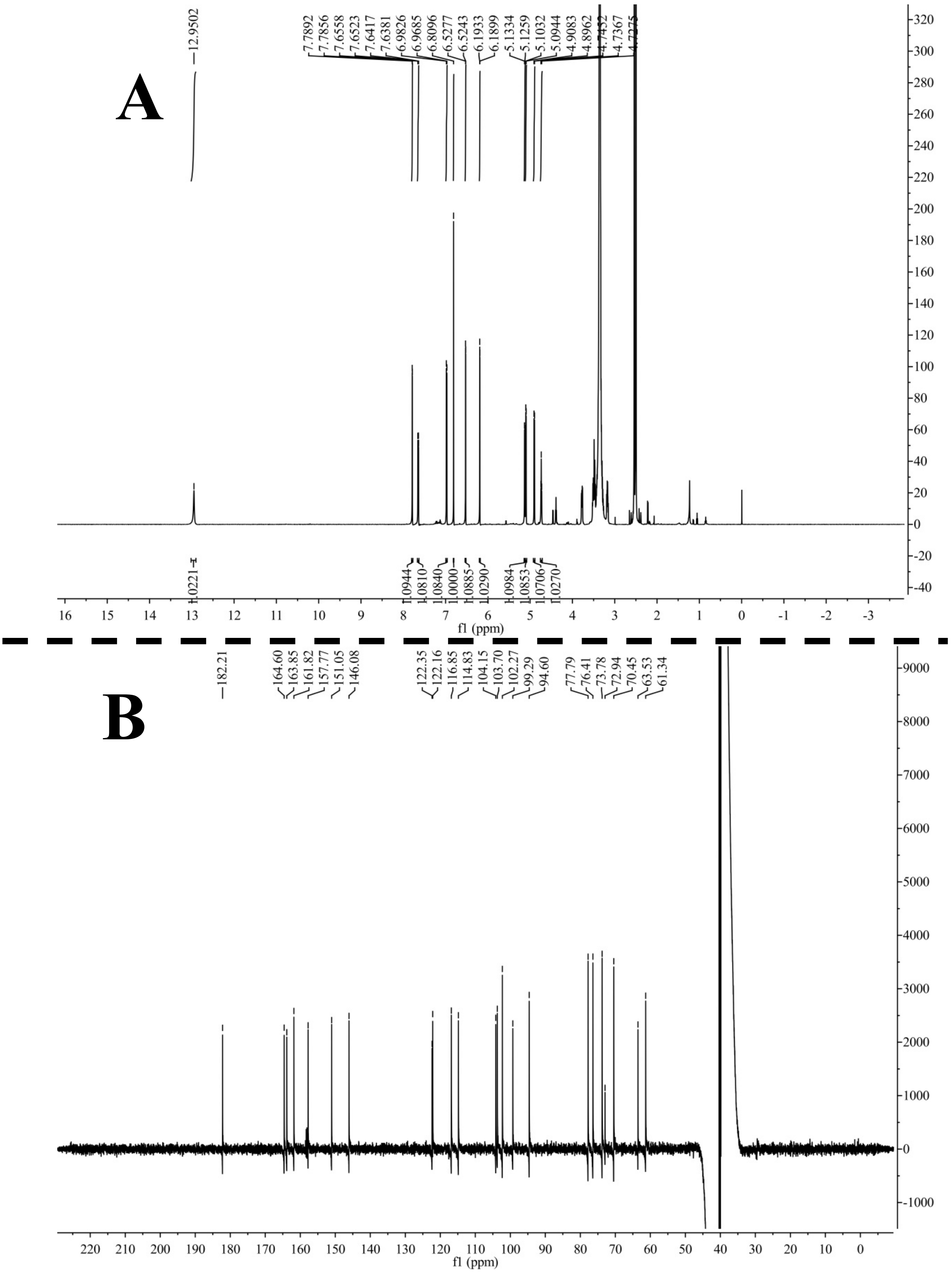
A
12.9502
7.7892
7.7856
7.6558
7.6523
7.6417
7.6381
6.9826
6.9685
6.8096
6.5277
6.5243
6.1933
6.1899
5.1334
5.1259
5.1032
5.0944
4.9083
4.8962
4.7432
4.7367
4.7275
1.0221
1.0944
1.0810
1.0840
1.0000
1.0885
1.0290
1.0984
1.0853
1.0706
1.0270
f1 (ppm)
B
182.21
164.60
163.85
161.82
157.77
151.05
146.08
122.35
122.16
116.85
114.83
104.15
103.70
102.27
99.29
94.60
77.79
76.41
73.78
72.94
70.45
63.53
61.34
f1 (ppm)

Figure S5

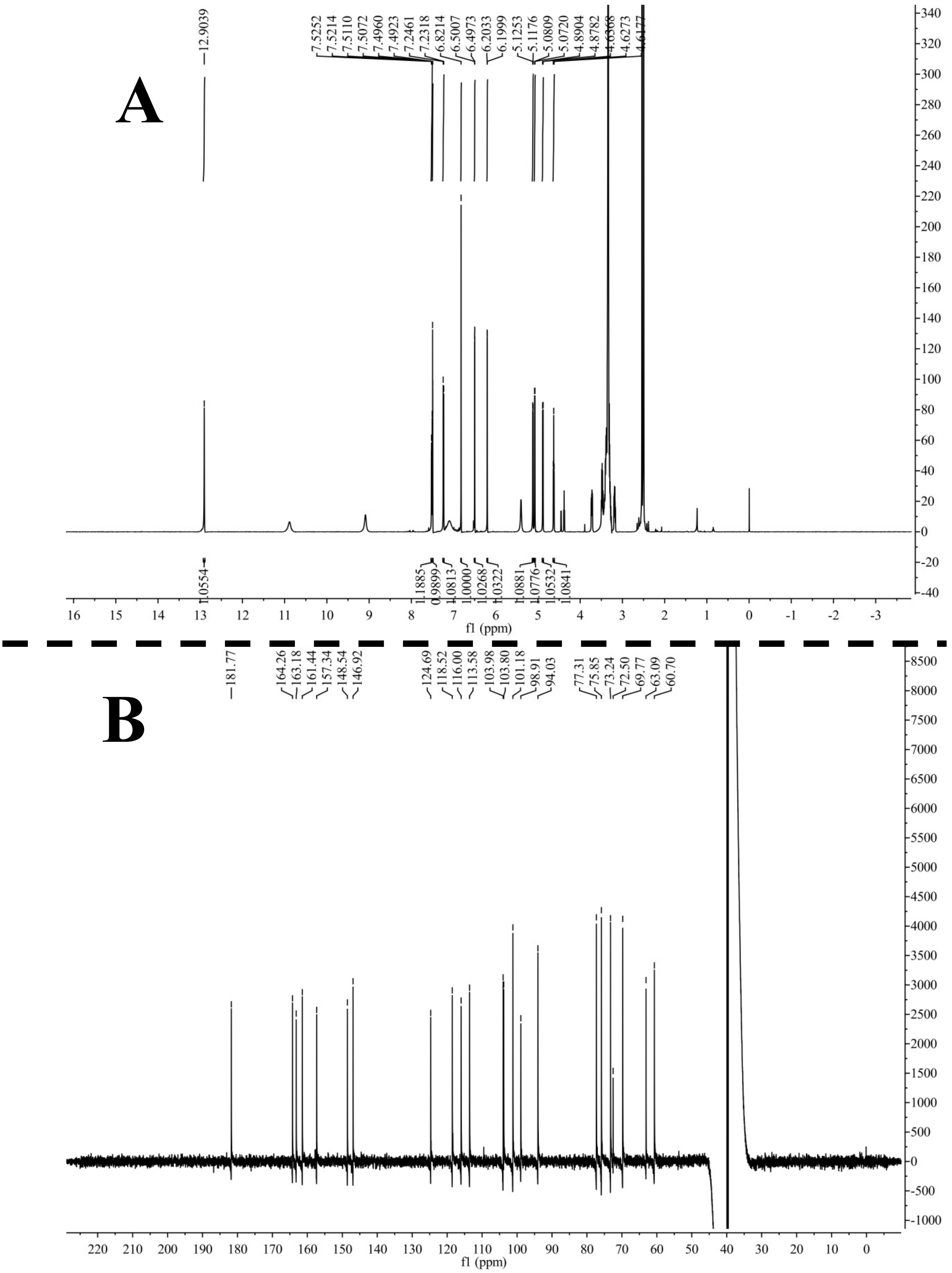
A
12.9039
7.5252
7.5214
7.5110
7.5072
7.4960
7.4923
7.2461
7.2318
6.8214
6.5007
6.4973
6.2033
6.1999
5.1253
5.1176
5.0809
5.0720
4.8904
4.8782
4.6368
4.6273
4.6177
1.0554
1.1885
0.9899
1.0813
1.0000
1.0268
1.0322
1.0881
1.0776
1.0532
1.0841
f1 (ppm)
B
181.77
164.26
163.18
161.44
157.34
148.54
146.92
124.69
118.52
116.00
113.58
103.98
103.80
101.18
98.91
94.03
77.31
75.85
73.24
72.50
69.77
63.09
60.70
f1 (ppm)

Figure S6

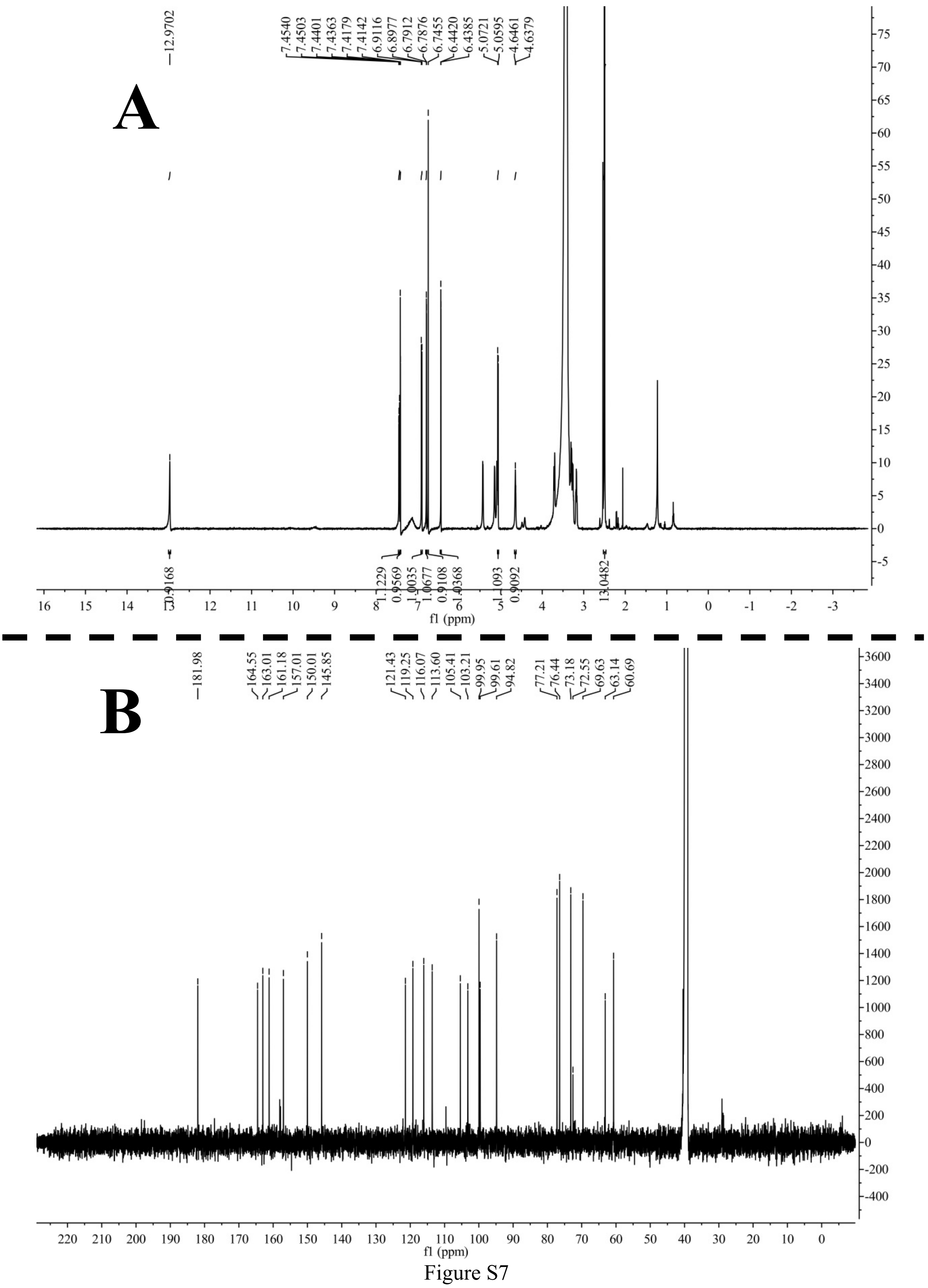
A
B
12.9702
7.4540
7.4503
7.4401
7.4363
7.4179
7.4142
6.9116
6.8977
6.7912
6.7876
6.7455
6.4420
6.4385
5.0721
5.0595
4.6461
4.6379
0.9168
1.1229
0.9569
1.0035
1.0677
0.9108
1.0368
1.1093
0.9092
13.0482
f1 (ppm)
181.98
164.55
163.01
161.18
157.01
150.01
145.85
121.43
119.25
116.07
113.60
105.41
103.21
99.95
99.61
94.82
77.21
76.44
73.18
72.55
69.63
63.14
60.69
f1 (ppm)

Figure S7

A

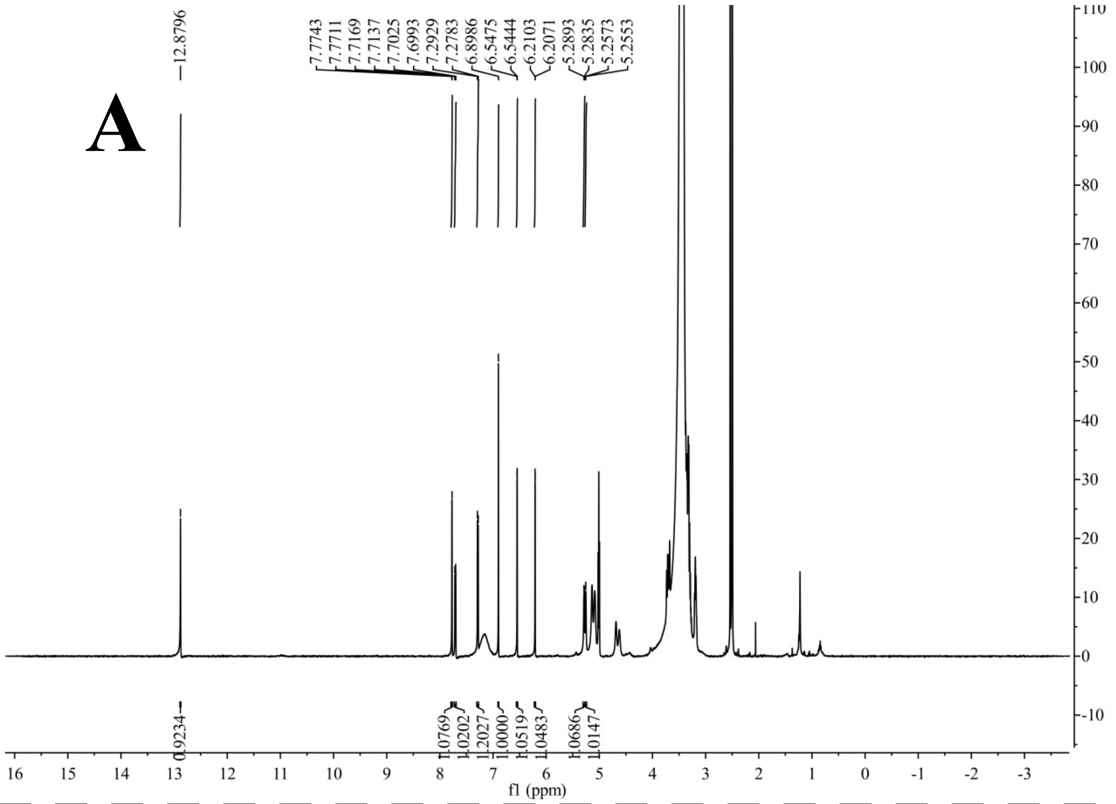

12.8796
7.7743
7.7711
7.7169
7.7137
7.7025
7.6993
7.2929
7.2783
6.8986
6.5475
6.5444
6.2103
6.2071
5.2893
5.2835
5.2573
5.2553
0.9234
1.0769
1.0202
1.2027
1.0000
1.0519
1.0483
1.0686
1.0147
f1 (ppm)

B

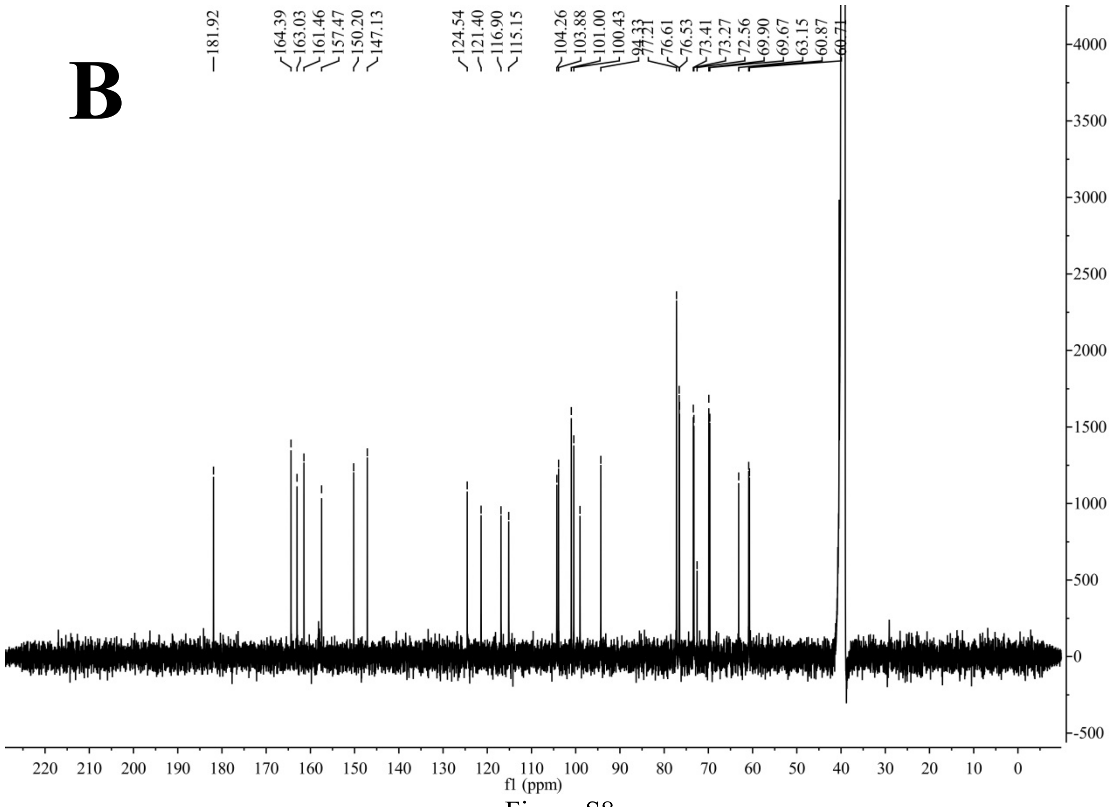

181.92
164.39
163.03
161.46
157.47
150.20
147.13
124.54
121.40
116.90
115.15
104.26
103.88
101.00
100.43
94.33
77.21
76.61
76.53
73.41
73.27
72.56
69.90
69.67
63.15
60.87
60.71
f1 (ppm)

Figure S8

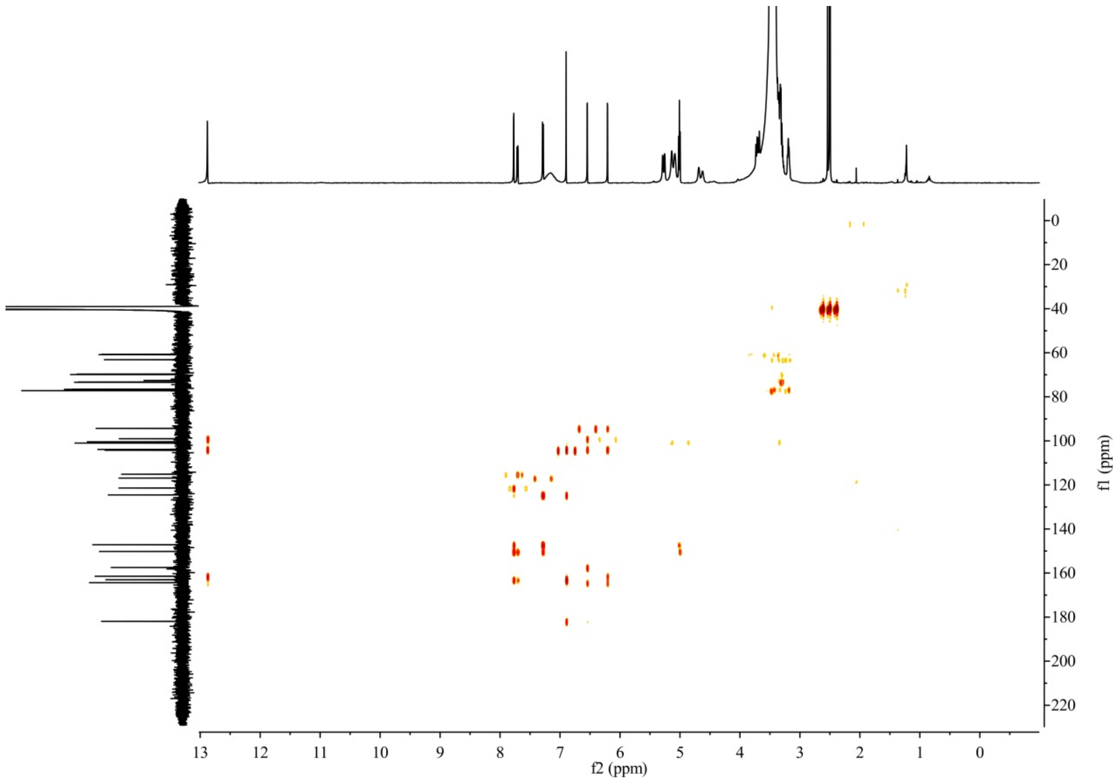

Figure S9

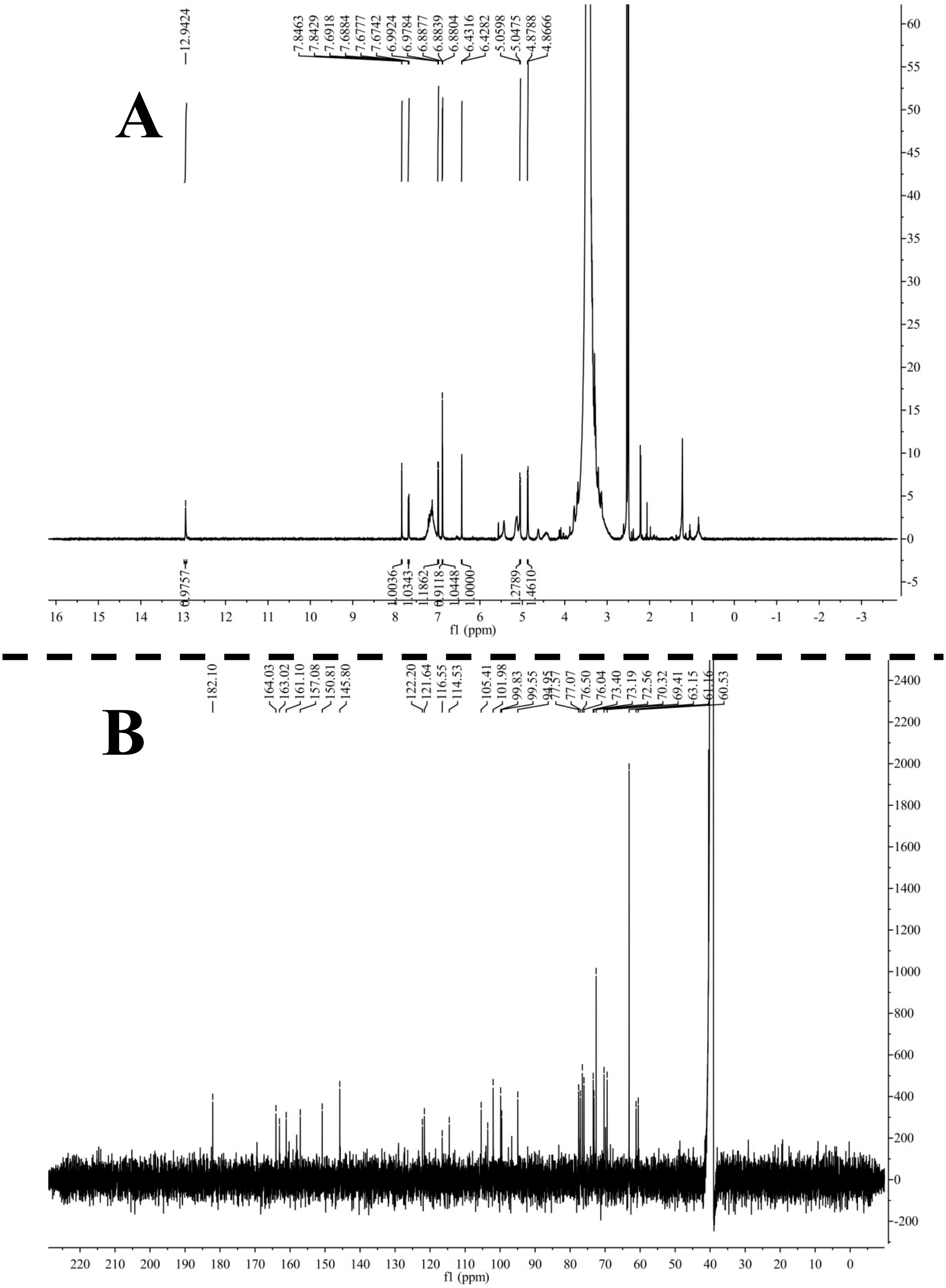

Figure S10

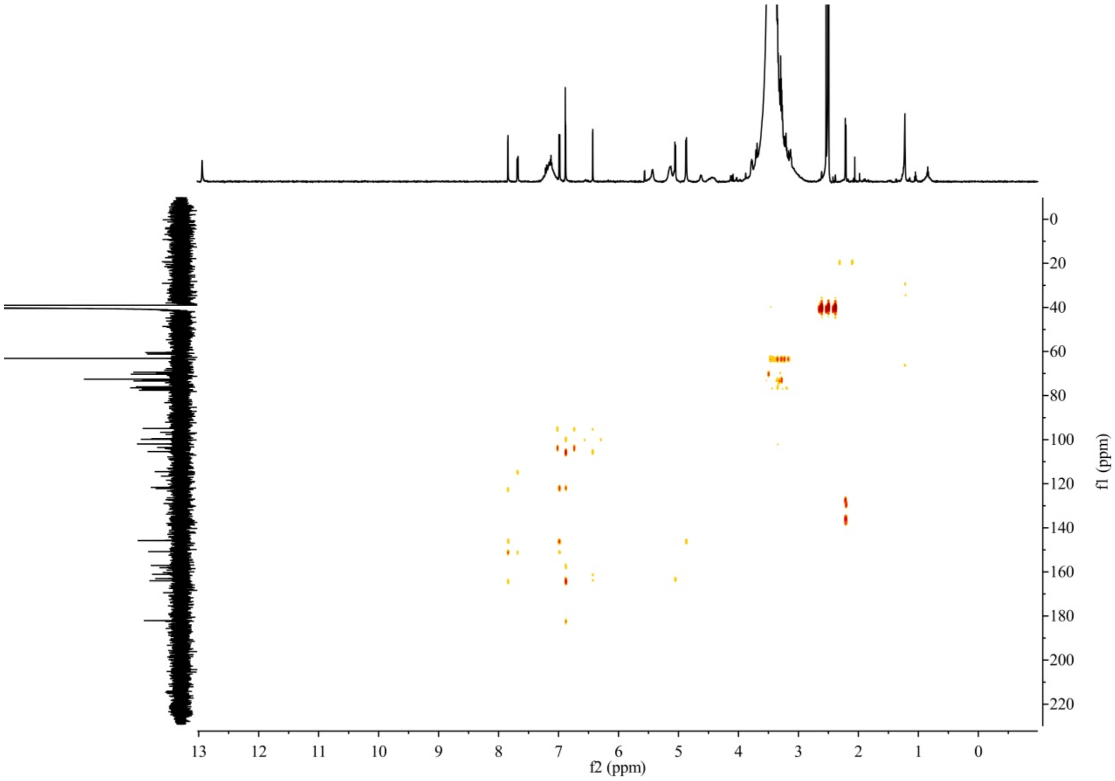

Figure S11

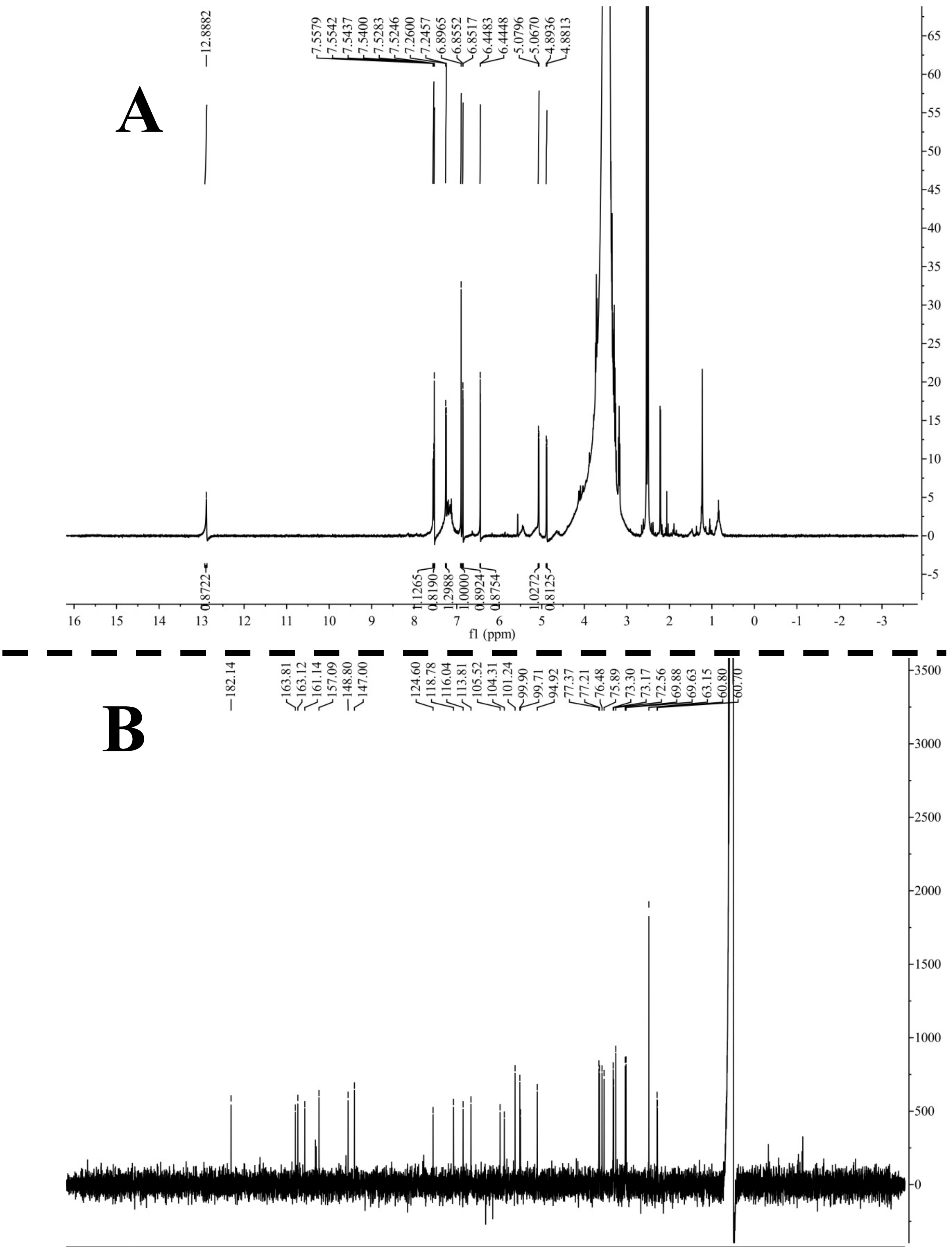
A
12.8882
7.5579
7.5542
7.5437
7.5400
7.5283
7.5246
7.2600
7.2457
6.8965
6.8552
6.8517
6.4483
6.4448
5.0796
5.0670
4.8936
4.8813
0.8722
1.1265
0.8190
1.2988
1.0000
0.8924
0.8754
1.0272
0.8125
f1 (ppm)
B
182.14
163.81
163.12
161.14
157.09
148.80
147.00
124.60
118.78
116.04
113.81
105.52
104.31
101.24
99.90
99.71
94.92
77.37
77.21
76.48
75.89
73.30
73.17
72.56
69.88
69.63
63.15
60.80
60.70
f1 (ppm)

Figure S12

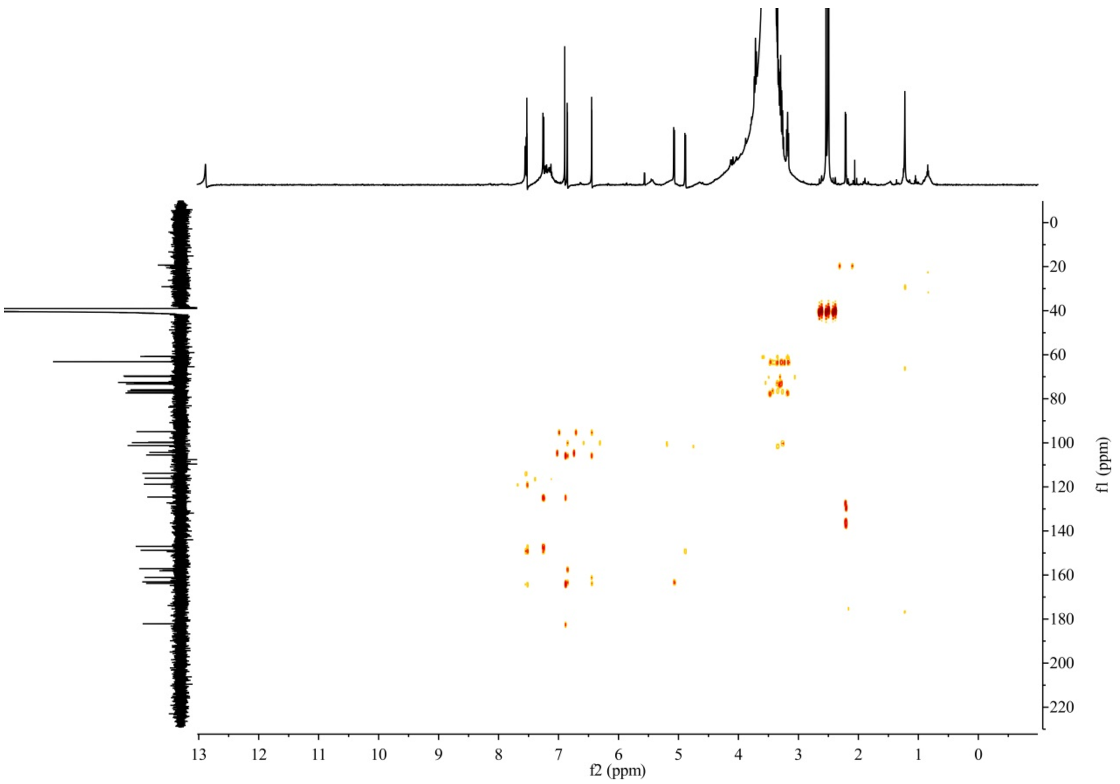

Figure S13

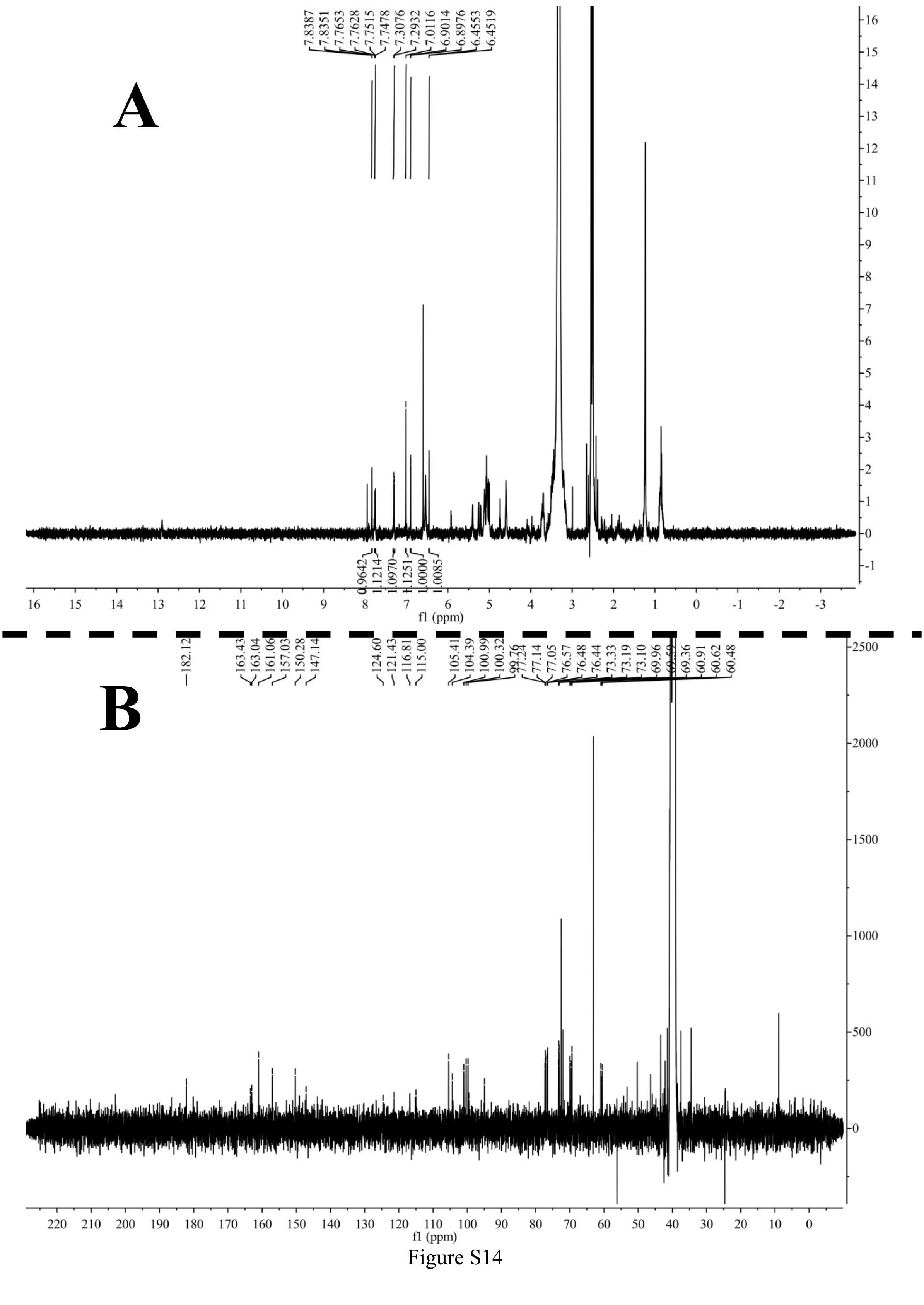

Figure S14

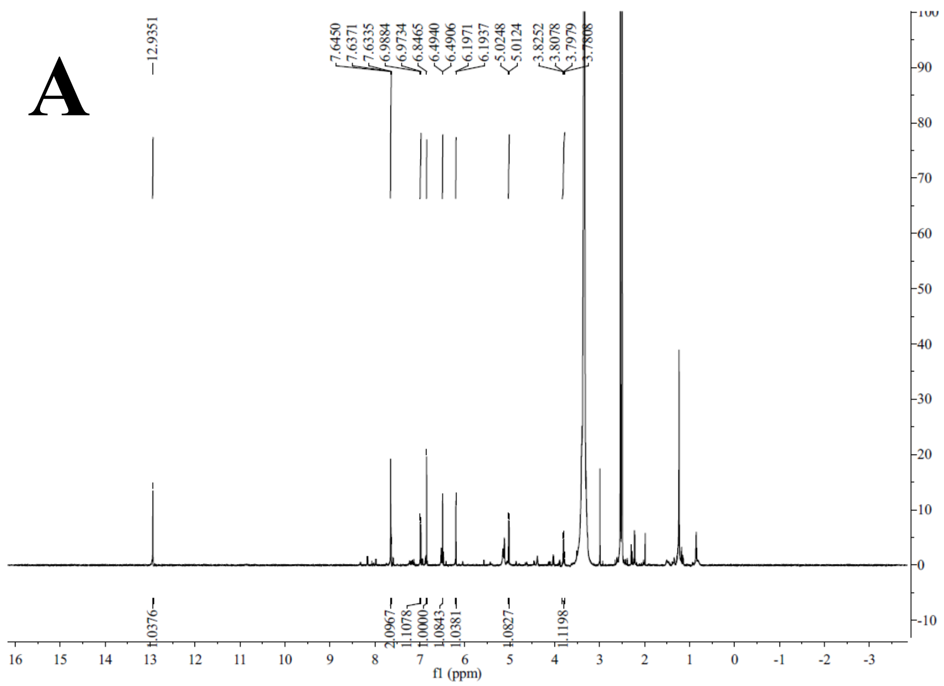
A
12.9351
7.6450
7.6371
7.6335
6.9884
6.9734
6.8465
6.4940
6.4906
6.1971
6.1937
5.0248
5.0124
3.8252
3.8078
3.7979
3.7808
1.0376
2.0967
1.1078
1.0000
1.0843
1.0381
1.0827
1.1198
f1 (ppm)

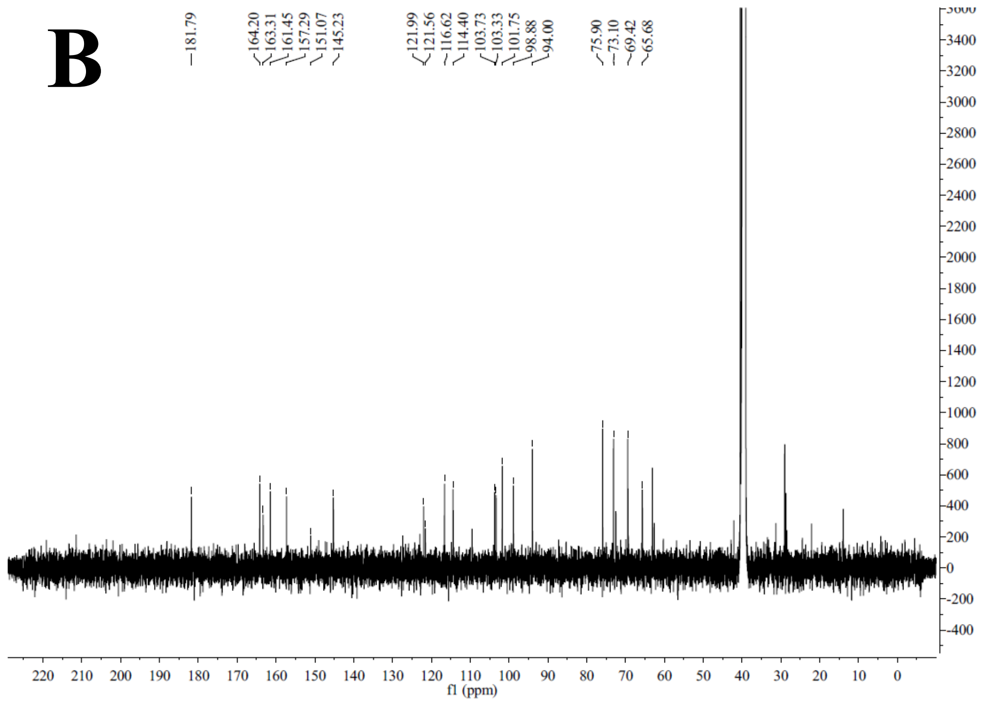
B
181.79
164.20
163.31
161.45
157.29
151.07
145.23
121.99
121.56
116.62
114.40
103.73
103.33
101.75
98.88
94.00
75.90
73.10
69.42
65.68
f1 (ppm)

Figure S15

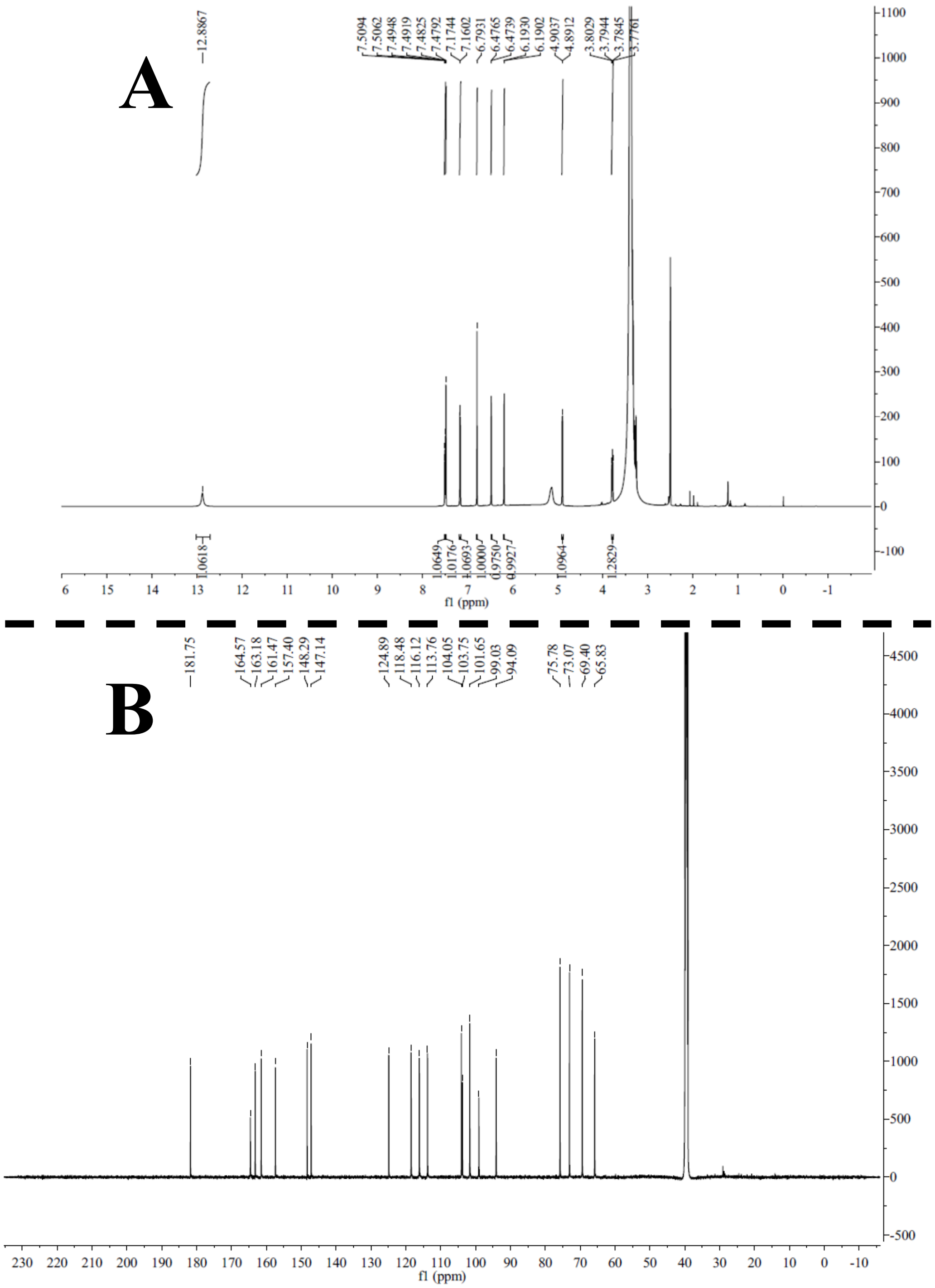
A
12.8867
7.5094
7.5062
7.4948
7.4919
7.4825
7.4792
7.1744
7.1602
6.7931
6.4765
6.4739
6.1930
6.1902
4.9037
4.8912
3.8029
3.7944
3.7845
3.7761
1.0618
1.0649
1.0176
1.0693
1.0000
0.9750
0.9927
1.0964
1.2829
f1 (ppm)
B
181.75
164.57
163.18
161.47
157.40
148.29
147.14
124.89
118.48
116.12
113.76
104.05
103.75
101.65
99.03
94.09
75.78
73.07
69.40
65.83
f1 (ppm)

Figure S16

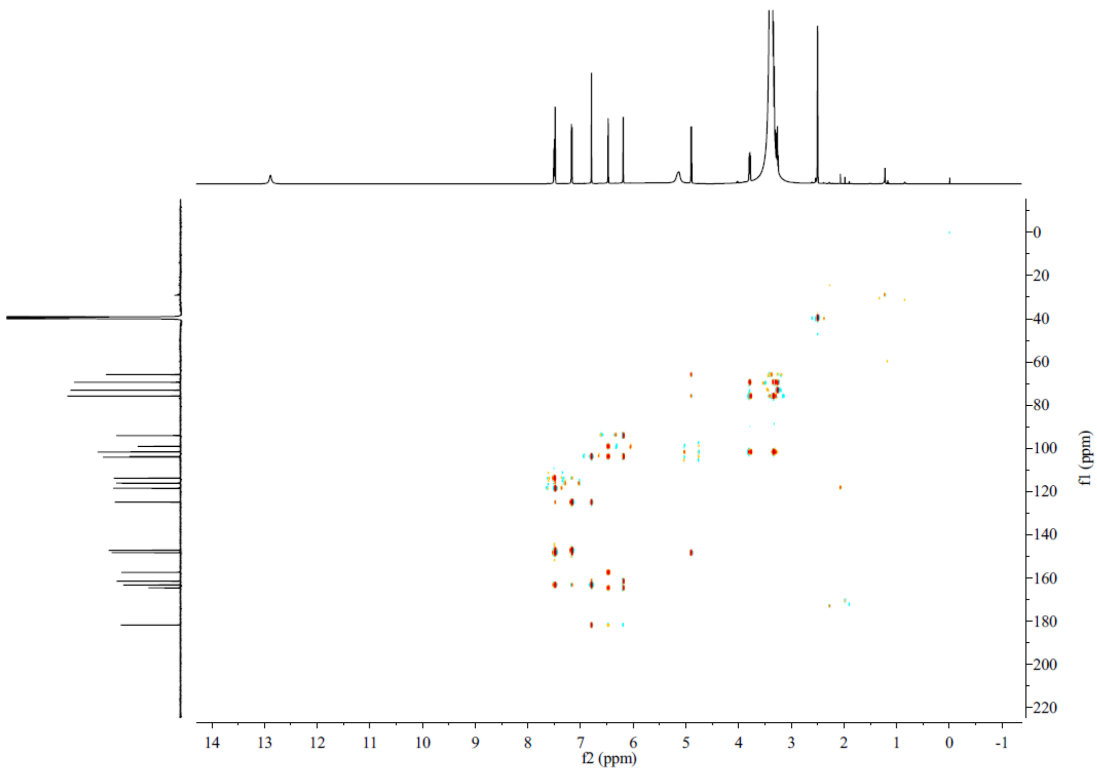

14
13
12
11
10
9
8
7
6
5
4
3
2
1
0
-1
f2 (ppm)
0
20
40
60
80
100
120
140
160
180
200
220
f1 (ppm)

Figure S17

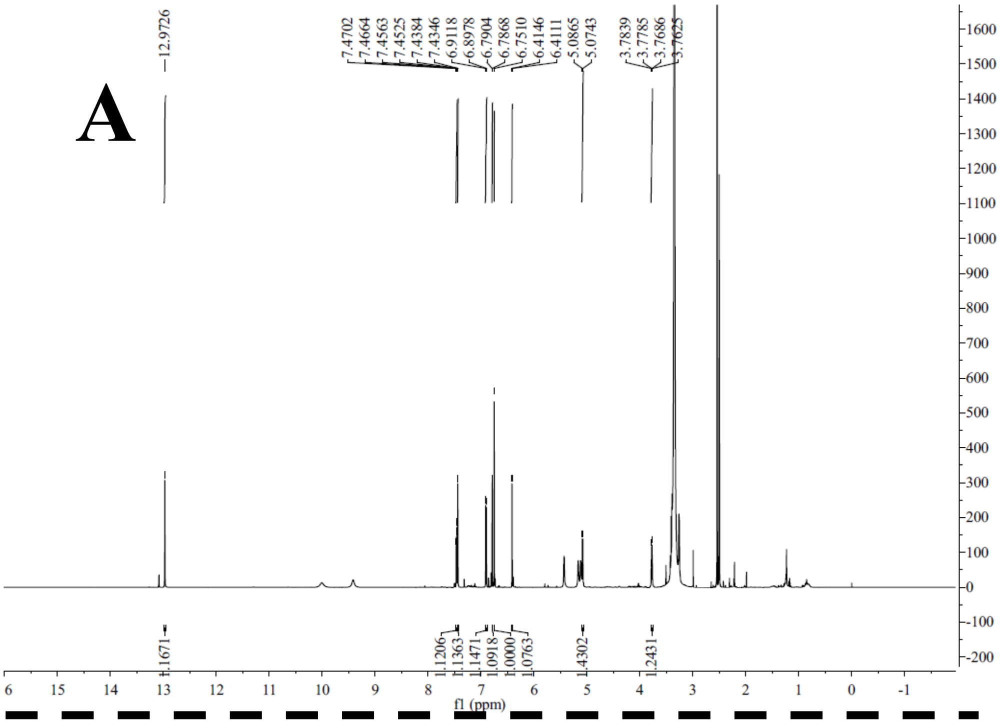
A
12.9726
7.4702
7.4664
7.4563
7.4525
7.4384
7.4346
6.9118
6.8978
6.7904
6.7868
6.7510
6.4146
6.4111
5.0865
5.0743
3.7839
3.7785
3.7686
3.7625
1.1671
1.1206
1.1363
1.1471
1.0918
1.0000
1.0763
1.4302
1.2431
f1 (ppm)

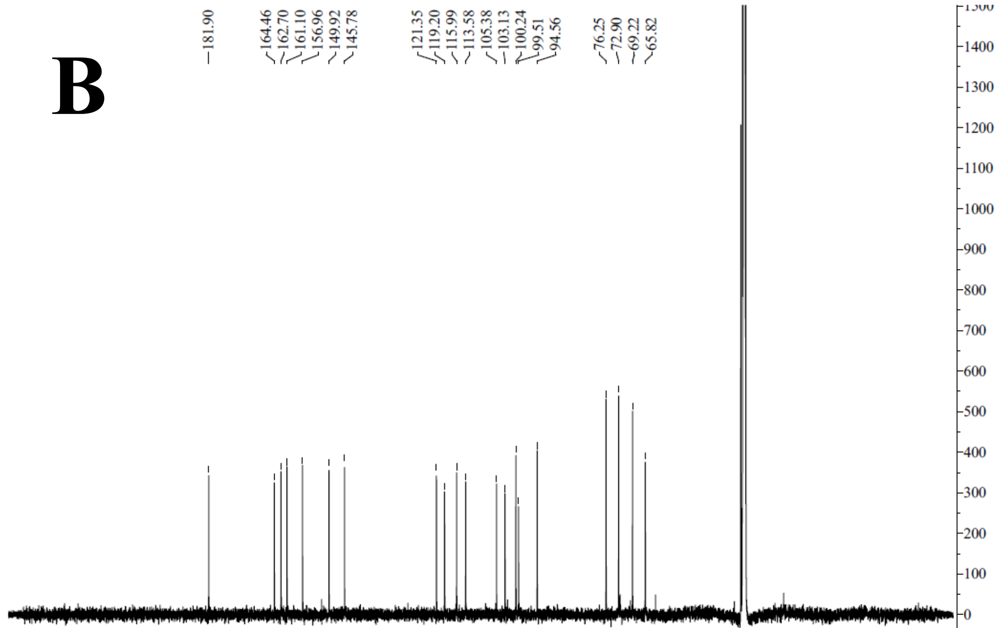
B
181.90
164.46
162.70
161.10
156.96
149.92
145.78
121.35
119.20
115.99
113.58
105.38
103.13
100.24
99.51
94.56
76.25
72.90
69.22
65.82
f1 (ppm)

Figure S18

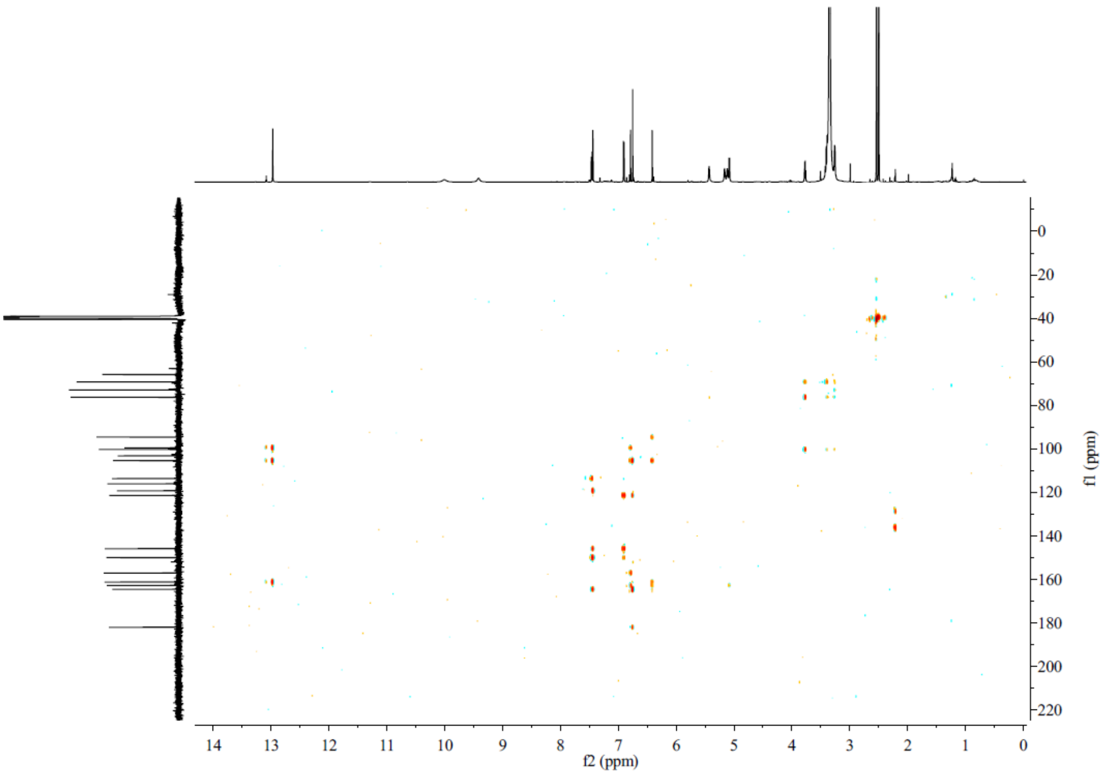

Figure S19

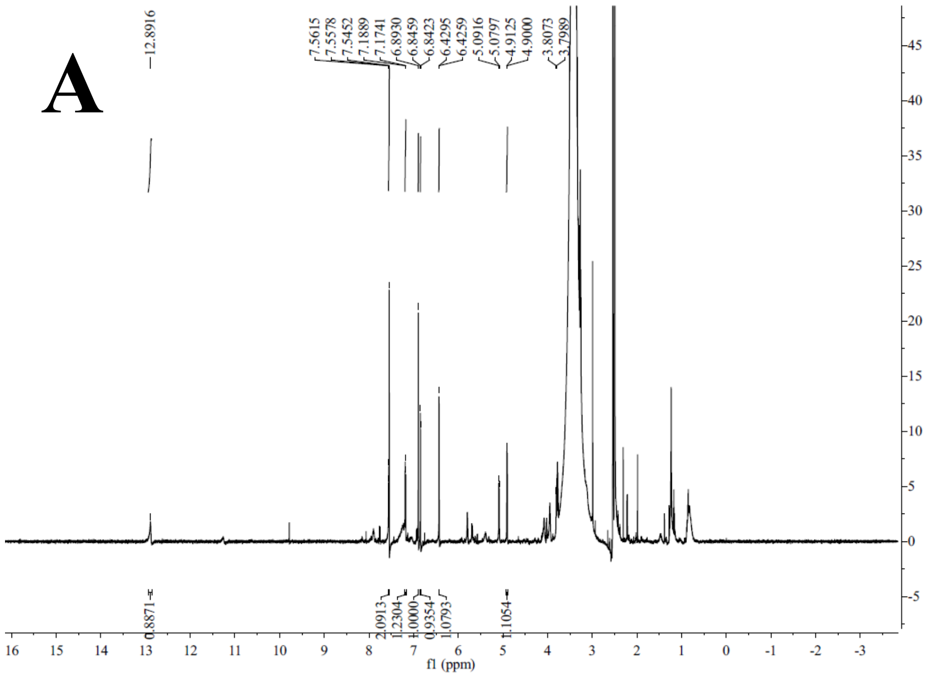
A
12.8916
7.5615
7.5578
7.5452
7.1889
7.1741
6.8930
6.8459
6.8423
6.4295
6.4259
5.0916
5.0797
4.9125
4.9000
3.8073
3.7989
0.8871
2.0913
1.2304
1.0000
0.9354
1.0793
1.1054
f1 (ppm)

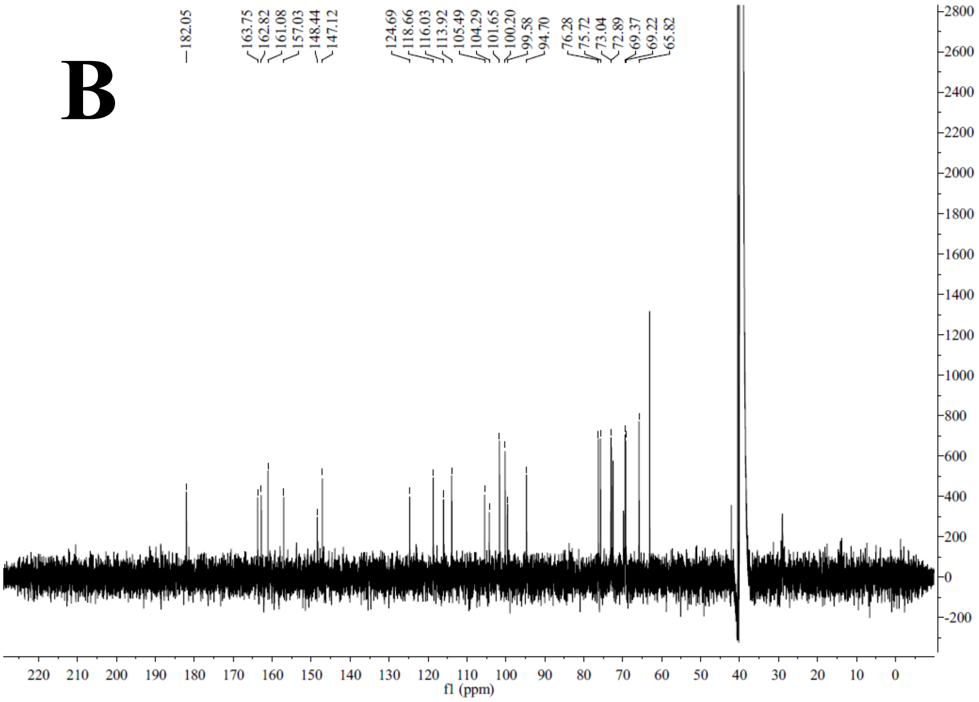
B
182.05
163.75
162.82
161.08
157.03
148.44
147.12
124.69
118.66
116.03
113.92
105.49
104.29
101.65
100.20
99.58
94.70
76.28
75.72
73.04
72.89
69.37
69.22
65.82
f1 (ppm)

Figure S20

Supplementary result section III

Section III is a database presenting HPLC chromatogram (Figure S1), UV spectrum (Figure S1), mass spectrum (Figure S2) and NMR spectrum (Figures S3-4 and Tables S1-2) for compounds **6a** and **6b**.

Table S1 ^{1}H NMR (600 MHz) spectroscopic data (δ in ppm, J in Hz) for compounds **6a** and **6b** in DMSO-d_6

Table S2 ^{13}C NMR (150 MHz) spectroscopic data (δ in ppm, J in Hz) for compounds **6a** and **6b** in DMSO-d_6

Figure S1 HPLC chromatogram of glycosylated metabolites (**6a** and **6b**) of genistein (**6**) with OcUGT1 (a) or without OcUGT1 (b). The UV absorption spectrum of **6a** or **6b** is similar to that of **6**. All of them are marked in the top panels.

Figure S2 The mass spectra of **6a(A)** and **6b(B)** analyzed on ESI-TOF MS.

Figure S3 ^{1}H-NMR spectrum (600 MHz, DMSO-d_6) (**A**) and ^{13}C-NMR spectrum of **6a** (150 MHz, DMSO-d_6) (**B**)

Figure S4 ^{1}H NMR spectrum (600 MHz, DMSO-d_6) (**A**) and ^{13}C NMR spectrum of **6b** (150 MHz, DMSO-d_6) (**B**)

Table S1

Position	**6a**	**6b**
5-OH	12.89, 1H, s	12.93, 1H, s
4'-OH		9.64, 1H, brs
2	8.36, 1H, s	8.42, 1H, s
2',6'	7.49, 2H, m	7.40, 2H, m
3',5'	7.10, 2H, m	6.83, 2H, m
8	6.40, 1H, d(2.2Hz)	6.72, 1H, d(2.2Hz)
6	6.23, 1H, d(2.1Hz)	6.47, 1H, d(2.2Hz)
	Glc	Glc
1''	5.01,1H,m	5.28,1H,d(7.9Hz)
H of sugar	3.00-3.70	2.90-3.60

Table S2

Position	**6a**	**6b**
2	154.9, C	154.6, C
3	124.6, C	122.6, C
4	180.5, C	180.6, C
5	162.4, C	161.7, C
6	100.8, CH	99.6, CH
7	164.8, C	163.0, C
8	94.2, CH	94.6, CH
9	158.1, C	157.3, C
10	104.9, C	106.1, C
1'	122.4, C	121.1, C
2'	130.5, CH	130.2, CH
3'	115.2, CH	115.2, CH
4'	157.7, C	157.5, C
5'	116.5, CH	115.2, CH
6'	130.5, CH	130.2, CH
	Glc	Glc
1''	99.5, CH	99.9, CH
2''	77.0, CH	73.1, CH
3''	73.7, CH	76.4, CH
4''	77.5, CH	69.6, CH
5''	77.0, CH	77.2, CH
6''	61.2, CH_2	60.7, CH_2

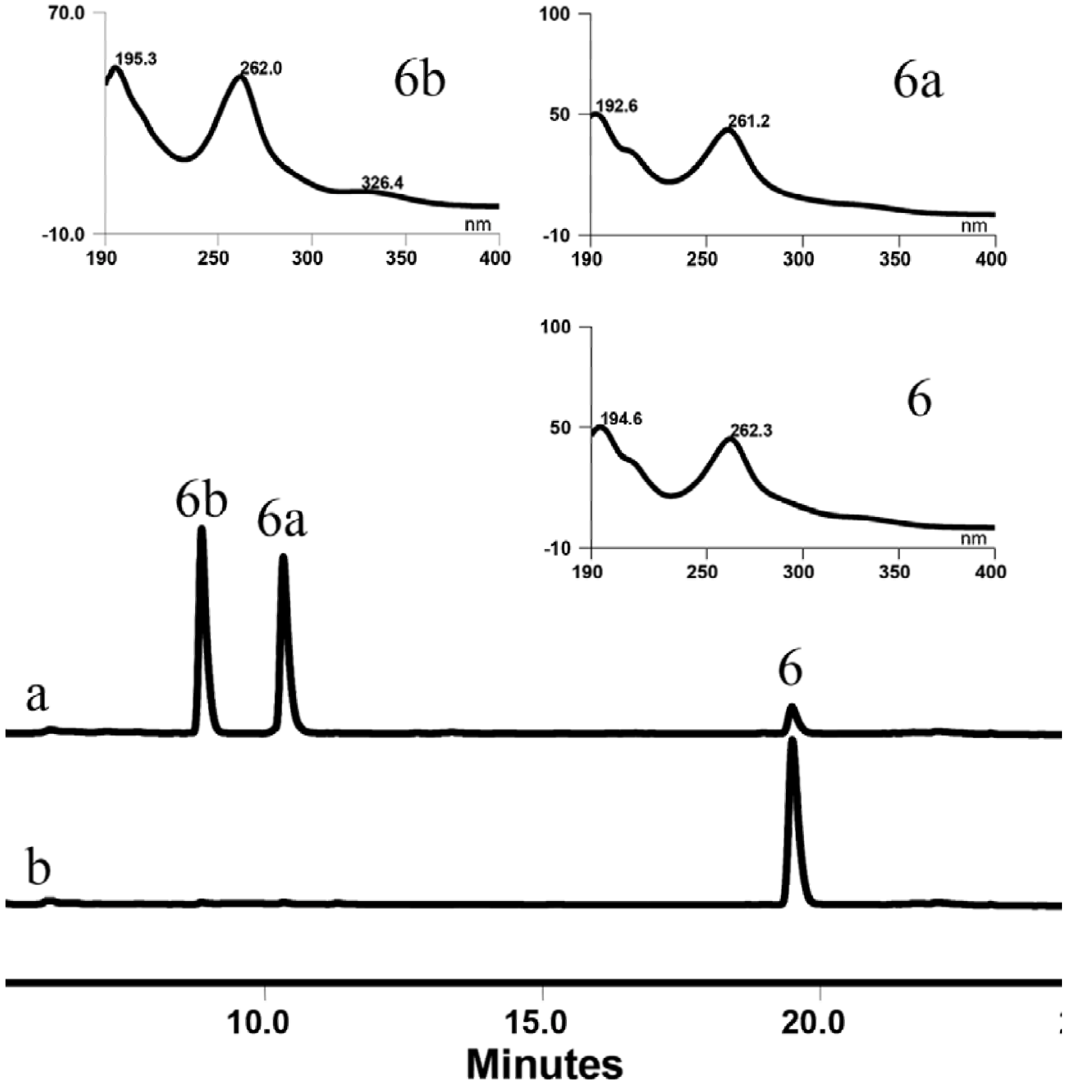
70.0
195.3
262.0
6b
326.4
-10.0
nm
190
250
300
350
400
100
192.6
6a
50
261.2
-10
nm
190
250
300
350
400
100
6
50
194.6
262.3
-10
nm
190
250
300
350
400
6b
6a
a
6
b
10.0
15.0
20.0
Minutes

Figure S1

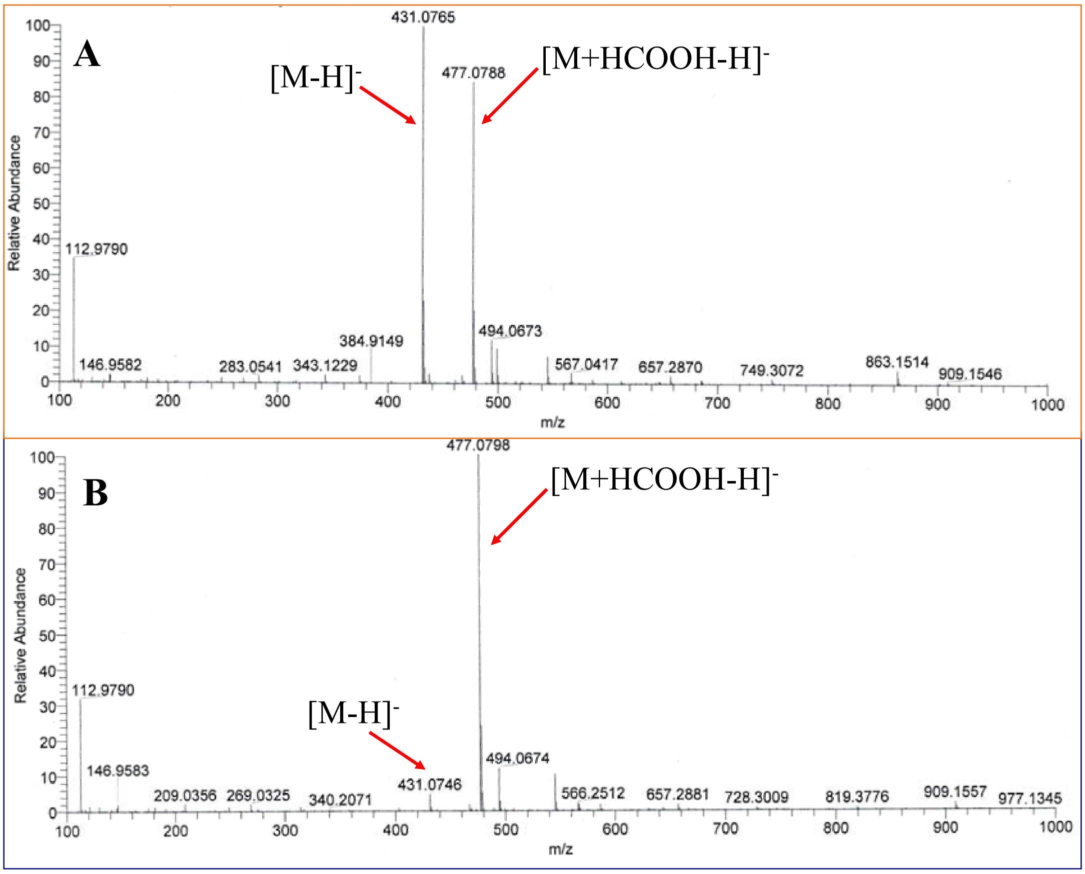
A
[M-H]-
[M+HCOOH-H]-
431.0765
477.0788
112.9790
146.9582
283.0541
343.1229
384.9149
494.0673
567.0417
657.2870
749.3072
863.1514
909.1546
Relative Abundance
m/z
B
[M+HCOOH-H]-
[M-H]-
477.0798
112.9790
146.9583
209.0356
269.0325
340.2071
431.0746
494.0674
566.2512
657.2881
728.3009
819.3776
909.1557
977.1345

Figure S2

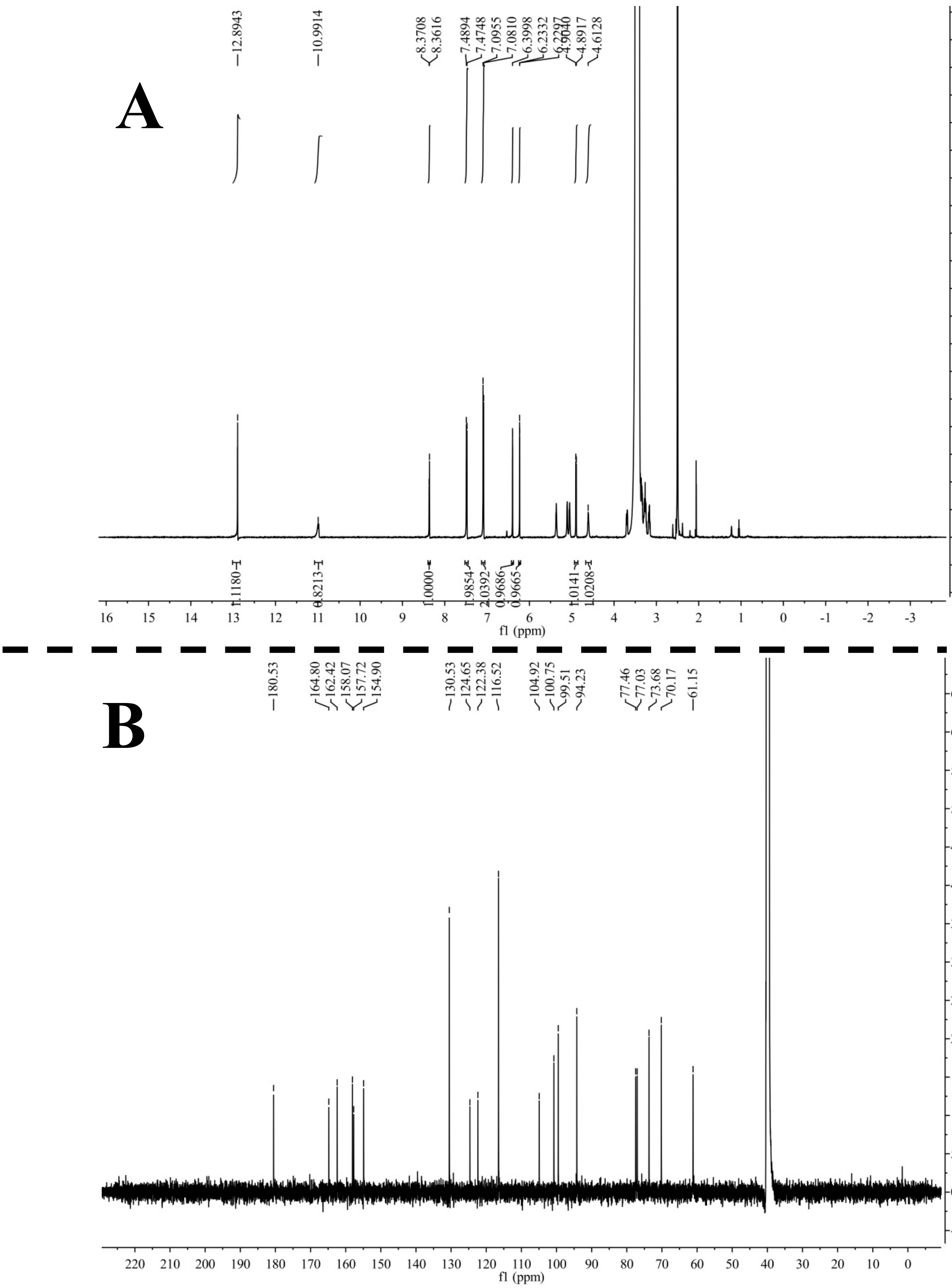

A
12.8943
10.9914
8.3708
8.3616
7.4894
7.4748
7.0955
7.0810
6.3998
6.2332
6.2297
4.9040
4.8917
4.6128
1.1180
0.8213
1.0000
1.9854
2.0392
0.9686
0.9665
1.0141
1.0208
f1 (ppm)
B
180.53
164.80
162.42
158.07
157.72
154.90
130.53
124.65
122.38
116.52
104.92
100.75
99.51
94.23
77.46
77.03
73.68
70.17
61.15
f1 (ppm)

Figure S3

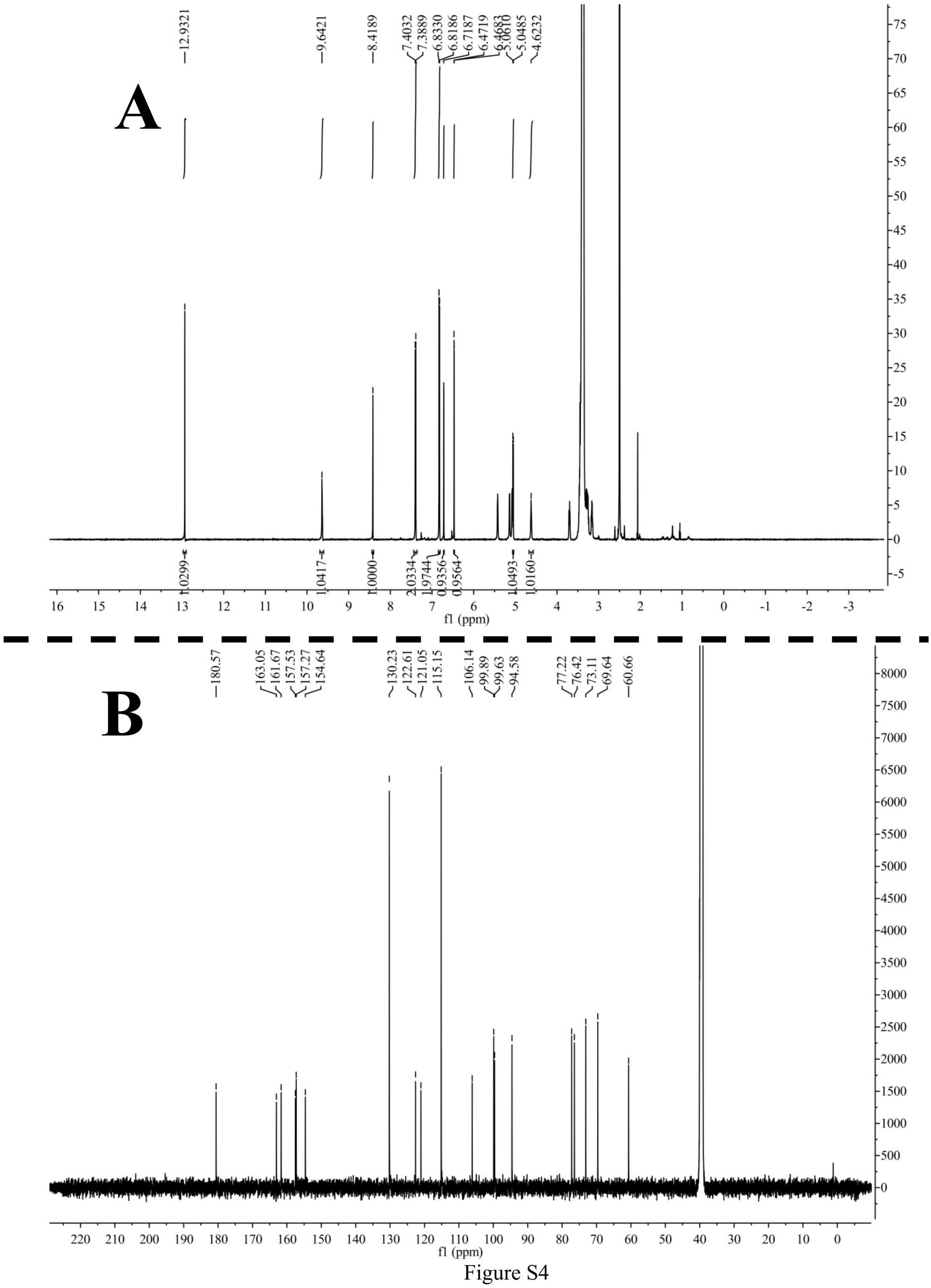

Figure S4

Supplementary result section IV

Section IV is a database presenting HPLC chromatogram (Figure S1), UV spectrum (Figure S1), mass spectrum (Figure S1) and NMR spectrum (Figures S2-3 and Tables S1-2) for compounds **7a** and **7b**.

Table S1 ^{1}H NMR (600 MHz) spectroscopic data (δ in ppm, J in Hz) for compounds **7a** and **7b** in DMSO-d_6

Table S2 ^{13}C NMR (150 MHz) spectroscopic data (δ in ppm, J in Hz) for compounds **7a** and **7b** in DMSO-d_6

Figure S1 HPLC chromatogram of glycosylated metabolites (**7a** and **7b**) of daidzein (**7**) with OcUGT1 (a) or without OcUGT1 (b). The UV absorption spectrum of **7a** or **7b** is similar to that of **7**. All of them are marked in the right panels. The mass spectra of **7a (A)** and **7b (B)** analyzed on ESI-TOF MS are displayed in the top panels.

Figure S2 ^{1}H NMR spectrum (600 MHz, DMSO-d_6) (**A**) and ^{13}C NMR spectrum of **7a** (150 MHz, DMSO-d_6) (**B**)

Figure S3 ^{1}H NMR spectrum (600 MHz, DMSO-d_6) (**A**) and ^{13}C NMR spectrum of **7b** (150 MHz, DMSO-d_6) (**B**)

Table S1

Position	**7a**	**7b**
2	8.35,1H,s	8.39,1H,s
5	7.98,1H,d(8.8Hz)	8.05,1H,d(8.9Hz)
6	6.95,1H,dd(8.8Hz,2.2Hz)	7.15,1H,dd(8.9Hz,2.3Hz)
8	6.88,1H,d(2.3Hz)	7.23,1H,d(2.3Hz)
2', 6'	7.50,2H,d(8.7Hz)	7.41,2H,d(8.7Hz)
3', 5'	7.09,2H,d(8.8Hz)	6.82,2H,d(8.6Hz)
	Glc	Glc
1''	4.91,1H,d(7.4Hz)	5.10,1H,d(7.3Hz)
H of sugar	3.10-3.60	3.00-3.70

Table S2

Position	**7a**	**7b**
2	153.3,CH	153.4,CH
3	123.1,C	123.7,C
4	174.6,C	174.8,C
5	127.3,CH	127.0,CH
6	115.3,CH	115.6,CH
7	162.6,C	161.4,C
8	102.2,CH	103.4,CH
9	157.5,C	157.3,C
10	116.6,C	118.5,C
1'	125.5,C	122.3,C
2', 6'	130.0,CH	130.1,CH
3', 5'	116.0,CH	115.0,CH
4'	157.1,C	157.1,C
	Glc	Glc
1''	100.4,CH	100.0,CH
2''	73.3,CH	73.2,CH
3''	76.7,CH	76.5,CH
4''	69.7,CH	70.0,CH
5''	77.1,CH	77.2,CH
6''	60.7,CH_2	60.7,CH_2

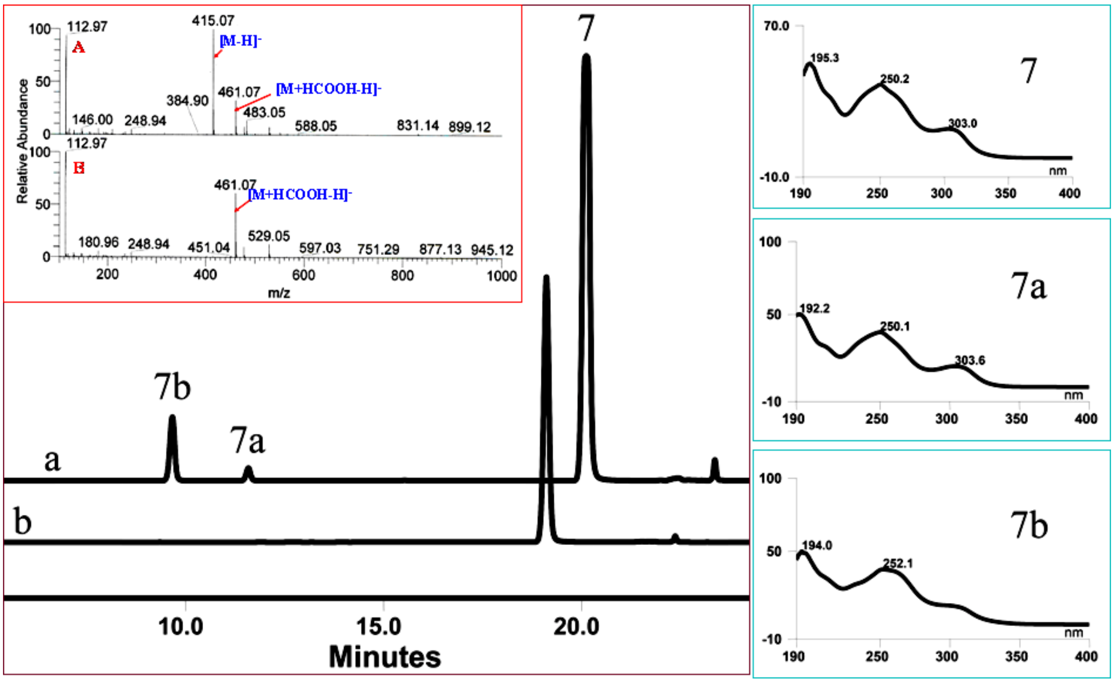
7
7b
7a
a
b
10.0
15.0
20.0
Minutes
Relative Abundance
m/z
A
B
112.97
415.07
[M-H]-
461.07
[M+HCOOH-H]-
384.90
483.05
146.00
248.94
588.05
831.14
899.12
180.96
451.04
529.05
597.03
751.29
877.13
945.12
195.3
250.2
303.0
7a
192.2
250.1
303.6
194.0
252.1
nm

Figure S1

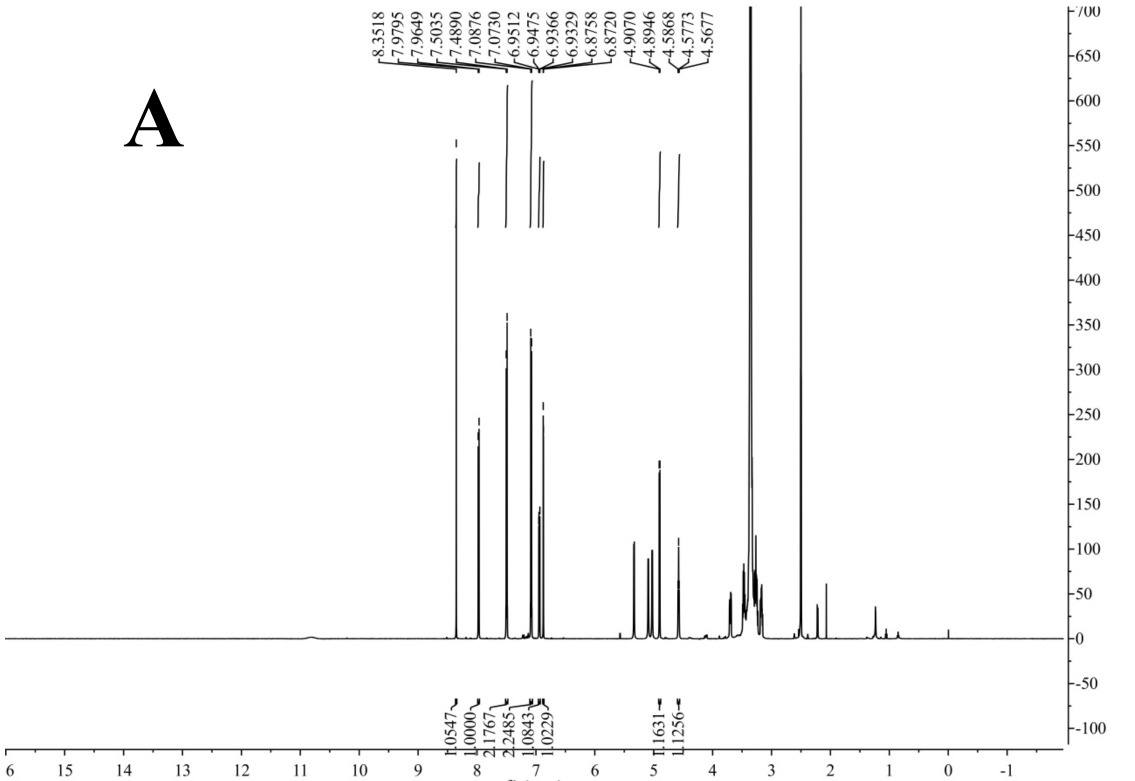
A
8.3518
7.9795
7.9649
7.5035
7.4890
7.0876
7.0730
6.9512
6.9475
6.9366
6.9329
6.8758
6.8720
4.9070
4.8946
4.5868
4.5773
4.5677
1.0547
1.0000
2.1767
2.2485
1.0843
1.0229
1.1631
1.1256
f1 (ppm)

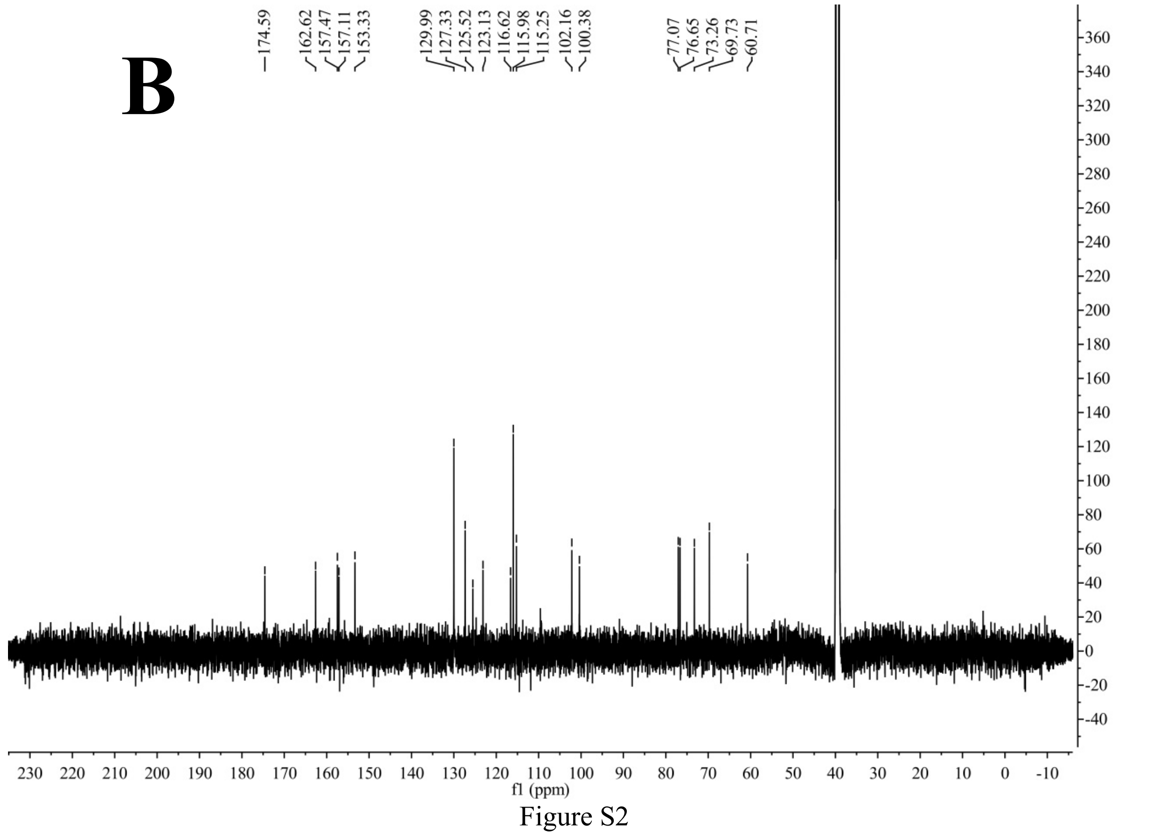
B
174.59
162.62
157.47
157.11
153.33
129.99
127.33
125.52
123.13
116.62
115.98
115.25
102.16
100.38
77.07
76.65
73.26
69.73
60.71
f1 (ppm)

Figure S2

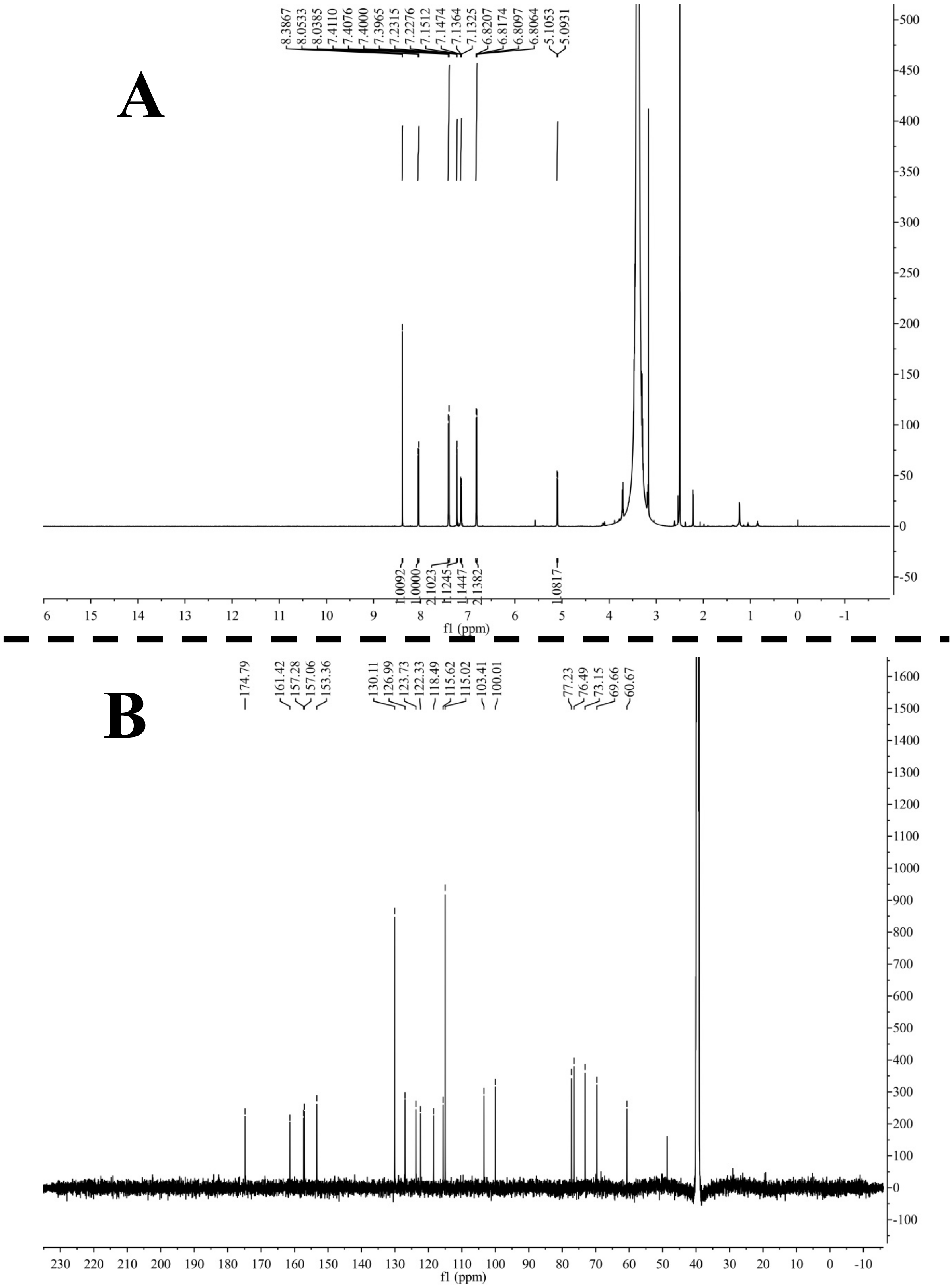
A
8.3867
8.0533
8.0385
7.4110
7.4076
7.4000
7.3965
7.2315
7.2276
7.1512
7.1474
7.1364
7.1325
6.8207
6.8174
6.8097
6.8064
5.1053
5.0931
1.0092
1.0000
2.1023
1.1245
1.1447
2.1382
1.0817
f1 (ppm)
B
174.79
161.42
157.28
157.06
153.36
130.11
126.99
123.73
122.33
118.49
115.62
115.02
103.41
100.01
77.23
76.49
73.15
69.66
60.67
f1 (ppm)

Figure S3

Supplementary result section V

Section V is a database presenting HPLC chromatogram (Figure S1), UV spectrum (Figure S1), mass spectrum (Figure S1) and NMR spectrum (Figure S2 and Table S1) for compound **3a**.

Table S1 ^{1}H NMR (600 MHz) and ^{13}C NMR (150 MHz) spectroscopic data (δ in ppm) for **3a** in DMSO-d_6

Figure S1 HPLC chromatogram of glycosylated metabolite (**3a**) of 6-hydroxyflavone (**3**) with OcUGT1 (a) or without OcUGT1 (b) (A). The UV absorption spectrum of **3a** is similar to that of **3**. Both are marked in the right panels (B). The mass spectra of **3a** analyzed on ESI-TOF MS is displayed in the bottom panel (C).

Figure S2 ^{1}H NMR spectrum (600 MHz, DMSO-d_6) (A) and ^{13}C NMR spectrum of **3a** (150 MHz, DMSO-d_6) (B)

Table S1

Position	*δ*C	*δ*H
2	162.7, C	
3	106.3, CH	7.02, 1H, s
4	177.1, C	
5	109.5, CH	7.60, 1H, m
6	151.2, C	
7	124.1, CH	7.54, 1H, dd(3.1Hz, 9.1Hz)
8	120.2, CH	7.79, 1H, d(6.7Hz)
9	154.8, C	
10	124.3, C	
1'	131.3, C	
2'	126.5, CH	8.11, 1H, d(1.7Hz)
3'	129.3, CH	7.60, 1H, m
4'	132.0, CH	7.60, 1H, m
5'	129.3, CH	7.60, 1H, m
6'	126.5, CH	8.11, 1H, d(1.7Hz)
1''	101.1, CH	4.98, 1H, d(7.5Hz)
2''	73.4, CH	
3''	76.5, CH	
4''	69.7, CH	
5''	77.2, CH	
6''	60.7, CH_2	
		H of Sugar 3.10-3.70

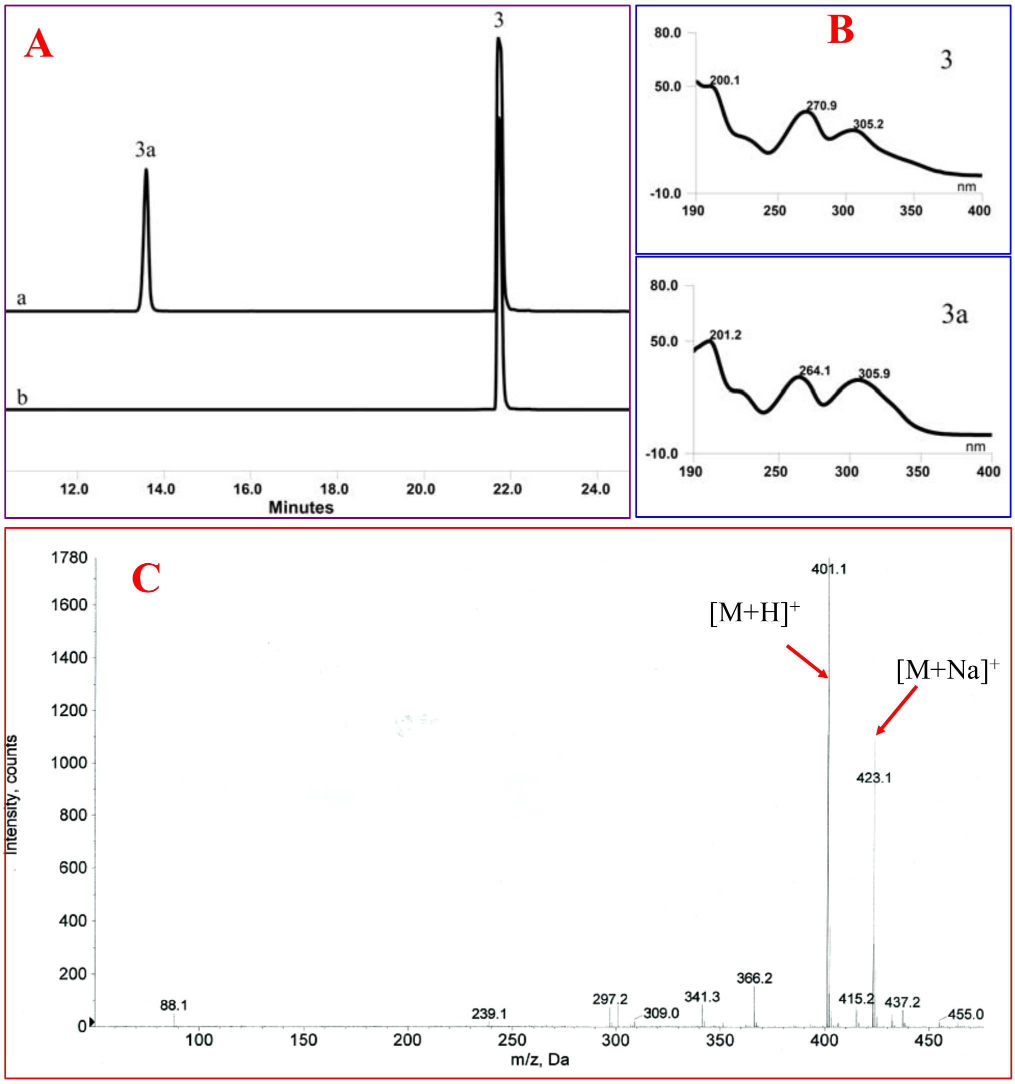
A
3a
3
a
b
12.0
14.0
16.0
18.0
20.0
22.0
24.0
Minutes
B
3
80.0
50.0
-10.0
200.1
270.9
305.2
nm
190
250
300
350
400
3a
201.2
264.1
305.9
C
1780
1600
1400
1200
1000
800
600
400
200
0
Intensity, counts
401.1
[M+H]+
[M+Na]+
423.1
88.1
239.1
297.2
309.0
341.3
366.2
415.2
437.2
455.0
100
150
200
250
300
350
400
450
m/z, Da

Figure S1

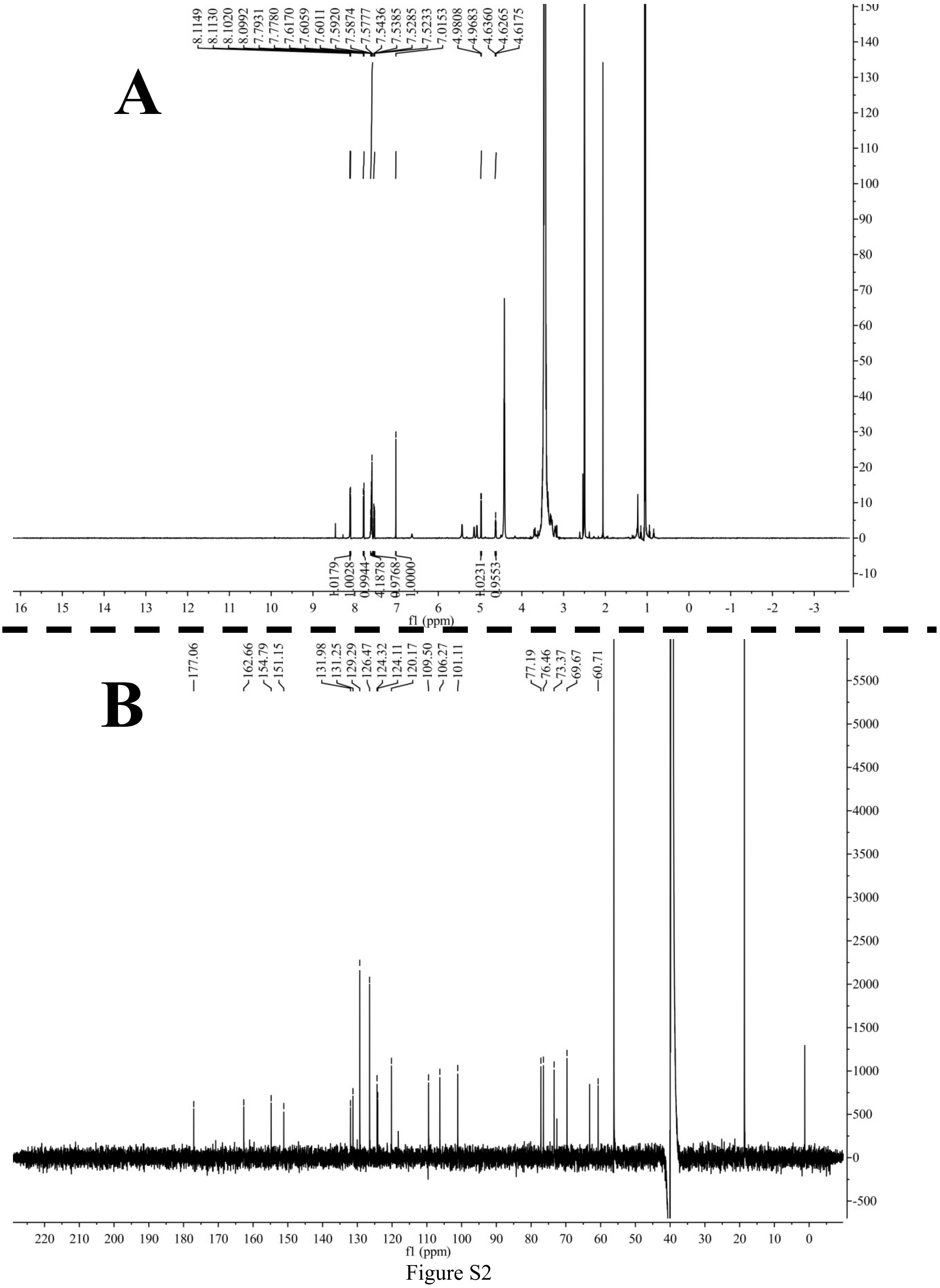
A
8.1149
8.1130
8.1020
8.0992
7.7931
7.7780
7.6170
7.6059
7.6011
7.5920
7.5874
7.5777
7.5436
7.5385
7.5285
7.5233
7.0153
4.9808
4.9683
4.6360
4.6265
4.6175
1.0179
1.0028
0.9944
4.1878
0.9768
1.0000
1.0231
0.9553
f1 (ppm)
B
177.06
162.66
154.79
151.15
131.98
131.25
129.29
126.47
124.32
124.11
120.17
109.50
106.27
101.11
77.19
76.46
73.37
69.67
60.71
f1 (ppm)

Figure S2

Supplementary result section VI

Section VI is a database presenting HPLC chromatogram (Figure S1), UV spectrum (Figure S1), mass spectrum (Figure S1) and NMR spectrum (Figures S2-3 and Tables S1-2) for compounds **4a-4c**.

Table S1 ^{1}H NMR (600 MHz) spectroscopic data (δ in ppm, J in Hz) for compounds **4a** and **4b** in DMSO-d_6

Table S2 ^{13}C NMR (150 MHz) spectroscopic data (δ in ppm, J in Hz) for compounds **4a** and **4b** in DMSO-d_6

Figure S1 HPLC chromatogram of glycosylated metabolites (**4a-4c**) of 2'-hydroxyflavonol (**4**) with OcUGT1 (a) or without OcUGT1 (b) (B). The UV absorption spectra of **4a-4c** are similar to that of **4**. All are marked in the top panels **(A)**. The mass spectra of **4a-4c** analyzed on ESI-TOF MS were displayed right panels (**C-E**).

Figure S2 ^{1}H NMR spectrum (600 MHz, DMSO-d_6) (A) and ^{13}C NMR spectrum of **4a** (150 MHz, DMSO-d_6) (B)

Figure S3 ^{1}H NMR spectrum (600 MHz, DMSO-d_6) (A) and ^{13}C NMR spectrum of **4b** (150 MHz, DMSO-d_6) (B)

Table S1

Position	**4a**	**4b**
5	8.14, 1H, d(8.0Hz)	8.14, 1H, d(8.0Hz)
7	7.77, 1H, m	7.83, 1H, t(8.0Hz)
8	7.63, 1H, d(8.6Hz)	7.67, 1H, m
6'	7.51, 1H, m	7.67, 1H, m
6	7.55, 1H, dd(7.6Hz,1.7Hz)	7.53, 1H, t(7.4Hz)
4'	7.32, 1H, d(8.7Hz)	7.35, 1H, t(8.7Hz)
3'	7.51, 1H, m	6.96, 1H, d(8.2Hz)
5'	7.15, 1H, t(7.3Hz)	6.90, 1H, t(7.5Hz)
	Glc	Glc
1''	5.01, 1H, m	5.28, 1H, d(7.9Hz)
H of sugar	3.00-3.70	2.90-3.60

Table S2

Position	**4a**	**4b**
2	150.0, C	157.6, C
3	138.9, C	137.1, C
4	173.0, C	173.7, C
5	124.8, CH	125.1, CH
6	124.3, CH	125.0, CH
7	133.4, CH	134.1, CH
8	118.1, CH	118.4, CH
9	155.1, C	155.2, C
10	122.1, C	123.6, C
1'	118.6, C	118.0, C
2'	155.5, C	155.5, C
3'	115.3, CH	116.0, CH
4'	131.1, CH	131.6, CH
5'	120.5, CH	118.4, CH
6'	127.6, CH	132.0, CH
	Glc	Glc
1''	100.4, CH	100.9, CH
2''	75.0, CH	74.0, CH
3''	76.6, CH	76.5, CH
4''	73.3, CH	69.8, CH
5''	77.1, CH	77.2, CH
6''	69.7, CH_2	61.0, CH_2

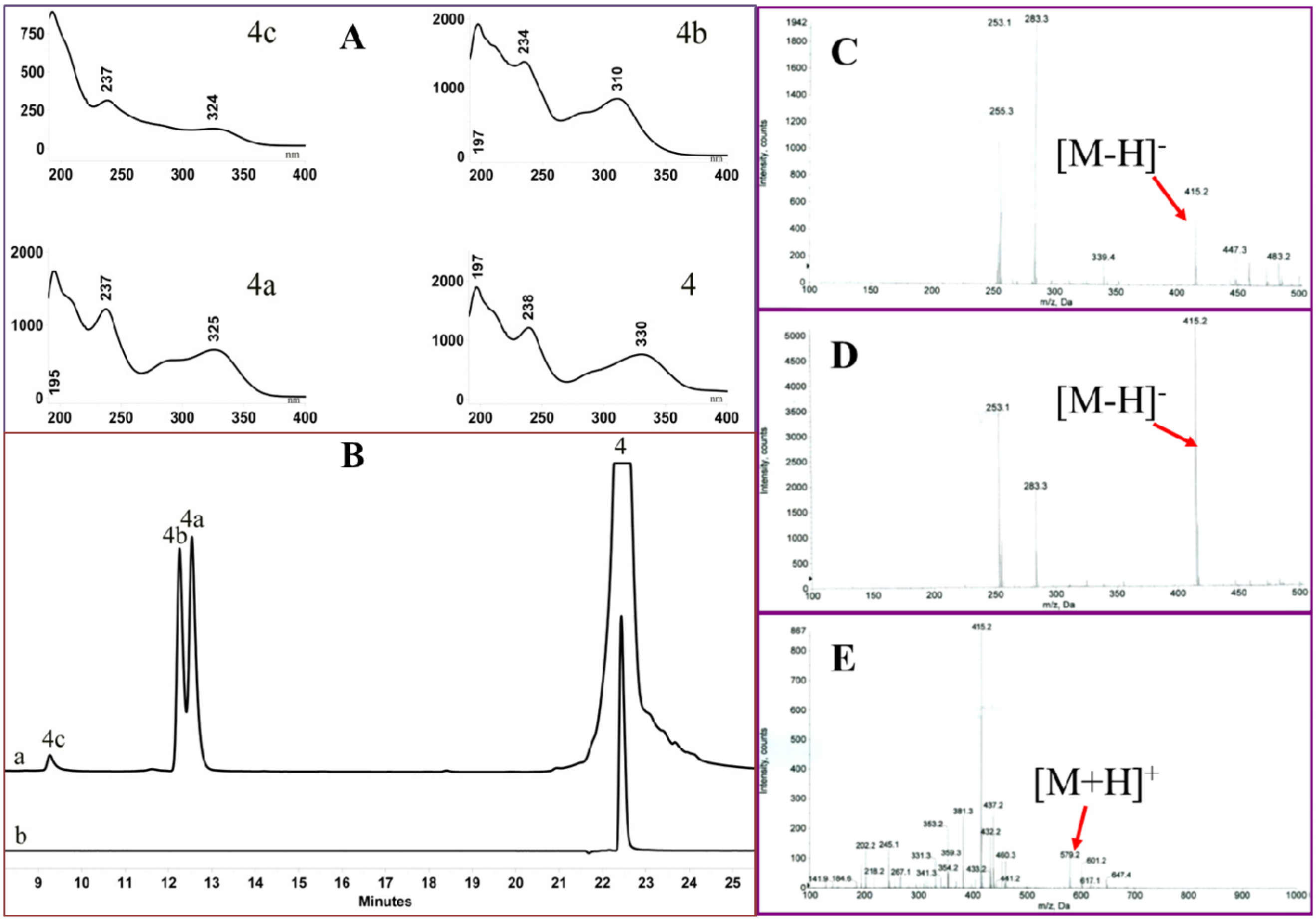
A
4c
4b
4a
4
237
324
234
310
197
195
325
238
330
200
250
300
350
400
750
500
250
0
2000
1000
B
4b
4a
4c
a
b
9
10
11
12
13
14
15
16
17
18
19
20
21
22
23
24
25
Minutes
C
[M-H]-
253.1
283.3
255.3
415.2
339.4
447.3
483.2
D
[M-H]-
415.2
253.1
283.3
E
[M+H]+
415.2
381.3
437.2
363.2
432.2
202.2
245.1
331.3
359.3
460.3
579.2
601.2
218.2
267.1
341.3
354.2
433.2
441.2
617.1
647.4
Intensity, counts
m/z, Da

Figure S1

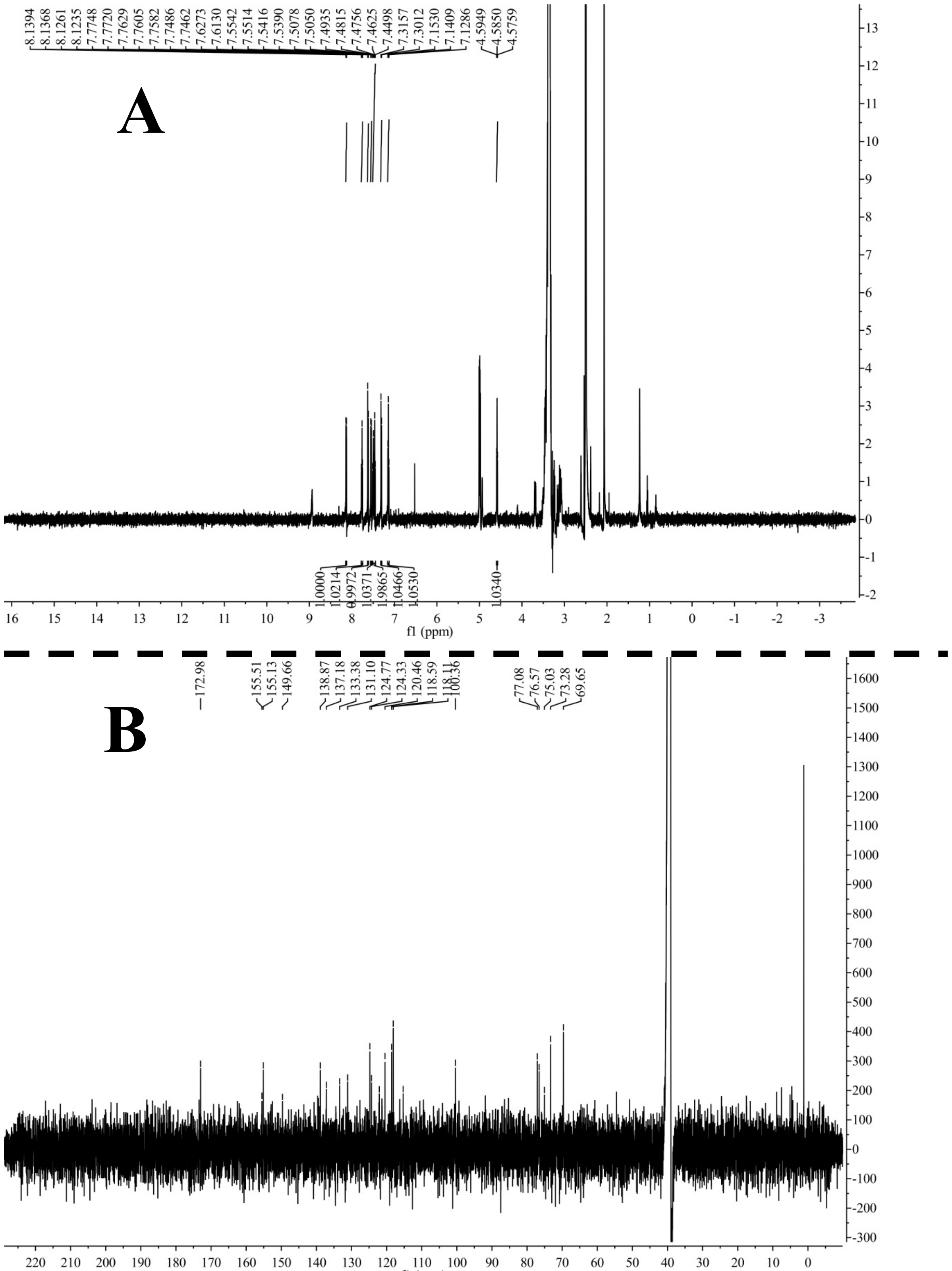

Figure S2

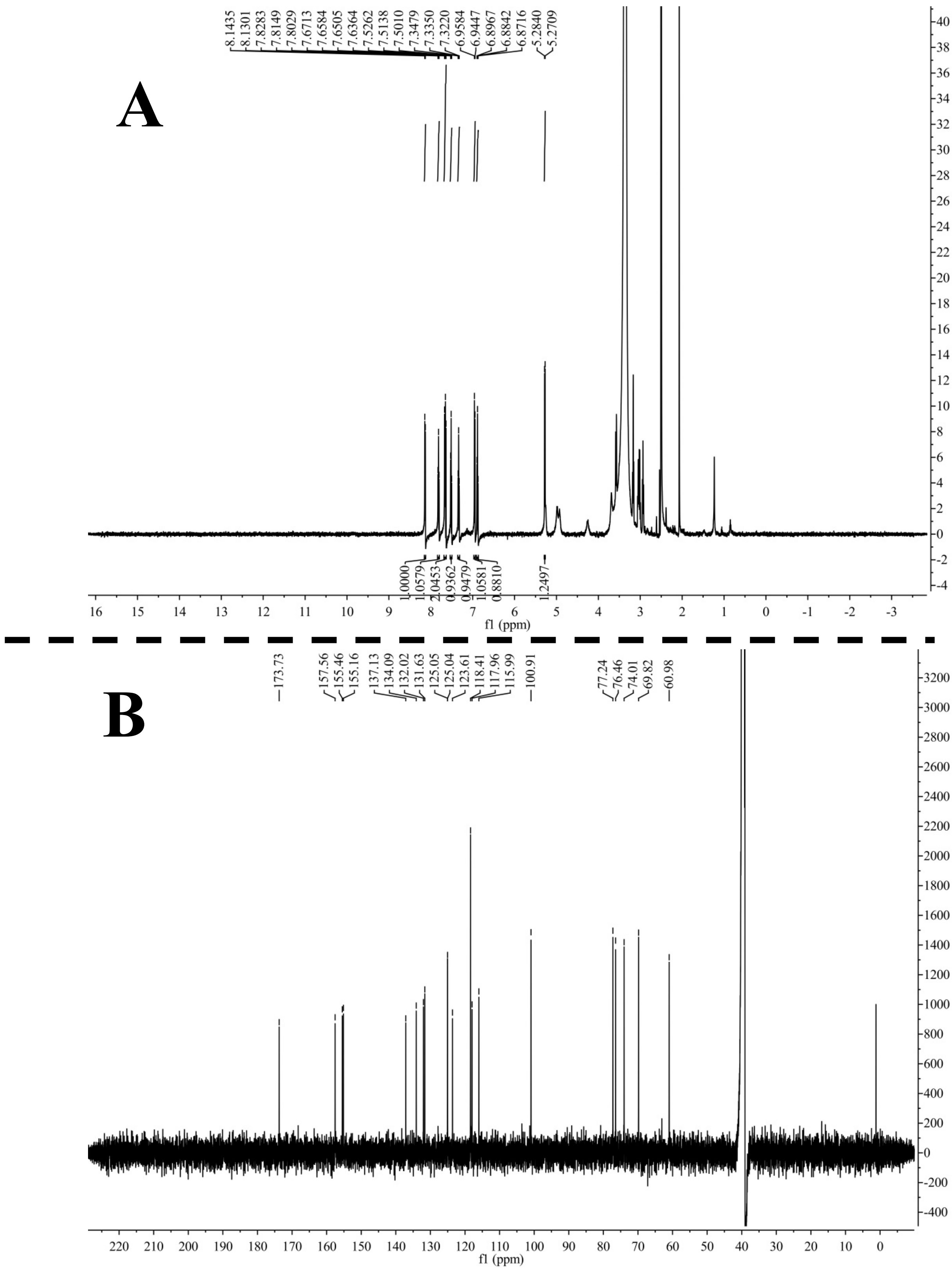
A
8.1435
8.1301
7.8283
7.8149
7.8029
7.6713
7.6584
7.6505
7.6364
7.5262
7.5138
7.5010
7.3479
7.3350
7.3220
6.9584
6.9447
6.8967
6.8842
6.8716
5.2840
5.2709
1.0000
1.0579
2.0453
0.9362
0.9479
1.0581
0.8810
1.2497
f1 (ppm)
B
173.73
157.56
155.46
155.16
137.13
134.09
132.02
131.63
125.05
125.04
123.61
118.41
117.96
115.99
100.91
77.24
76.46
74.01
69.82
60.98
f1 (ppm)

Figure S3

Supplementary result section VII

Section VII is a database presenting HPLC chromatogram, UV spectrum (Figure S1) and mass spectrum for compounds **5a** (Figure S1), **8a** (Figure S2), **9a** and **9b** (Figure S3).

Figure S1 HPLC chromatogram of glycosylated metabolite (**5a**) of 7-hydroxyflavone (**5**) with OcUGT1 (a) or without OcUGT1 (b). The UV absorption spectrum of **5a** is similar to that of **5**. Both are marked in the top panels (A). The mass spectrum of **5a** analyzed on ESI-TOF MS was displayed in the bottom panel (B)

Figure S2 HPLC chromatogram of glycosylated metabolite (**8a**) of pinocembrin (**8**) with OcUGT1 (a) or without OcUGT1 (b). The UV absorption spectrum of **8a** is similar to that of **8**. Both are marked in the top panels (A). The mass spectrum of **8a** analyzed on ESI-TOF MS was displayed in the bottom panel (B)

Figure S3 HPLC chromatogram of glycosylated metabolites (**9a-9b**) of naringenin (**9**) with OcUGT1 (a) or without OcUGT1 (b) (**A**). The UV absorption spectra of **9a** and **9b** are similar to that of **9**. Both are marked in the right panels (**B-D**). The mass spectra of **9a** and **9b** analyzed on ESI-TOF MS were displayed in the bottom panels (**E-F**).

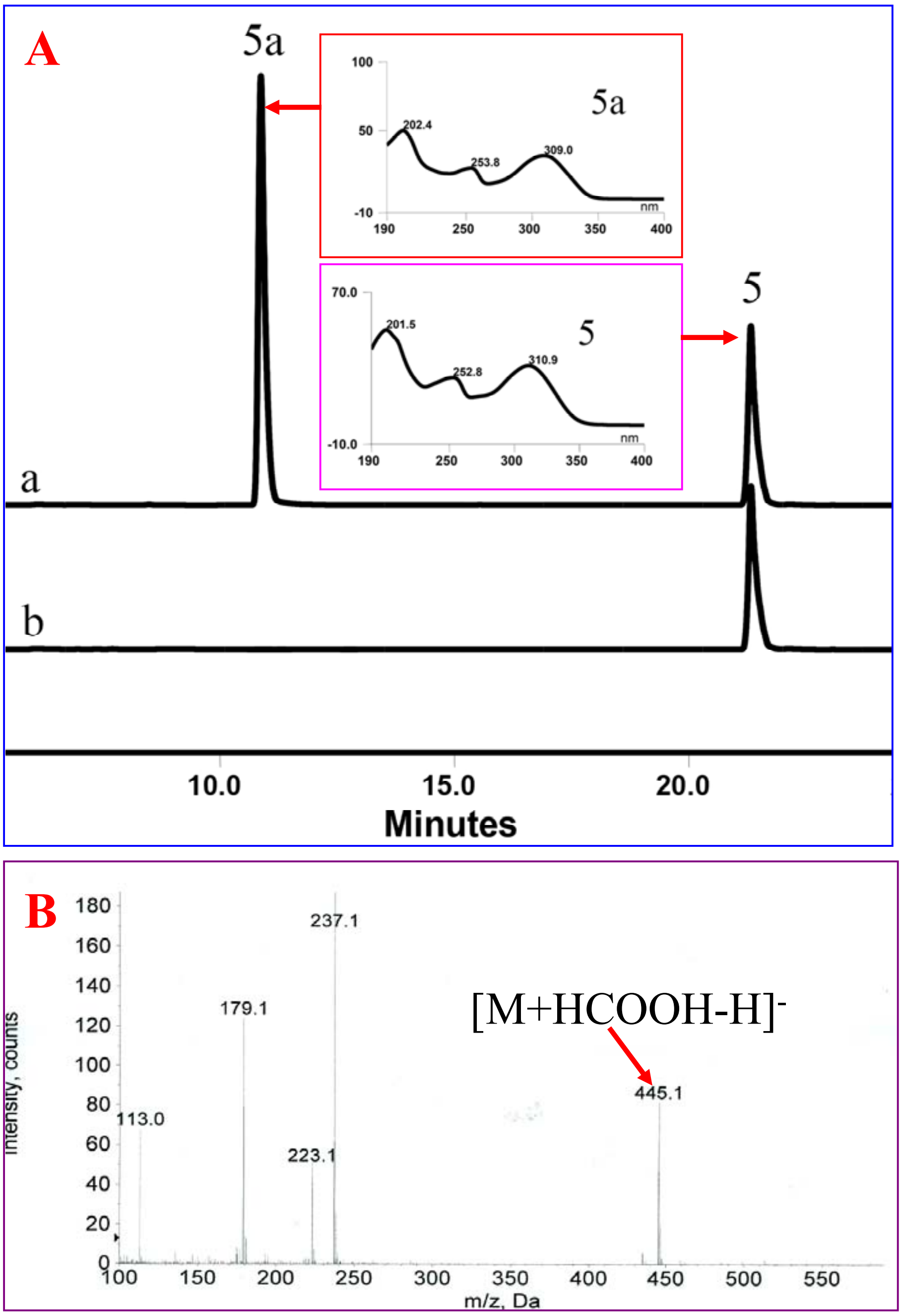
A
5a
100
50
-10
202.4
253.8
309.0
nm
190
250
300
350
400
5
70.0
-10.0
201.5
252.8
310.9
a
b
10.0
15.0
20.0
Minutes
B
180
160
140
120
100
80
60
40
20
0
Intensity, counts
237.1
179.1
113.0
223.1
445.1
[M+HCOOH-H]-
100
150
200
250
300
350
400
450
500
550
m/z, Da

Figure S1

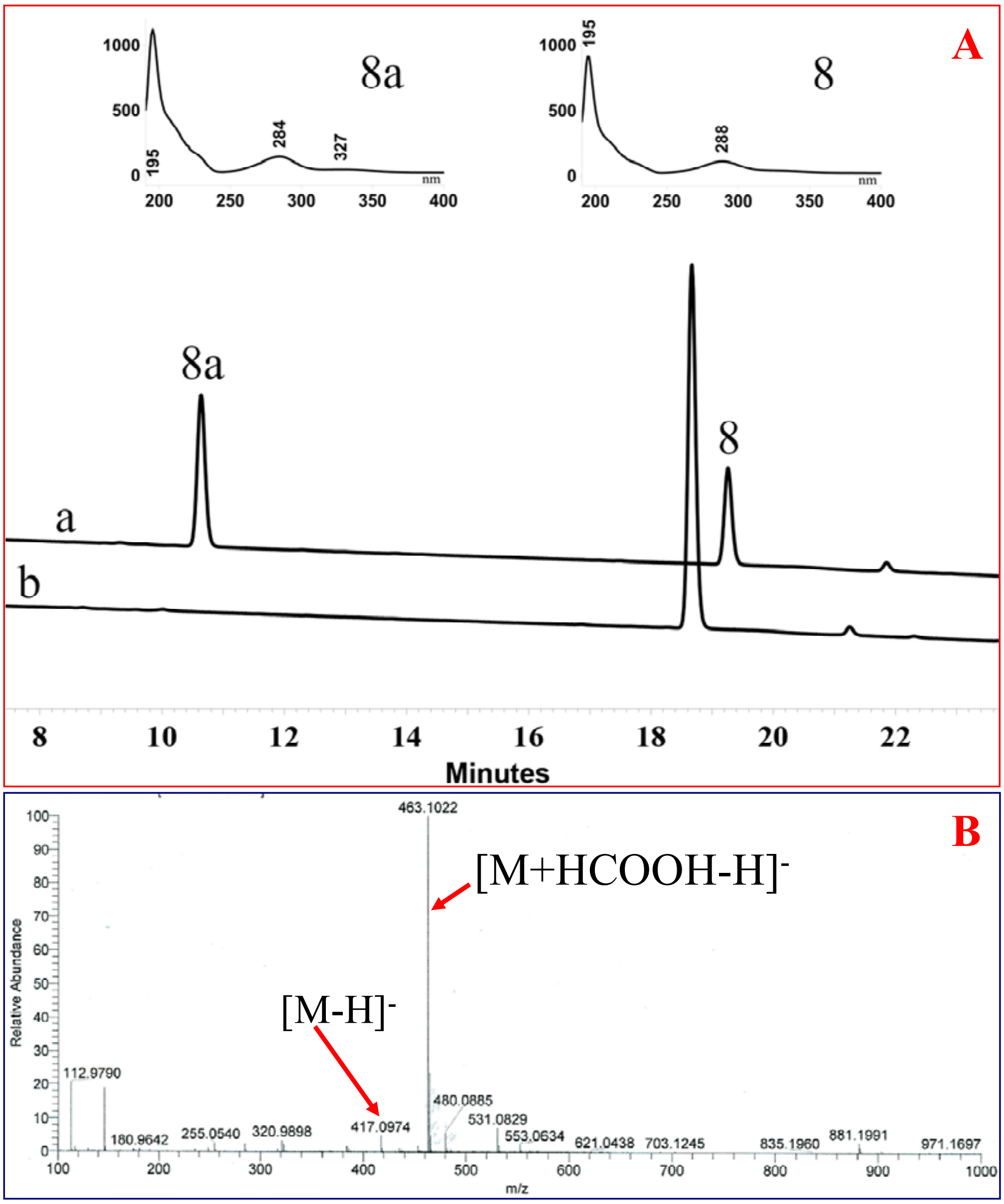

A
8a
8
1000
500
0
195
284
327
288
nm
200
250
300
350
400
a
b
8
10
12
14
16
18
20
22
Minutes
B
463.1022
[M+HCOOH-H]-
[M-H]-
Relative Abundance
112.9790
180.9642
255.0540
320.9898
417.0974
480.0885
531.0829
553.0634
621.0438
703.1245
835.1960
881.1991
971.1697
m/z

Figure S2

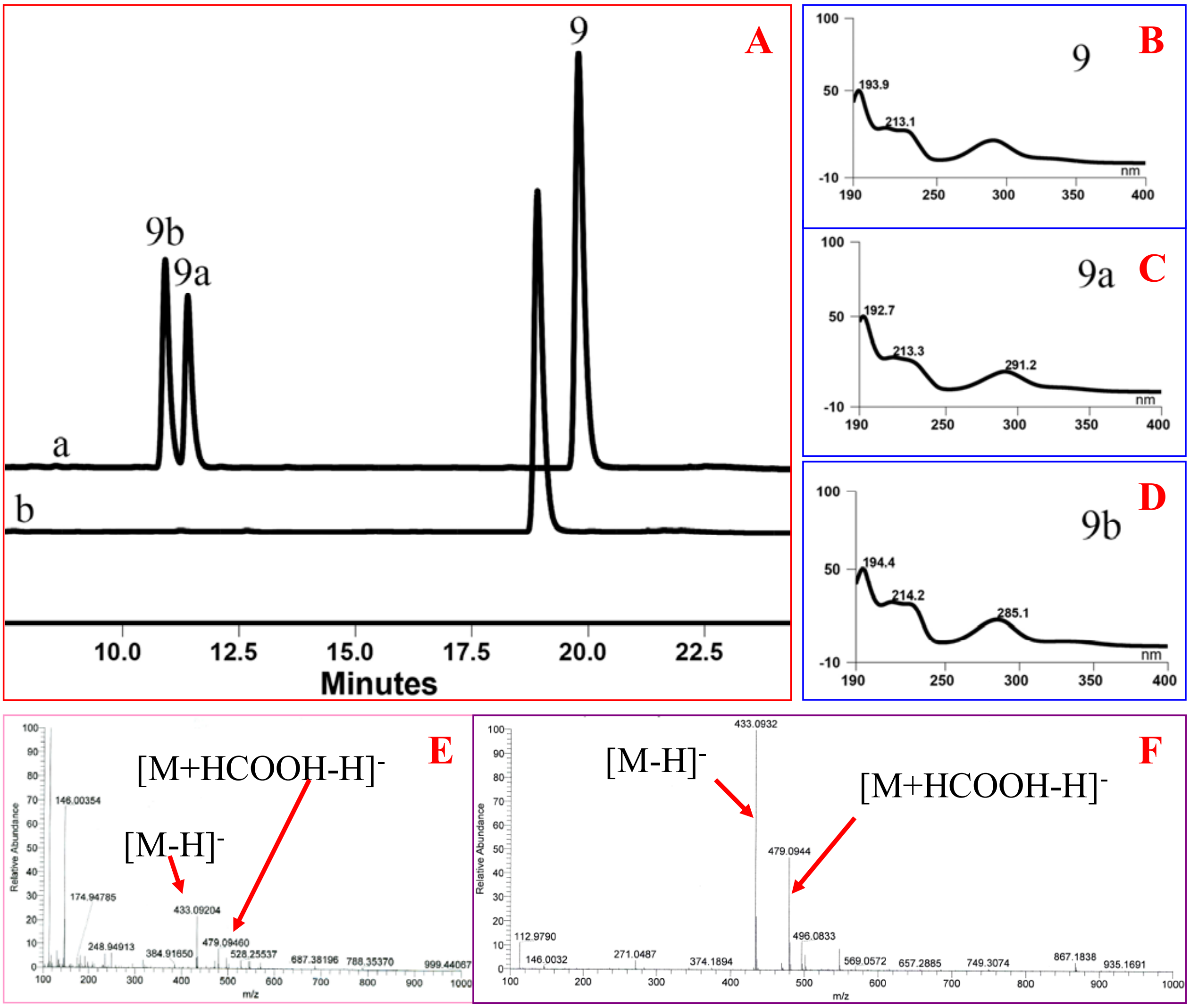

A
9
9b
9a
a
b
10.0
12.5
15.0
17.5
20.0
22.5
Minutes
B
9
193.9
213.1
C
9a
192.7
213.3
291.2
D
9b
194.4
214.2
285.1
E
[M+HCOOH-H]-
[M-H]-
146.00354
174.94785
433.09204
479.09460
F
[M-H]-
[M+HCOOH-H]-
433.0932
479.0944
496.0833
Relative Abundance
m/z

Figure S3

Supplementary result section VIII

Section VIII is a database presenting HPLC chromatogram (Figure S1), UV spectrum (Figure S1), mass spectrum (Figure S1) and NMR spectrum (Figure S2 and Table S1) for compound **10a**.

Table S1 ^{1}H NMR (600 MHz) and ^{13}C NMR (150 MHz) spectroscopic data (δ in ppm) for **10a** in DMSO-d_6

Figure S1 HPLC chromatogram of glycosylated metabolite **10a** of emodin (**10**) with OcUGT1 (a) or without OcUGT1 (b). The UV absorption spectrum of **10a** is similar to that of **10**. Both are marked in the top panels (A). The mass spectrum of **10a** analyzed on ESI-TOF MS was displayed in the bottom panel (B).

Figure S2 ^{1}H NMR spectrum (600 MHz, DMSO-d_6) (A) and ^{13}C NMR spectrum of **10a** (150 MHz, DMSO-d_6) (B)

Table S1

Position	δC	δH
1	161.5, C	1-OH 11.96, 1H, s
2	108.9, CH	7.23, 1H, s
3	164.0, C	
4	108.9, CH	7.55, 1H, s
5	120.6, CH	7.28, 1H, d(2.4Hz)
6	148.7, C	
7	124.2, CH	6.97, 1H, d(2.4Hz)
8	163.9, C	8-OH 12.14, 1H, s
9	190.2, C	
10	181.1, C	
4a	132.9, C	
8a	113.5, C	
9a	110.8, C	
10a	134.9, C	
1'	100.0, CH	5.15, 1H, d(7.5Hz)
2'	73.1, CH	
3'	77.3, CH	
4'	69.5, CH	
5'	76.2, CH	
6'	60.5, CH_2	
		$-CH_3$ 2.44, 3H, s
		H of Sugar 3.10-3.70

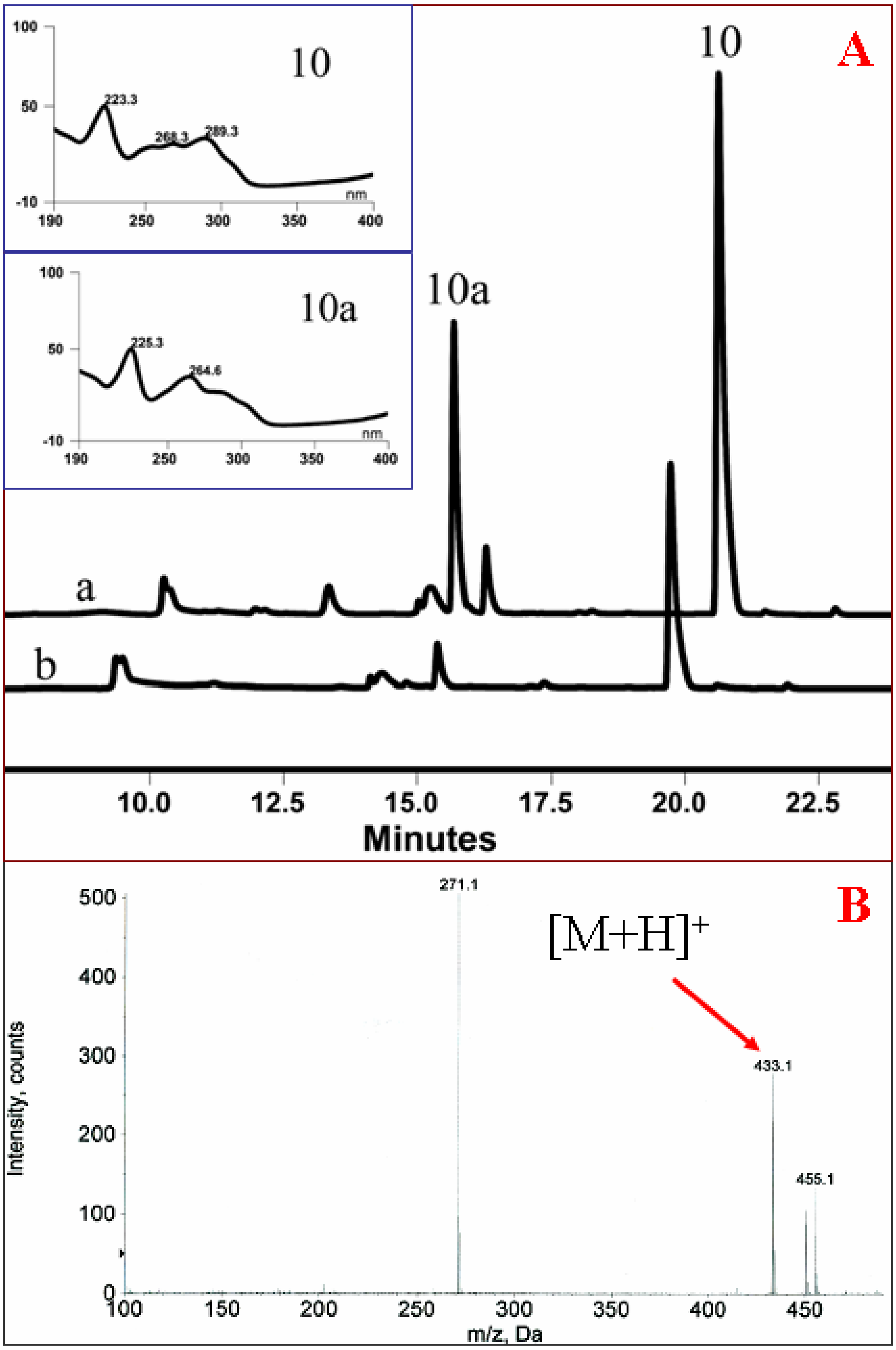

10
10a
A
a
b
Minutes
10.0
12.5
15.0
17.5
20.0
22.5
223.3
268.3
289.3
264.6
225.3
nm
B
[M+H]+
271.1
433.1
455.1
Intensity, counts
m/z, Da

Figure S1

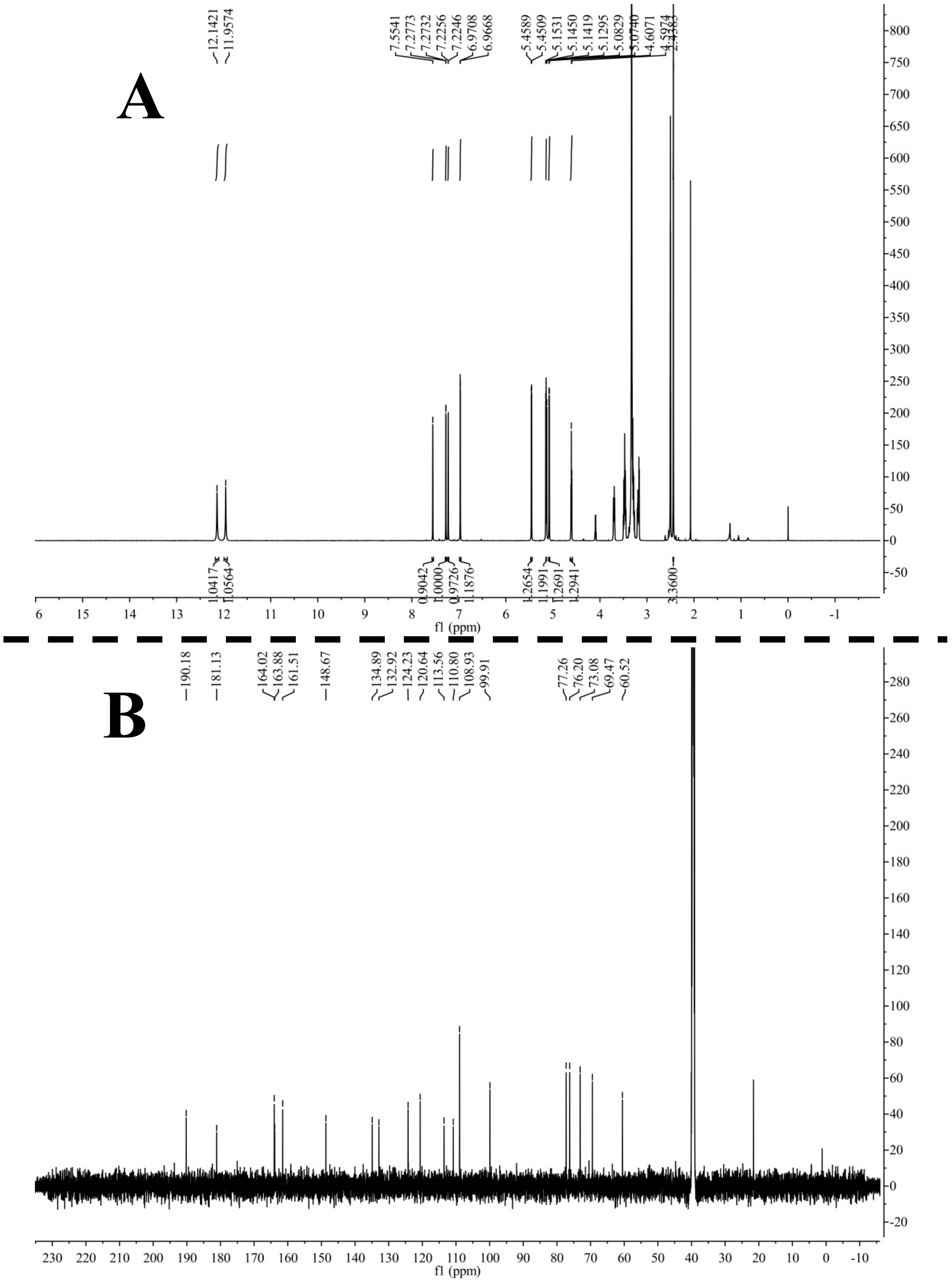

Figure S2

Supplementary result section IX

Section IX is a database presenting HPLC chromatogram (Figure S1), UV spectrum (Figure S1), mass spectrum (Figure S1) and NMR spectrum (Figures S2-3 and Tables S1-2) for compounds **11a-11b**.

Table S1 ^{1}H NMR (600 MHz) spectroscopic data (δ in ppm, J in Hz) for compounds **11a** and **11b** in DMSO-d_6

Table S2 ^{13}C NMR (150 MHz) spectroscopic data (δ in ppm, J in Hz) for compounds **11a** and **11b** in DMSO-d_6

Figure S1 HPLC chromatogram of glycosylated metabolite **11a** of resveratrol (**11**) with OcUGT1 (a) or without OcUGT1 (b) (A). The UV absorption spectrum of **11a** or **11b** is similar to that of **11**. All are marked in the right panels (B-D). The mass spectra of **11a** and **11b** analyzed on ESI-TOF MS were displayed in the lower left panel (E).

Figure S2 ^{1}H NMR spectrum (600 MHz, DMSO-d_6) (A) and ^{13}C NMR spectrum of **11a** (150 MHz, DMSO-d_6) (B)

Figure S3 ^{1}H NMR spectrum (600 MHz, DMSO-d_6) (A) and ^{13}C NMR spectrum of **11b** (150 MHz, DMSO-d_6) (B)

Table S1

Position	**11a**	**11b**
2', 6'	7.38,2H,d(8.6Hz)	7.46,2H,d(8.7Hz)
3', 5'	6.77,2H,d(8.6Hz)	7.09,2H,d(8.7Hz)
α	7.03,1H,d(16.2Hz)	7.01,1H,d(16.3Hz)
β	6.86,1H,d(16.2Hz)	6.89,1H,d(16.3Hz)
2, 6	6.79,1H,m, 6.61,1H,m	6.46,2H,d(2.1Hz)
4	6.45,1H,t(2.1Hz)	6.18,1H,t(2.1Hz)
	Glc	Glc
H of sugar	3.10-3.60	3.00-3.70

Table S2

Position	**11a**	**11b**
1	141.4,C	141.0,C
2	108.3,CH	105.9,CH
3	160.5,C	159.7,C
4	104.1,CH	102.2,CH
5	158.5,C	159.7,C
6	107.0,CH	105.9,CH
1'	130.3,C	133.2,C
2'	128.9,CH	128.6,CH
3'	116.5,CH	117.9,CH
4'	159.6,C	158.7,C
5'	116.5,CH	117.9,CH
6'	128.9,CH	128.6,CH
C-α	130.0,CH	128.9,CH
C-β	126.7,CH	128.5,CH
	Glc	Glc
1"	102.4,CH	102.9,CH
2"	75.0,CH	74.9,CH
3"	78.3,CH	78.2,CH
4"	71.5,CH	71.4,CH
5"	78.1,CH	78.0,CH
6"	62.6,CH_2	62.5,CH_2

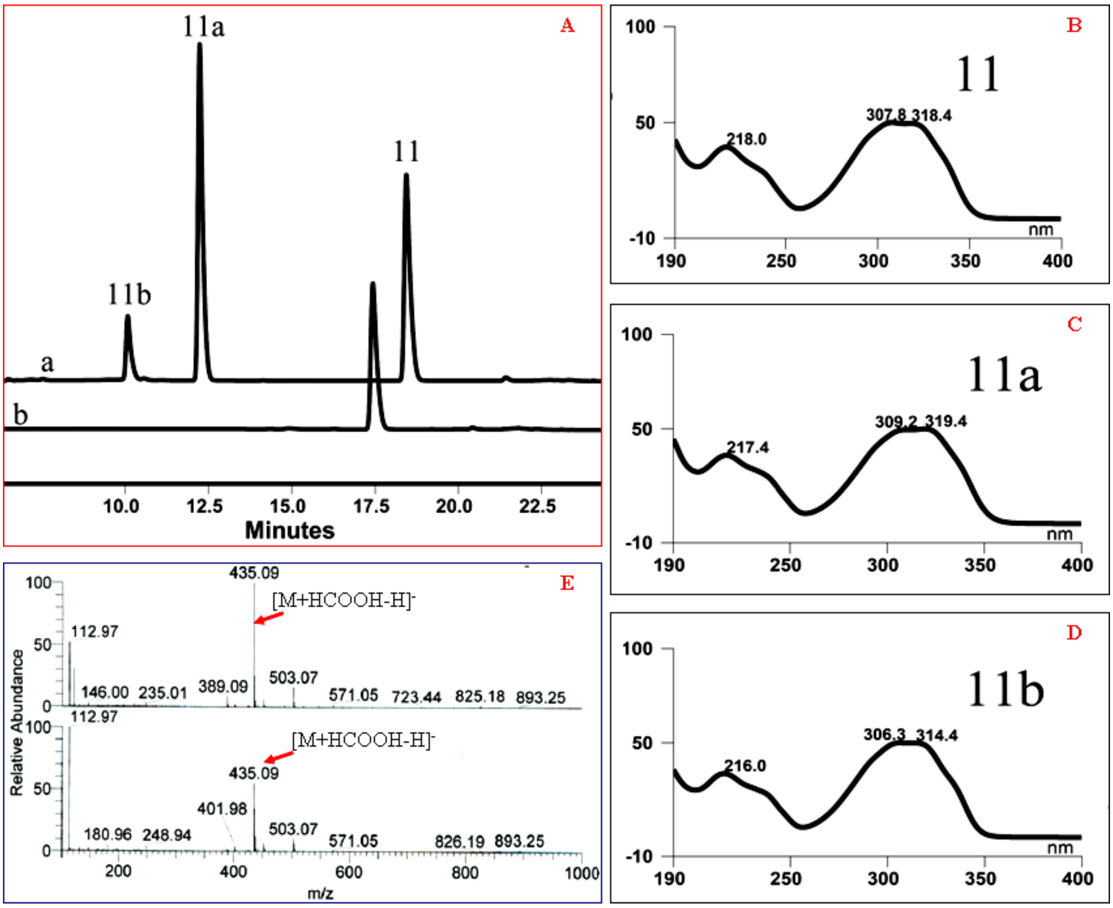
A
11a
11b
11
a
b
10.0
12.5
15.0
17.5
20.0
22.5
Minutes
B
11
100
50
-10
218.0
307.8 318.4
nm
190
250
300
350
400
C
11a
217.4
309.2 319.4
D
11b
216.0
306.3 314.4
E
Relative Abundance
435.09
[M+HCOOH-H]-
112.97
146.00
235.01
389.09
503.07
571.05
723.44
825.18
893.25
180.96
248.94
401.98
826.19
m/z
200
400
600
800
1000

Figure S1

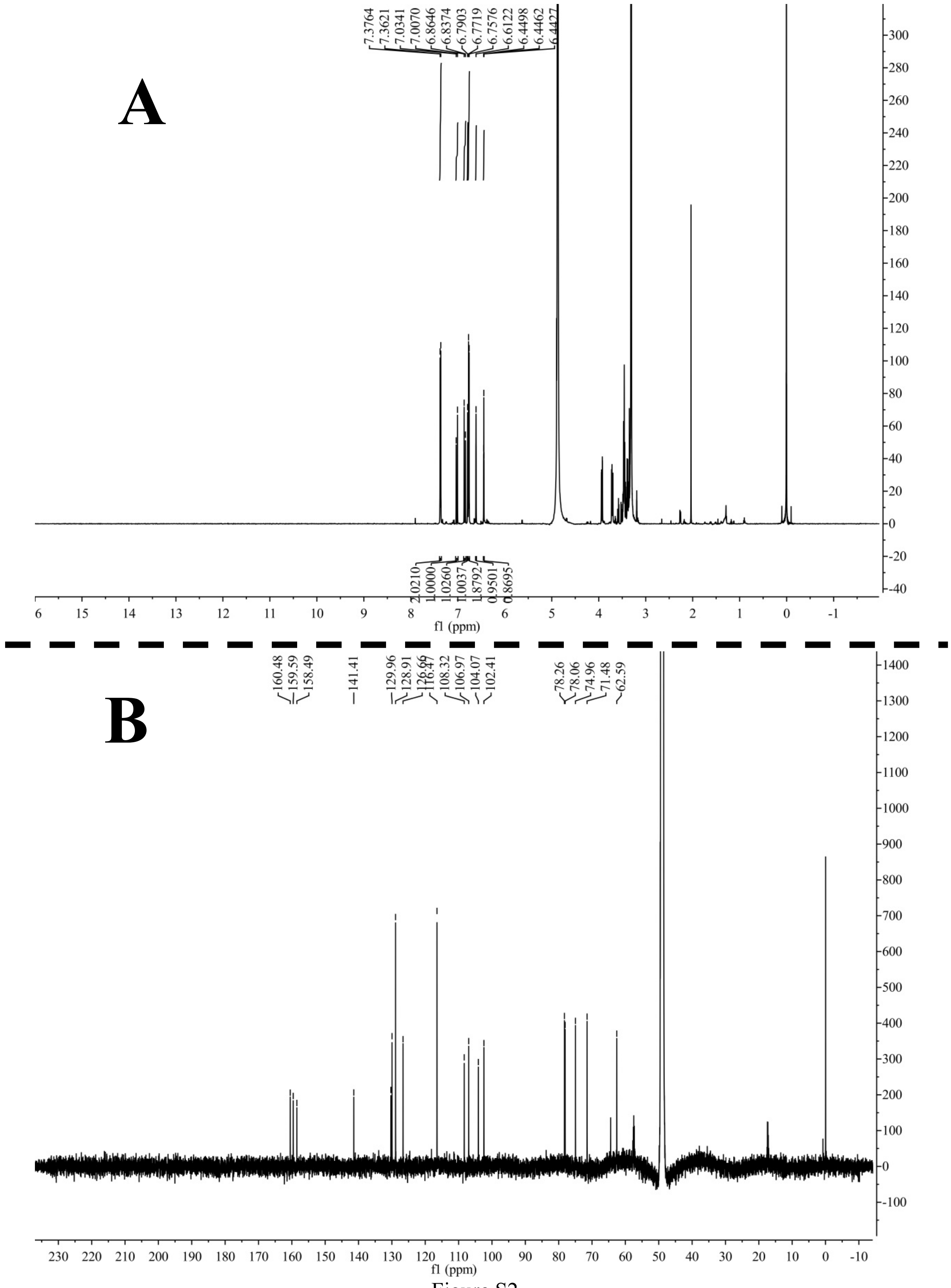
A
7.3764
7.3621
7.0341
7.0070
6.8646
6.8374
6.7903
6.7719
6.7576
6.6122
6.4498
6.4462
6.4427
2.0210
1.0000
1.0260
1.0037
1.8792
0.9501
0.8695
f1 (ppm)
B
160.48
159.59
158.49
141.41
129.96
128.91
126.66
108.32
106.97
104.07
102.41
78.26
78.06
74.96
71.48
62.59
f1 (ppm)

Figure S2

A

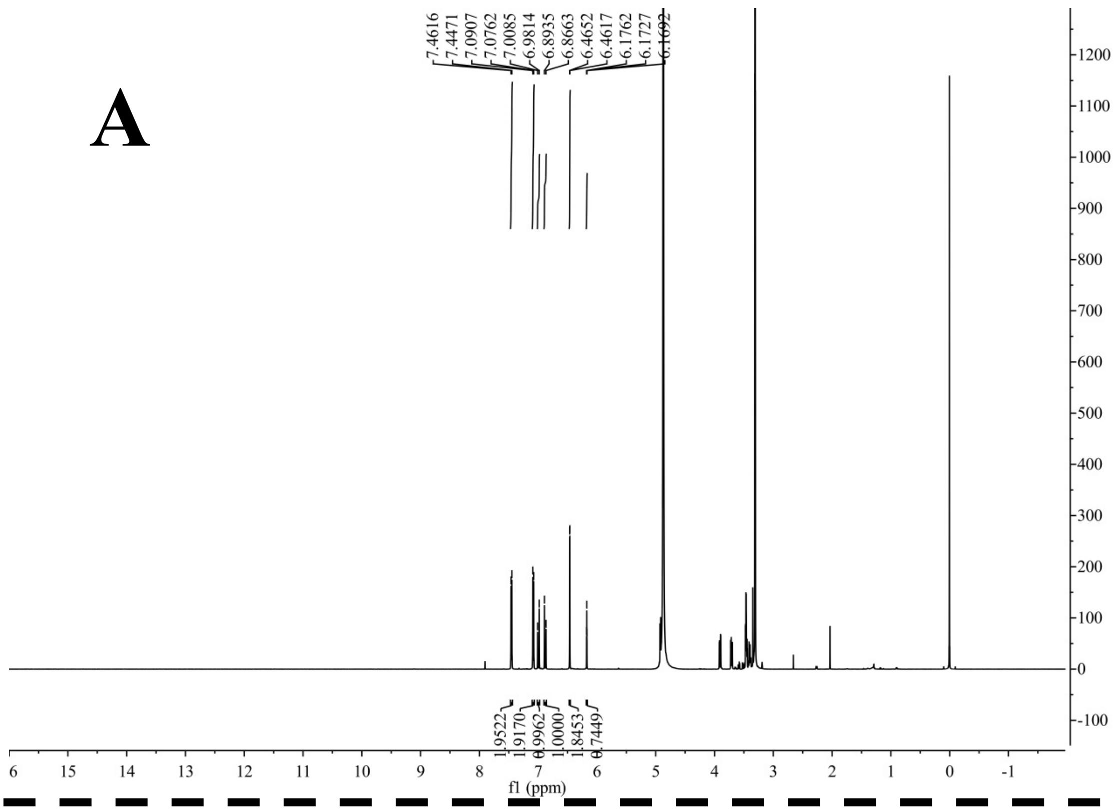

7.4616
7.4471
7.0907
7.0762
7.0085
6.9814
6.8935
6.8663
6.4652
6.4617
6.1762
6.1727
6.1692
1.9522
1.9170
0.9962
1.0000
1.8453
0.7449
f1 (ppm)

B

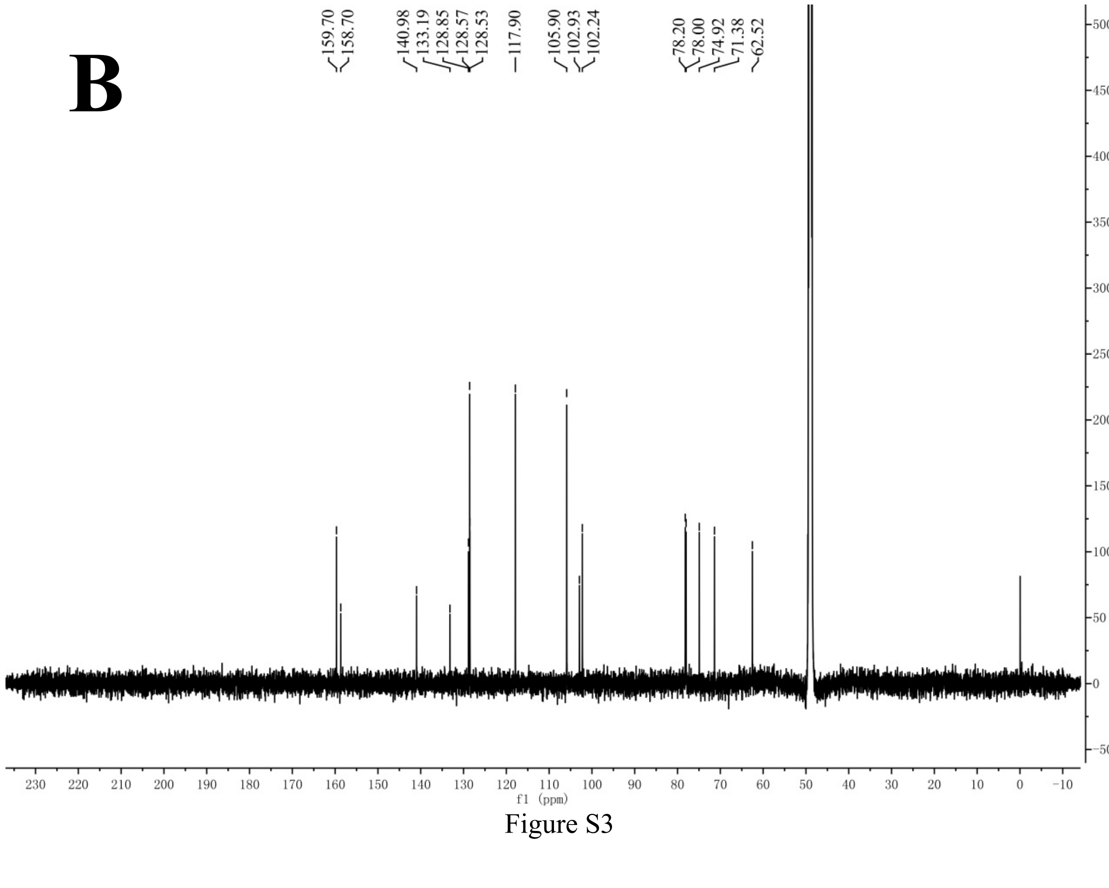

159.70
158.70
140.98
133.19
128.85
128.57
128.53
117.90
105.90
102.93
102.24
78.20
78.00
74.92
71.38
62.52
f1 (ppm)

Figure S3

Supplementary result section X

Section X is a database presenting HPLC chromatogram (Figure S1), UV spectrum (Figure S1), mass spectrum (Figure S1) and NMR spectrum (Figure S2 and Table S1) for compound **12a**.

Table S1 ^{1}H NMR (600 MHz) and ^{13}C NMR (150 MHz) spectroscopic data (δ in ppm) for **12a** in DMSO-d_6

Figure S1 HPLC chromatogram of glycosylated metabolite **12a** of magnolol (**12**) with OcUGT1 (a) or without OcUGT1 (b). The UV absorption spectrum of **12a** is similar to that of **12**. Both are marked in the top panels (**A**). The mass spectrum of **12a** analyzed on ESI-TOF MS was displayed in the bottom panel (**B**)

Figure S2 ^{1}H NMR spectrum (600 MHz, DMSO-d_6) (A) and ^{13}C NMR spectrum of **12a** (150 MHz, DMSO-d_6) (B)

Table S1

**Position**	**δC**	**δH**
1	127.8, C	
2	152.7, C	
3	114.4, CH	7.11,1H, d(8.5Hz)
4	128.1, CH	7.09,1H, dd(8.5Hz,1.9Hz)
5	132.3, C	
6	131.8, CH	7.03,1H, d(2.0Hz)
7	38.7, CH_2	3.22-3.29, 1H, m
8	138.0, CH	5.90-6.00, 1H, m
9	115.5, CH_2	5.00-5.10, 2H, m
1'	125.5, C	
2'	152.2, C	
3'	116.1, CH	6.82, 1H, d(8.2Hz)
4'	128.1, CH	6.95, 1H, dd(8.2Hz, 2.2Hz)
5'	130.1, C	
6'	131.5, CH	7.01, 1H, d(1.8Hz)
7'	38.7, CH_2	3.22-3.29, 1H, m
8'	138.4, CH	5.90-6.00, 1H, m
9'	115.2, CH_2	5.00-5.10, 2H, m
1''	100.4, CH	4.93, 1H, d(7.7Hz)
2''	73.4, CH	
3''	76.4, CH	3.22-3.29, 1H, m
4''		
5''	69.7, CH	
6''	77.1, CH	
	60.8, CH_2	H of sugar 3.00-3.70

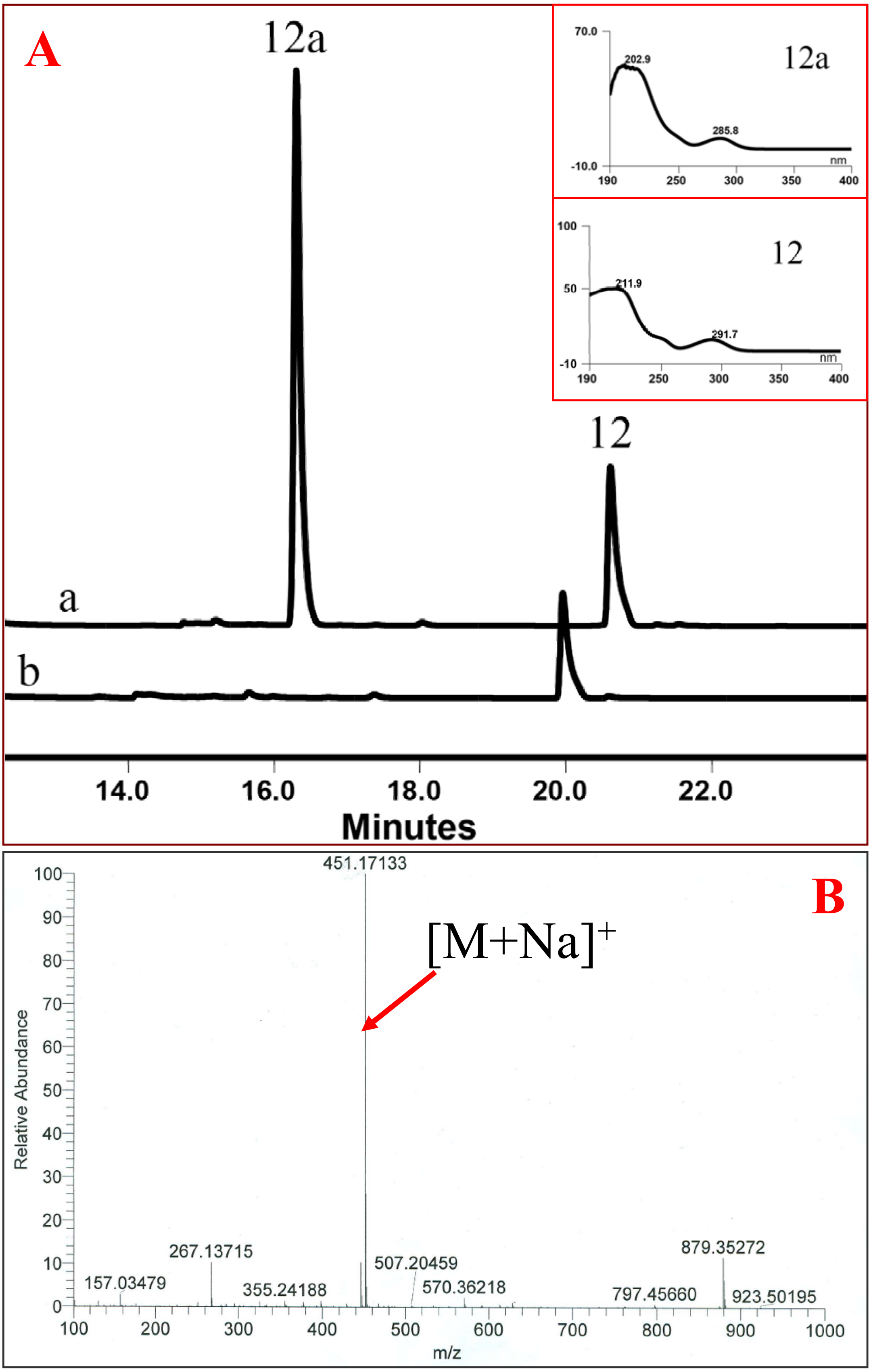
A
12a
70.0
202.9
12a
285.8
-10.0
nm
190
250
300
350
400
100
12
50
211.9
291.7
-10
nm
190
250
300
350
400
12
a
b
14.0
16.0
18.0
20.0
22.0
Minutes
451.17133
B
[M+Na]+
Relative Abundance
100
90
80
70
60
50
40
30
20
10
0
267.13715
157.03479
355.24188
507.20459
570.36218
879.35272
797.45660
923.50195
100
200
300
400
500
600
700
800
900
1000
m/z

Figure S1

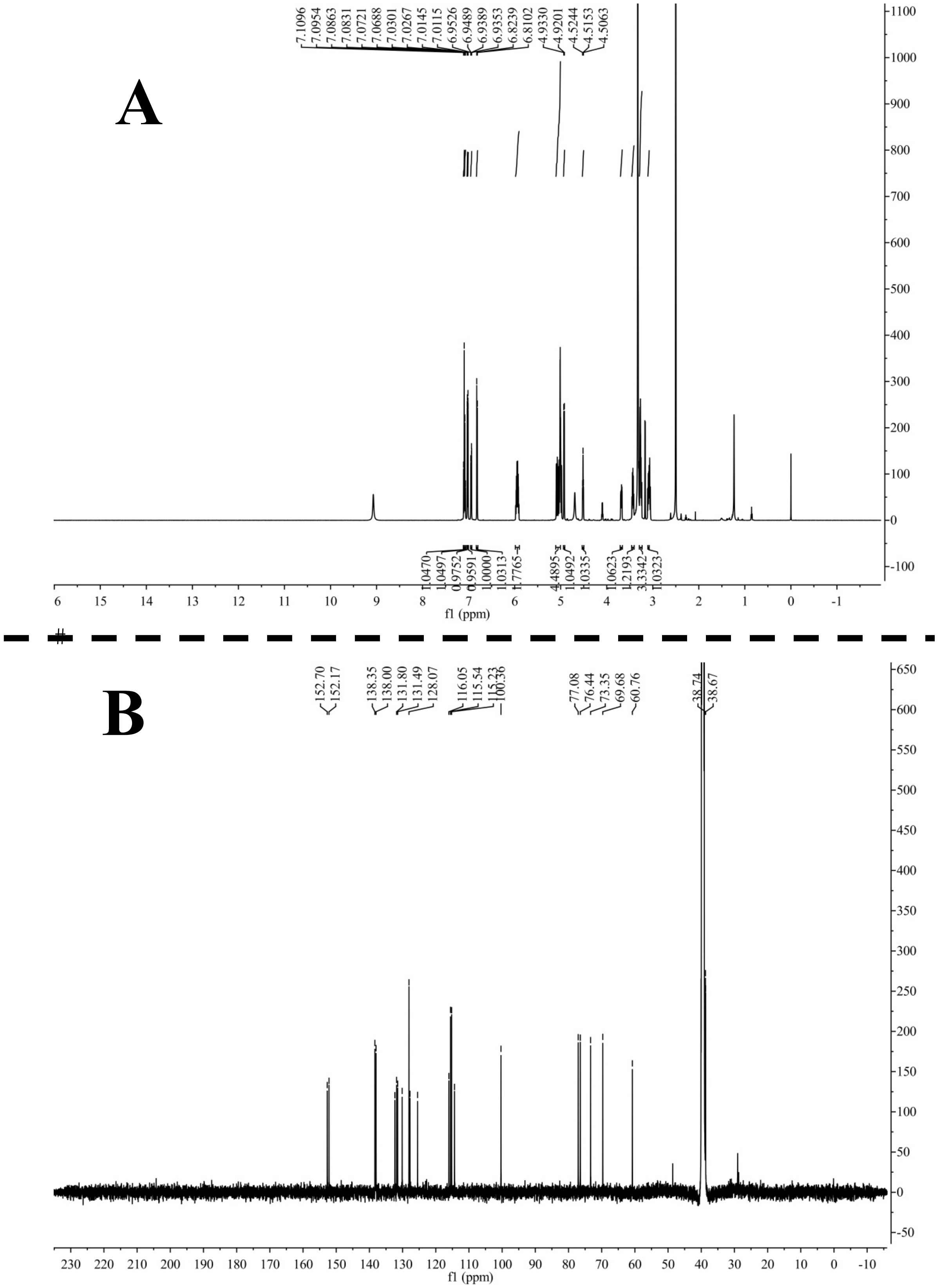
A
7.1096
7.0954
7.0863
7.0831
7.0721
7.0688
7.0301
7.0267
7.0145
7.0115
6.9526
6.9489
6.9389
6.9353
6.8239
6.8102
4.9330
4.9201
4.5244
4.5153
4.5063
1.0470
1.0497
0.9752
0.9591
1.0000
1.0313
1.7765
4.4895
1.0492
1.0335
1.0623
1.2193
3.3342
1.0323
f1 (ppm)
B
152.70
152.17
138.35
138.00
131.80
131.49
128.07
116.05
115.54
115.23
100.36
77.08
76.44
73.35
69.68
60.76
38.74
38.67
f1 (ppm)

Figure S2

Supplementary result section XI

Section XI is a database presenting HPLC chromatogram (Figure S1), UV spectrum (Figure S1), mass spectrum (Figure S1) and NMR spectrum (Figure S2 and Table S1) for compound **13a**.

Table S1 ^{1}H NMR (600 MHz) and ^{13}C NMR (150 MHz) spectroscopic data (δ in ppm) for **13a** in DMSO-d_6

Figure S1 HPLC chromatogram of glycosylated metabolite **13a** of testosterone (**13**) with OcUGT1 (a) or without OcUGT1 (b). The UV absorption spectrum of **13a** is similar to that of **13**. Both are marked in the top panels (**A**). The mass spectrum of **13a** analyzed on ESI-TOF MS was displayed in bottom panel (B).

Figure S2 ^{1}H NMR spectrum (600 MHz, DMSO-d_6) (A) and ^{13}C NMR spectrum of **13a** (150 MHz, DMSO-d_6) (B)

Table S1

Position	*δ*C	*δ*H
1	35.1, CH_2	
2	33.6, CH_2	
3	198.0, C	
4	123.1, CH	5.63, 1H, s
5	171.0, C	
6	31.9, CH_2	
7	31.2, CH_2	
8	34.8, CH	
9	53.3, CH	
10	38.2, C	
11	20.2, CH_2	
12	36.7, CH_2	
13	42.5, C	
14	49.8, CH	
15	22.8, CH_2	
16	28.4, CH_2	
17	86.8, CH	
18	11.3, CH_3	0.80, 3H, s
19	16.9, CH_3	1.15, 3H, s
1'	103.1, CH	4.17, 1H, d(7.8Hz)
2'	73.7, CH	2.91, 1H, m
3'	76.9, CH	3.43, 1H, m
4'	76.9, CH	3.10, 1H, m
5'	70.1, CH	3.43, 1H, m
6'	61.1, CH_2	3.66, 2H, m

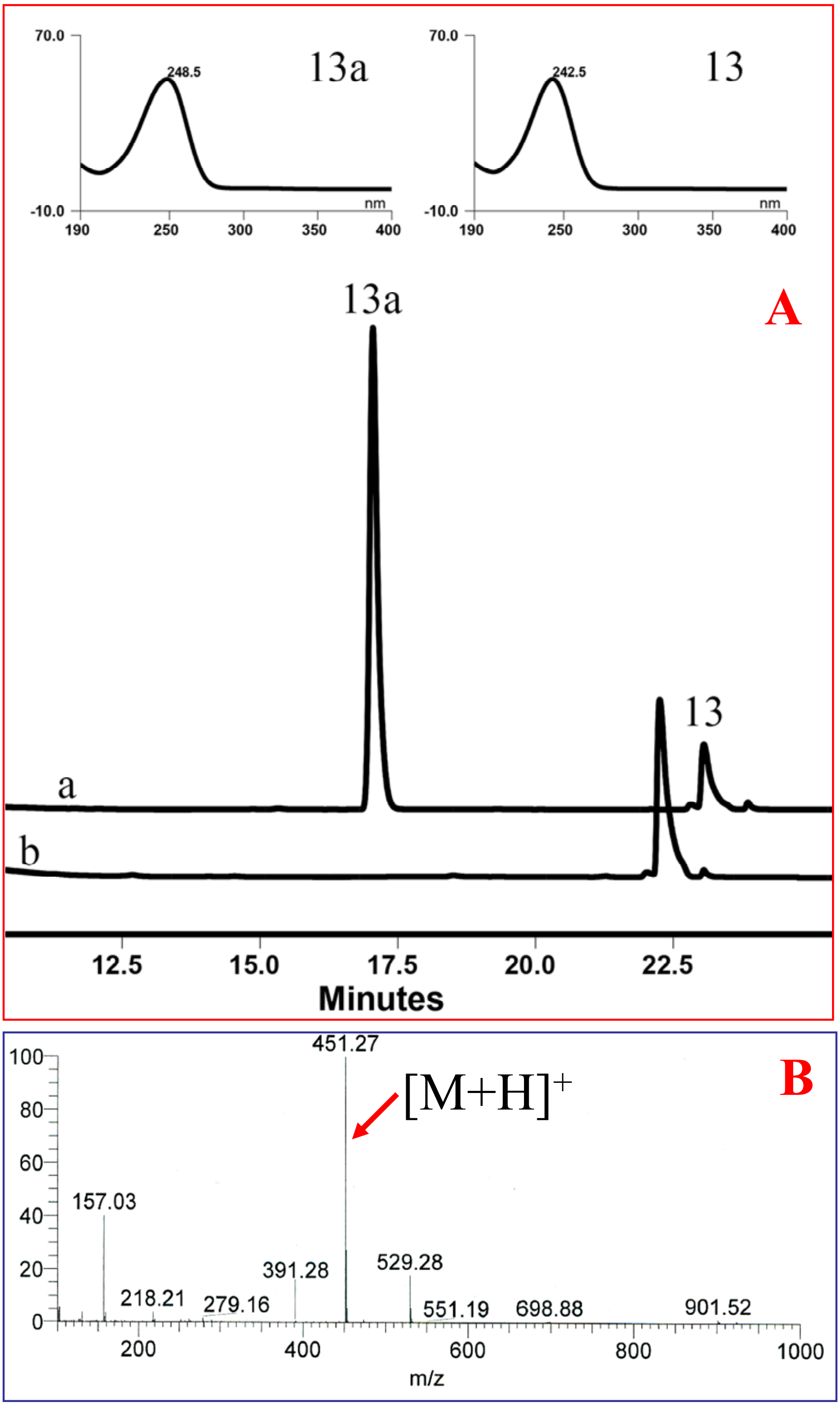

70.0
-10.0
248.5
13a
nm
190
250
300
350
400
70.0
-10.0
242.5
13
nm
190
250
300
350
400
13a
A
13
a
b
12.5
15.0
17.5
20.0
22.5
Minutes
100
80
60
40
20
0
451.27
[M+H]+
B
157.03
218.21
279.16
391.28
529.28
551.19
698.88
901.52
200
400
600
800
1000
m/z

Figure S1

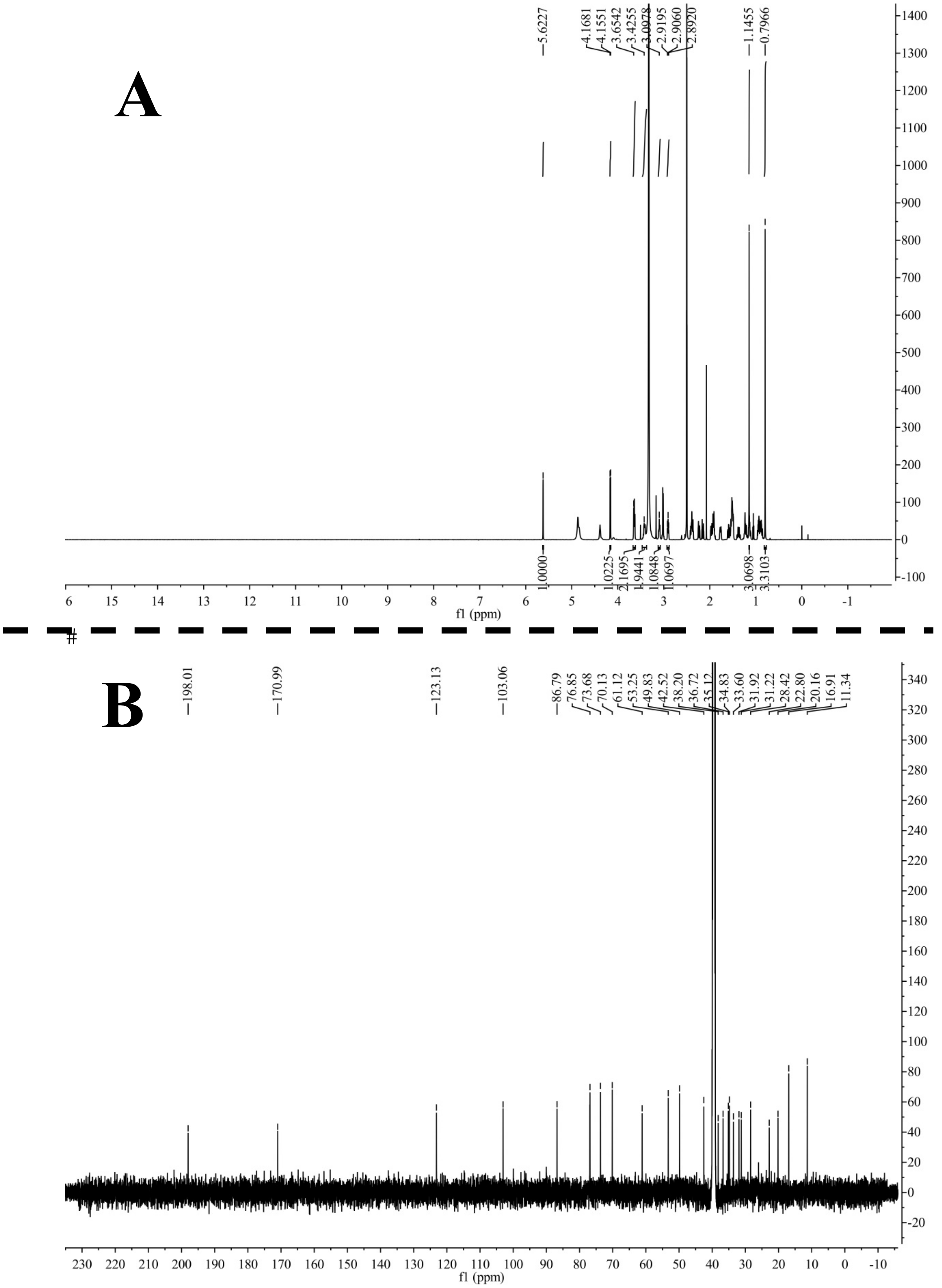
A
5.6227
4.1681
4.1551
3.6542
3.4255
3.0978
2.9195
2.9060
2.8920
1.1455
0.7966
1.0000
1.0225
2.1695
1.9441
1.0848
1.0697
3.0698
3.3103
f1 (ppm)
B
198.01
170.99
123.13
103.06
86.79
76.85
73.68
70.13
61.12
53.25
49.83
42.52
38.20
36.72
35.12
34.83
33.60
31.92
31.22
28.42
22.80
20.16
16.91
11.34
f1 (ppm)

Figure S2

Supplementary result section XII

Section XII presents substrates (Figure S1) and HPLC chromatograms for OcUGT1-catalyzed reactions, including NDP-sugar synthesis (Figures S2-3), sugar exchange (Figure S4), aglycon exchange (Figure S5), glycosylation (Figure S6), hydrolysis (Figures S7-11) and transglycosylation reactions (Figures S12-16).

Figure S1 Substrates used for OcUGT1-catalyzed reactions.

Figure S2 OcUGT1-catalyzed UDP-Glc (**20**) synthesis using *o*NP-β-Glc (**16**) and UDP (**19**) as the substrates.

a, *o*NP-β-Glc (**16**) standard;

b, UDP (**19**) standard;

c, UDP-Glc (**20**) standard;

d, reaction mixture with OcUGT1

e, the control reaction without OcUGT1

The UV absorption spectra of **16, 19** and **20** are marked in the top panels.

Figure S3 OcUGT1-catalyzed UDP-Xyl (**21**) synthesis using *o*NP-β-Xyl (**15**) and UDP (**19**) as the substrates.

a, *o*NP-β-Xyl (**15**) standard;

b, UDP (**19**) standard;

c, UDP-Xyl (**21**) standard;

d, reaction mixture with OcUGT1

e, the control reaction without OcUGT1

The UV absorption spectra of **15, 19** and **21 are** marked in the top panels.

Figure S4 Sugar exchange between *o*NP-β-Xyl (**15**) and UDP-Glc (**20**) catalyzed by OcUGT1 (a) or no OcUGT1 (b). The UV absorption spectra of **15** and **16** are marked in the top panels.

Figure S5 Aglycon exchange between *p*NP-β-Glc (**17**) and 7-hydroxyflavone (**5**) catalyzed by OcUGT1 (a) or no OcUGT1 (b). The UV absorption spectra of **5, 5a, 17** and **17a** are marked in the top panels.

Figure S6 OcUGT1-catalyzed glycosylation of UDP-Glc with **1a** (**a**), **1b** (**b**), **1c** (**c**), **1d** (**d**), **1e** (**e**), **1f** (**f**) and **1g** (**g**).

Figure S7 OcUGT1-assisted hydrolysis toward chrysin-7-glucoside (**2a**)

a, HPLC chromatogram of OcUGT1-catalyzed hydrolysis from chrysin-7-glucoside (**2a**) to chrysin (**2**).

b, HPLC chromatogram of the standard **2a**.

c, HPLC chromatogram of the standard **2**.

UV spectra of **2a** and its hydrolytic product **2** are shown in upper panels.

Figure S8 OcUGT1-assisted hydrolysis toward *o*NP-β-Xyl (**15**)

a, HPLC chromatogram of OcUGT1-catalyzed hydrolysis from *o*NP-β-Xyl (**15**) to *o*NP(**14a**)

b, HPLC chromatogram of the standard 15.

UV spectra of **15** and its hydrolytic product **14a** are shown in upper panels.

Figure S9 OcUGT1-assisted hydrolysis toward *o*NP-β-Glc (**16**)

a, HPLC chromatogram of OcUGT1-catalyzed hydrolysis from *o*NP-β-Glc (**16**) to *o*NP (**14a**).

b, HPLC chromatogram of the standard **16**.

UV spectra of **16** and its hydrolytic product **14a** are shown in upper panels.

Figure S10 OcUGT1-assisted hydrolysis toward *p*NP-β-Glc (**17**)

a, HPLC chromatogram of OcUGT1-catalyzed hydrolysis from *p*NP-β-Glc (**17**) to *p*NP (**17a**)

b, HPLC chromatogram of the standard **17**.

UV spectra of **17** and its hydrolytic product **17a** are shown in upper panels.

Figure S11 OcUGT1-assisted hydrolysis toward *p*NP-α-Glc (**18**)

a, HPLC chromatogram of OcUGT1-catalyzed hydrolysis from *p*NP-α-Glc (**18**) to *p*NP (**17a**)

b, HPLC chromatogram of the standard **18**.

UV spectra of **18** and its hydrolytic product **17a** are shown in upper panels.

Figure S12 OcUGT1-catalyzed intermolecular transglycosylation between *o*NP-β-Glc (**16**) and 6-hydroxyflavone (**3**). UV spectrum of substrates **16** and **3** and their corresponding products **14a** and **3a** are shown in upper panels.

Figure S13 OcUGT1-catalyzed intermolecular transglycosylation between *p*NP-β-Glc(**17)** to 6-hydroxyflavone(**3**). UV spectrum of substrates **17** and **3** and their corresponding products **17a** and **3a** are shown in upper panels.

Figure S14 OcUGT1-catalyzed intermolecular transglycosylation between chrysin-7-glucoside (**2a**) and 6-hydroxyflavone (**3**). UV spectrum of substrates **2a** and **3** and their corresponding products **2** and **3a** are shown in upper panels.

Figure S15 OcUGT1-catalyzed intermolecular transglycosylation between genistein-4'-glucoside (**6a**) and 7-hydroxyflavone (**5**). UV spectrum of substrates **6a** and **5** and their corresponding products **6** and **5a** are shown in upper panels.

Figure S16 OcUGT1-catalyzed transglycosylations between genistein-7-glucoside (**6b**) and 7-hydroxyflavone (**5**). UV spectrum of substrates **6b** and **5** and their corresponding products **6a, 6** and **5a** are shown in upper panels.

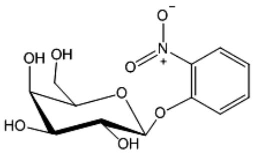

14 oNP-β-Gal

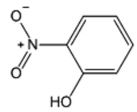

14a *o*-nitrophenol

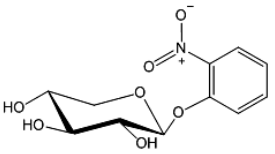

15 oNP-β-Xyl

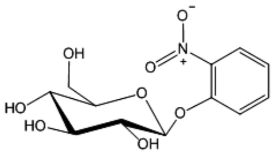

16 oNP-β-Glc

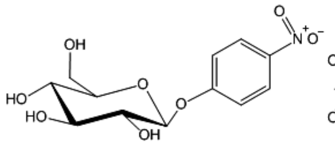

17 pNP-β-Glc

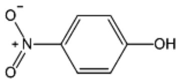

17a *p*-nitrophenol

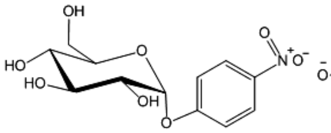

18 pNP-α-Glc

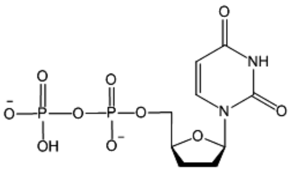

19 UDP

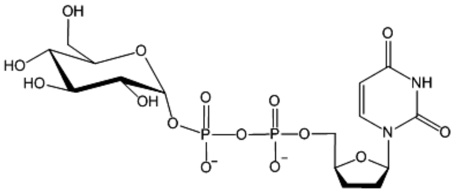

20 UDP-Glc

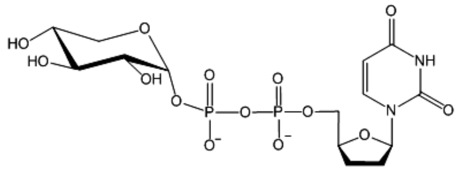

21 UDP-Xyl

Figure S1

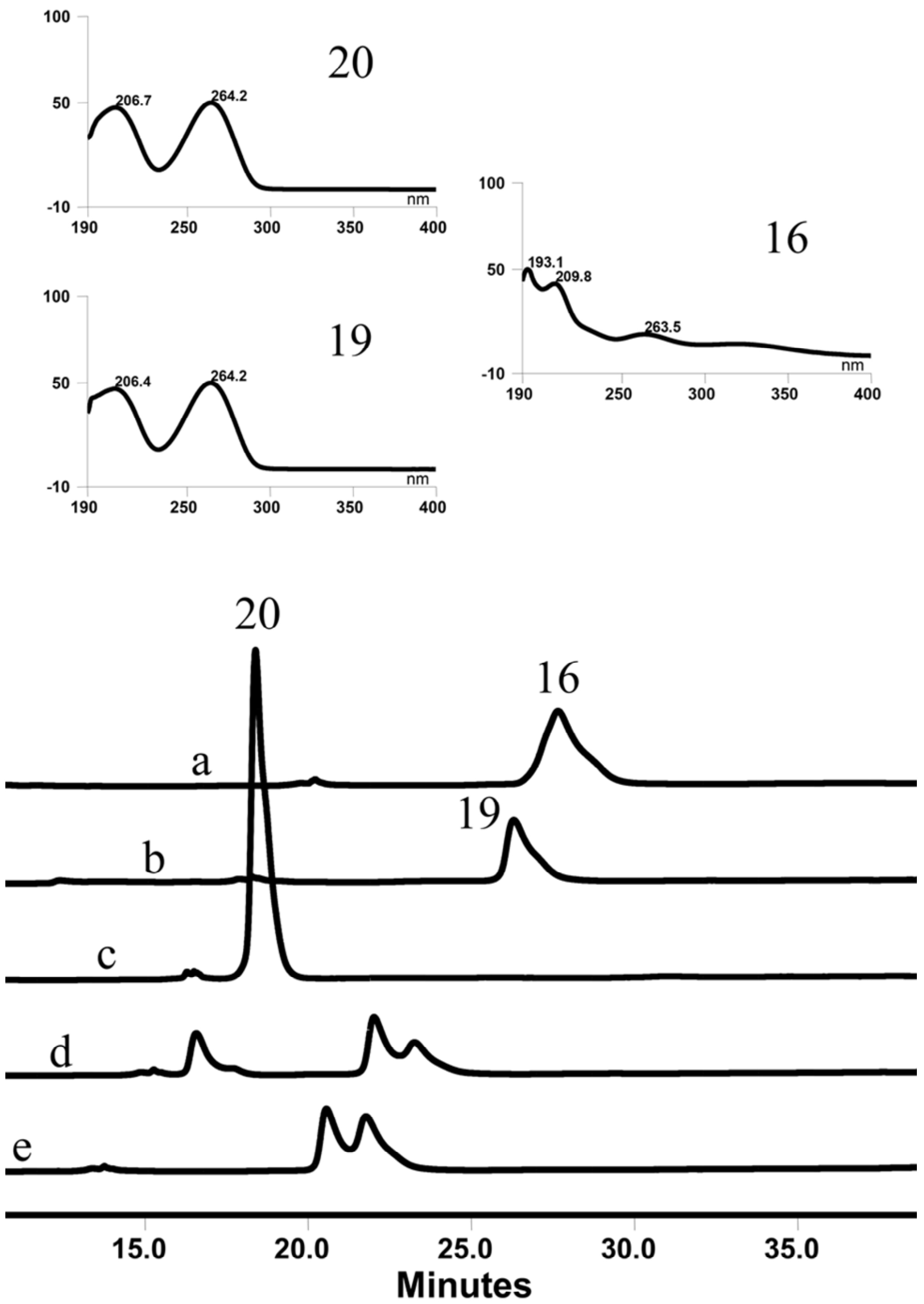

20
206.7
264.2
19
206.4
264.2
16
193.1
209.8
263.5
100
50
-10
nm
190
250
300
350
400
a
b
c
d
e
15.0
20.0
25.0
30.0
35.0
Minutes

Figure S2

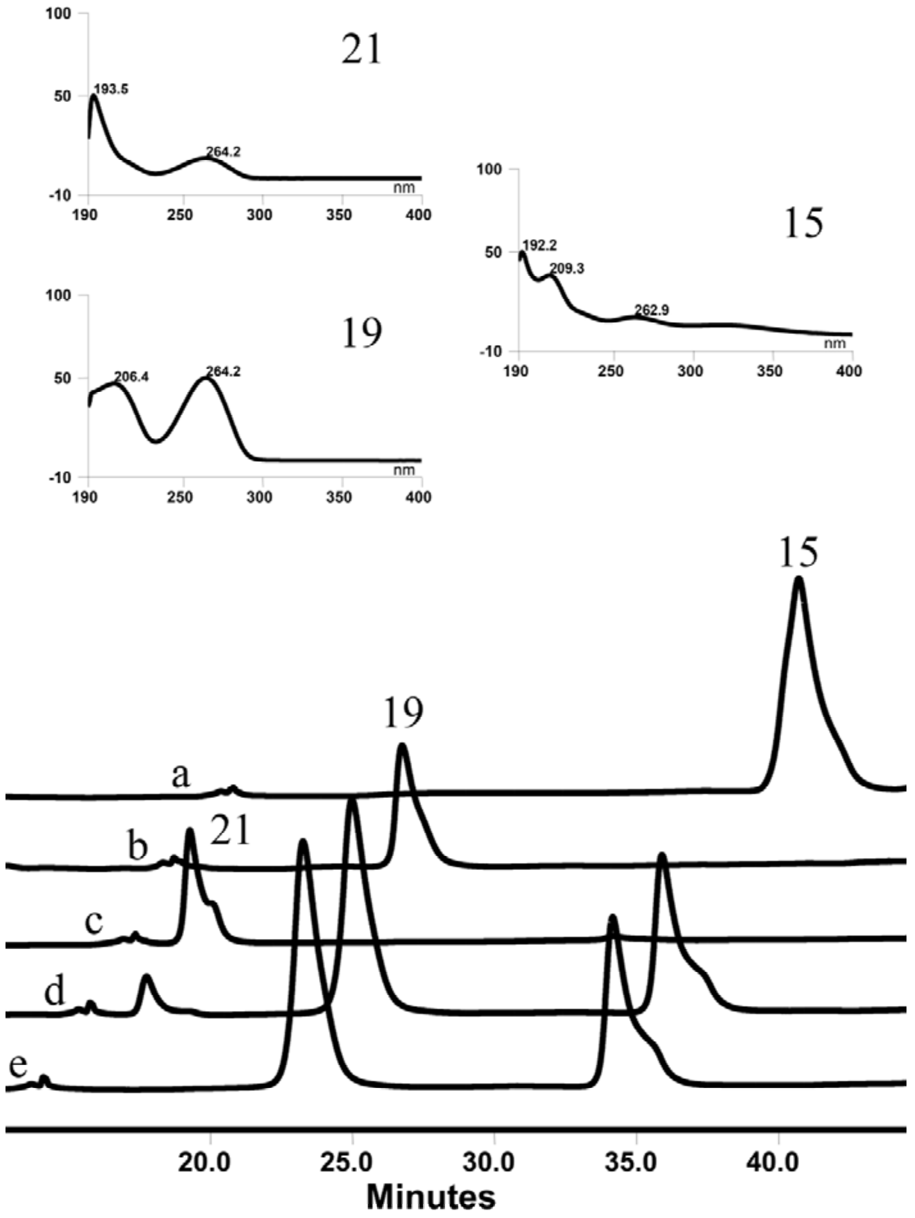

100
50
-10
190
250
300
350
400
nm
21
193.5
264.2
15
192.2
209.3
262.9
19
206.4
264.2
a
b
c
d
e
20.0
25.0
30.0
35.0
40.0
Minutes

Figure S3

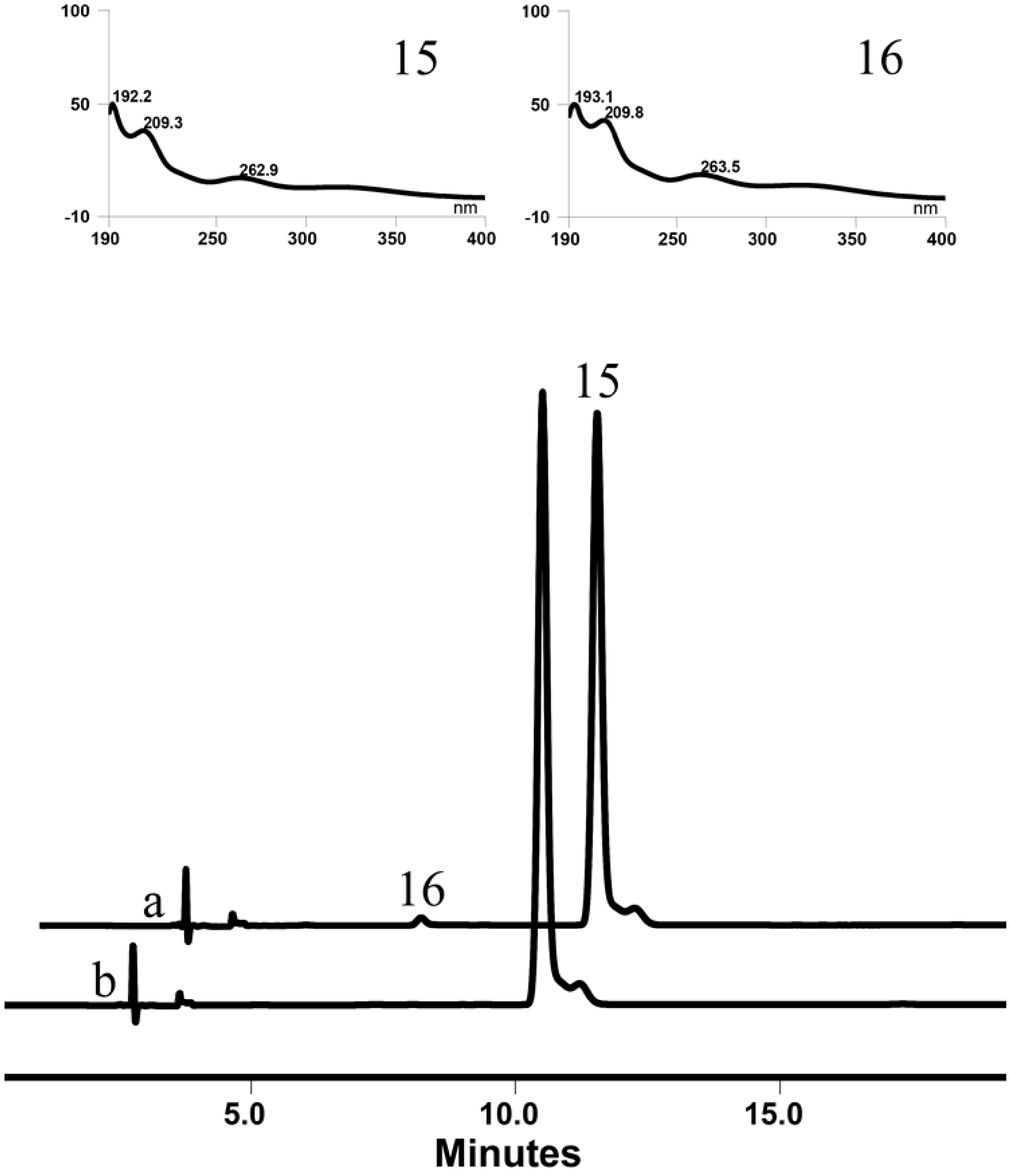
100
15
192.2
50
209.3
262.9
-10
nm
190
250
300
350
400
100
16
193.1
50
209.8
263.5
-10
nm
190
250
300
350
400
15
16
a
b
5.0
10.0
15.0
Minutes

Figure S4

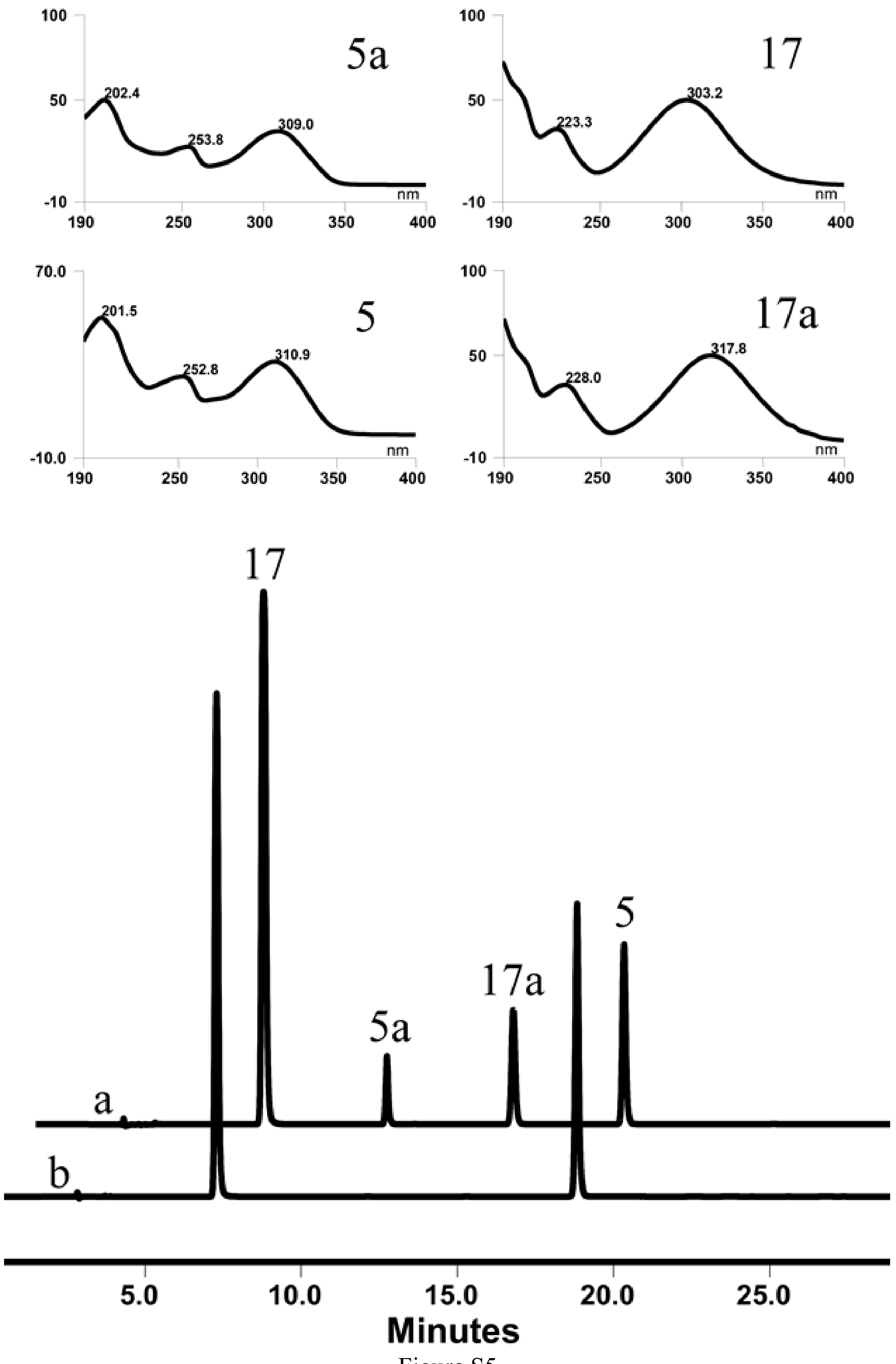

5a
100
50
-10
202.4
253.8
309.0
nm
190 250 300 350 400
17
223.3
303.2
5
70.0
-10.0
201.5
252.8
310.9
17a
228.0
317.8
17
5a
17a
5
a
b
5.0 10.0 15.0 20.0 25.0
Minutes

Figure S5

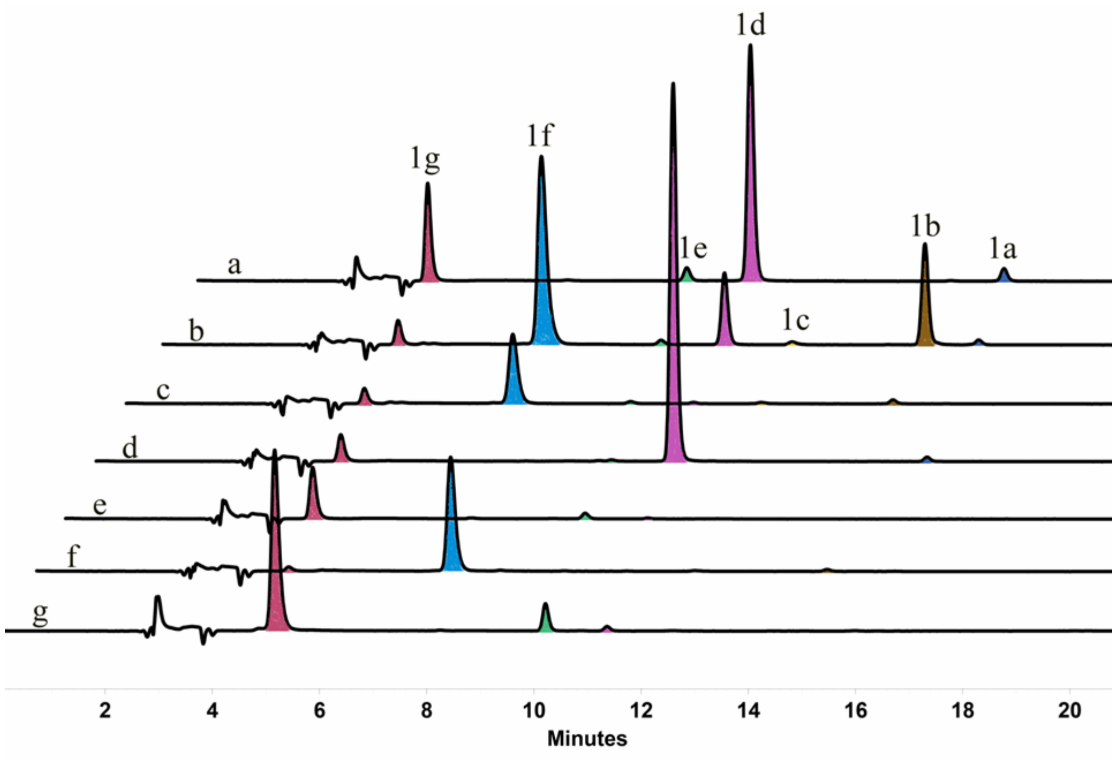

Figure S6

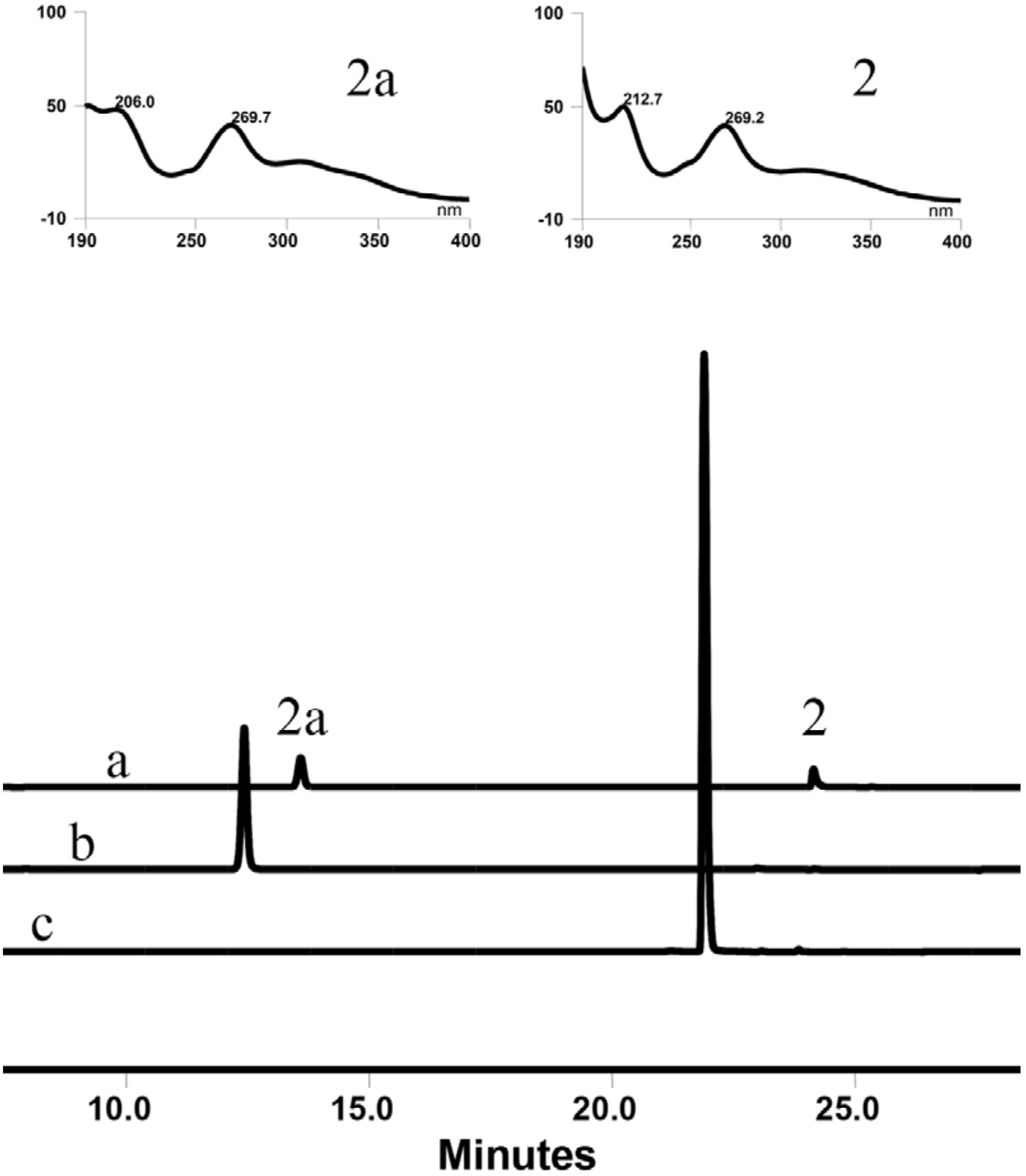

100
50
-10
206.0
269.7
2a
nm
190
250
300
350
400
100
50
-10
212.7
269.2
2
nm
190
250
300
350
400
2a
2
a
b
c
10.0
15.0
20.0
25.0
Minutes

Figure S7

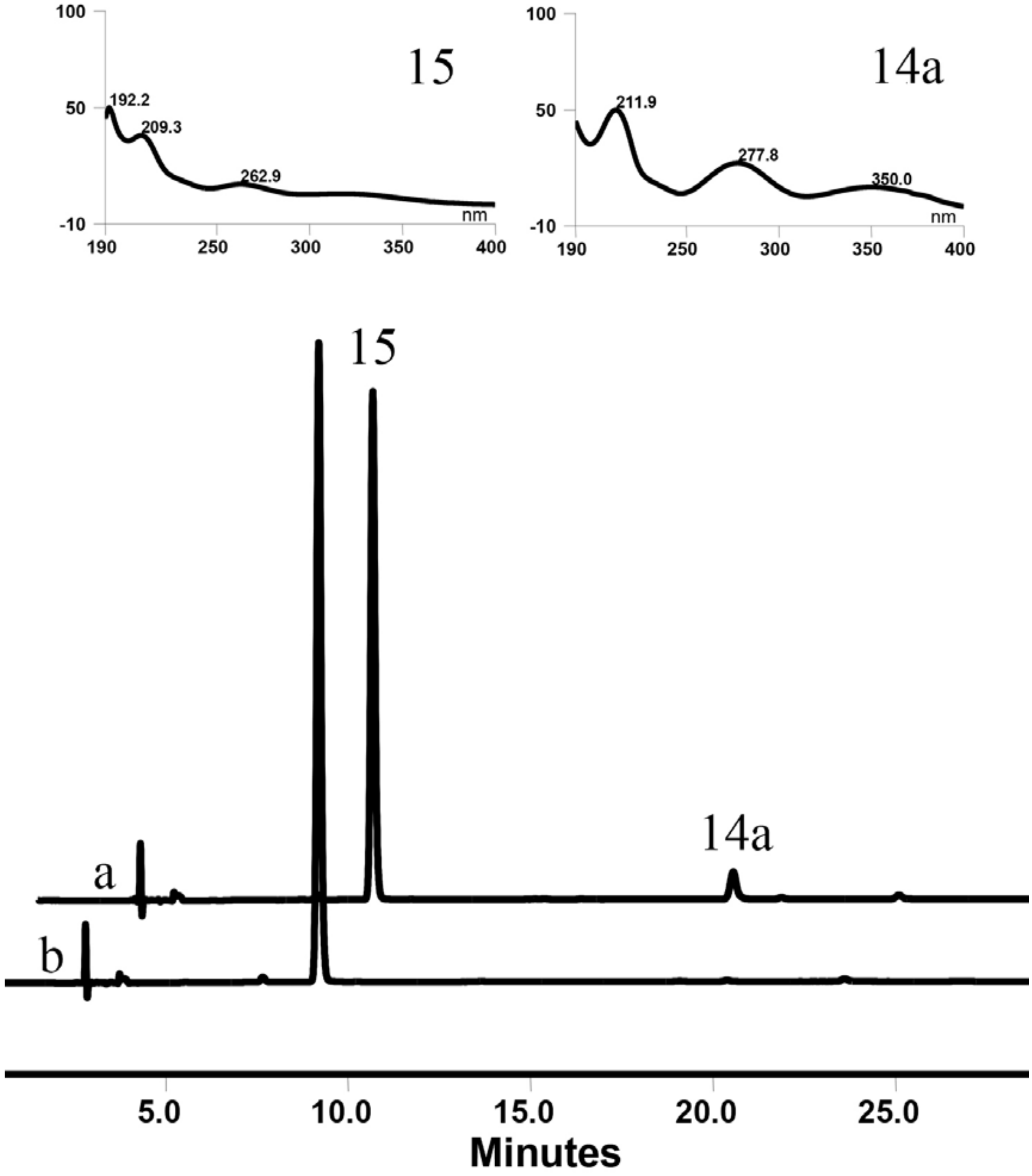
100
50
-10
15
192.2
209.3
262.9
nm
190
250
300
350
400
100
50
-10
14a
211.9
277.8
350.0
nm
190
250
300
350
400
15
14a
a
b
5.0
10.0
15.0
20.0
25.0
Minutes

Figure S8

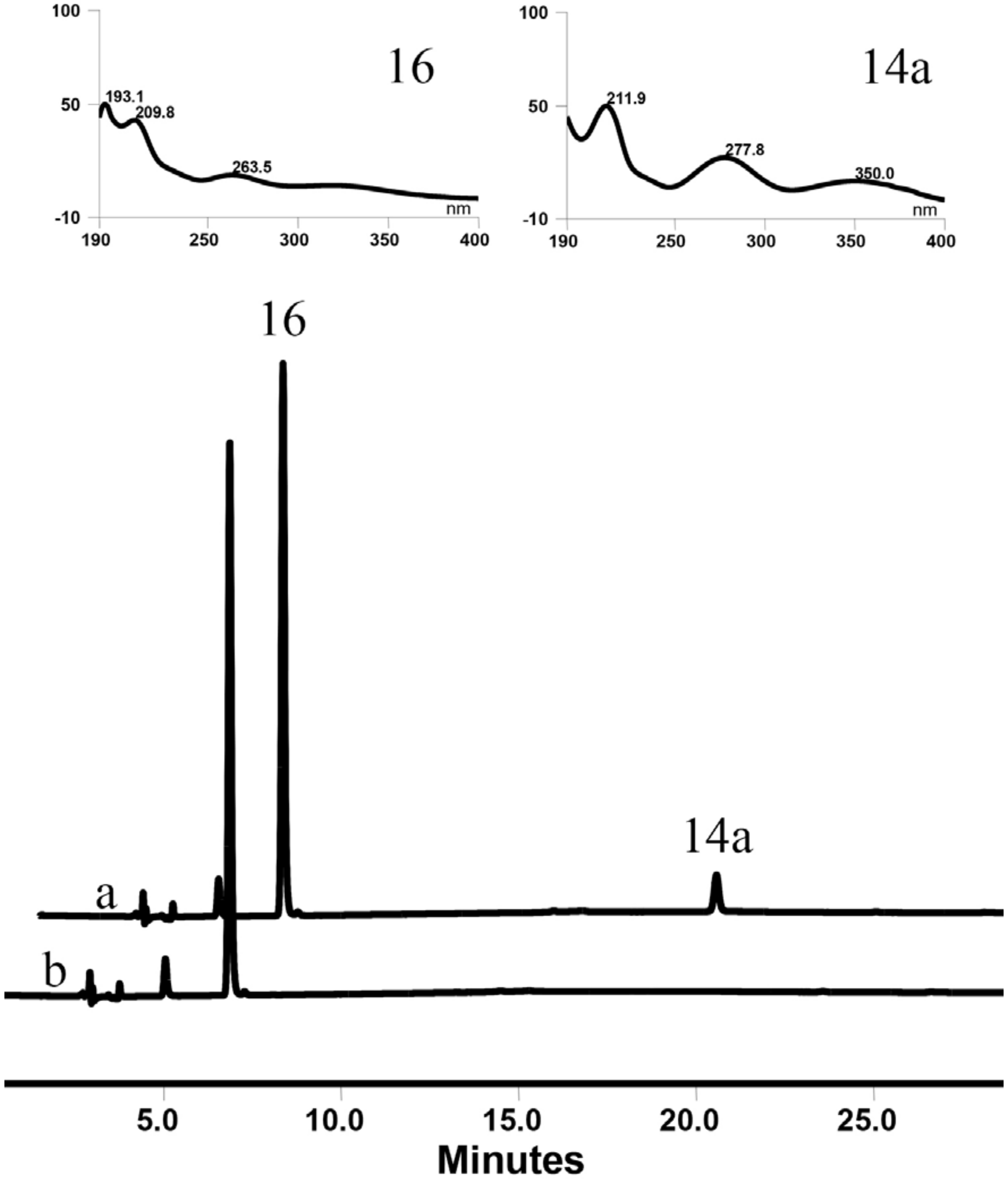

100
50
-10
193.1
209.8
263.5
16
nm
190
250
300
350
400
211.9
277.8
350.0
14a
a
b
5.0
10.0
15.0
20.0
25.0
Minutes

Figure S9

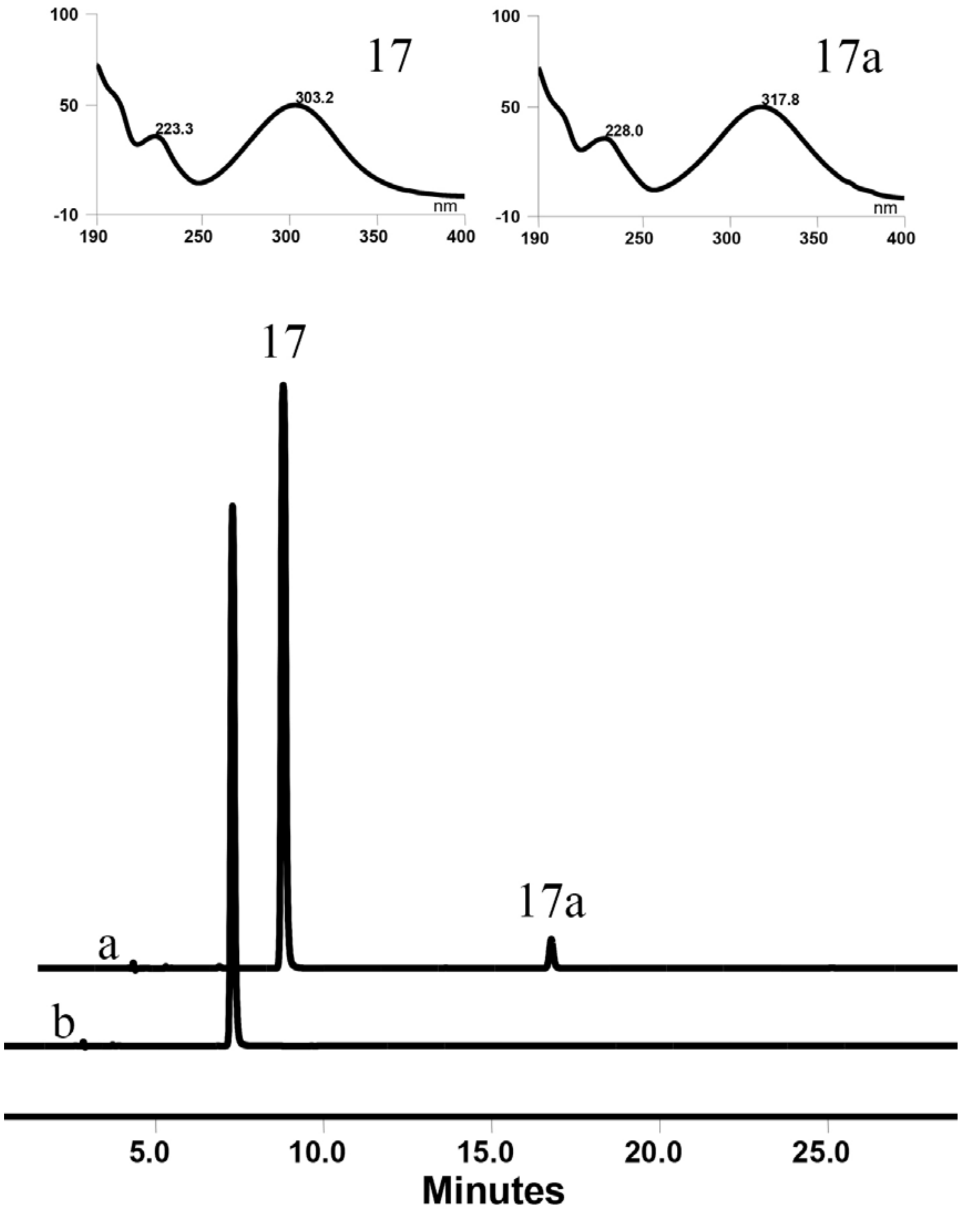

100
50
-10
190
250
300
350
400
nm
17
223.3
303.2
100
50
-10
190
250
300
350
400
nm
17a
228.0
317.8
17
17a
a
b
5.0
10.0
15.0
20.0
25.0
Minutes

Figure S10

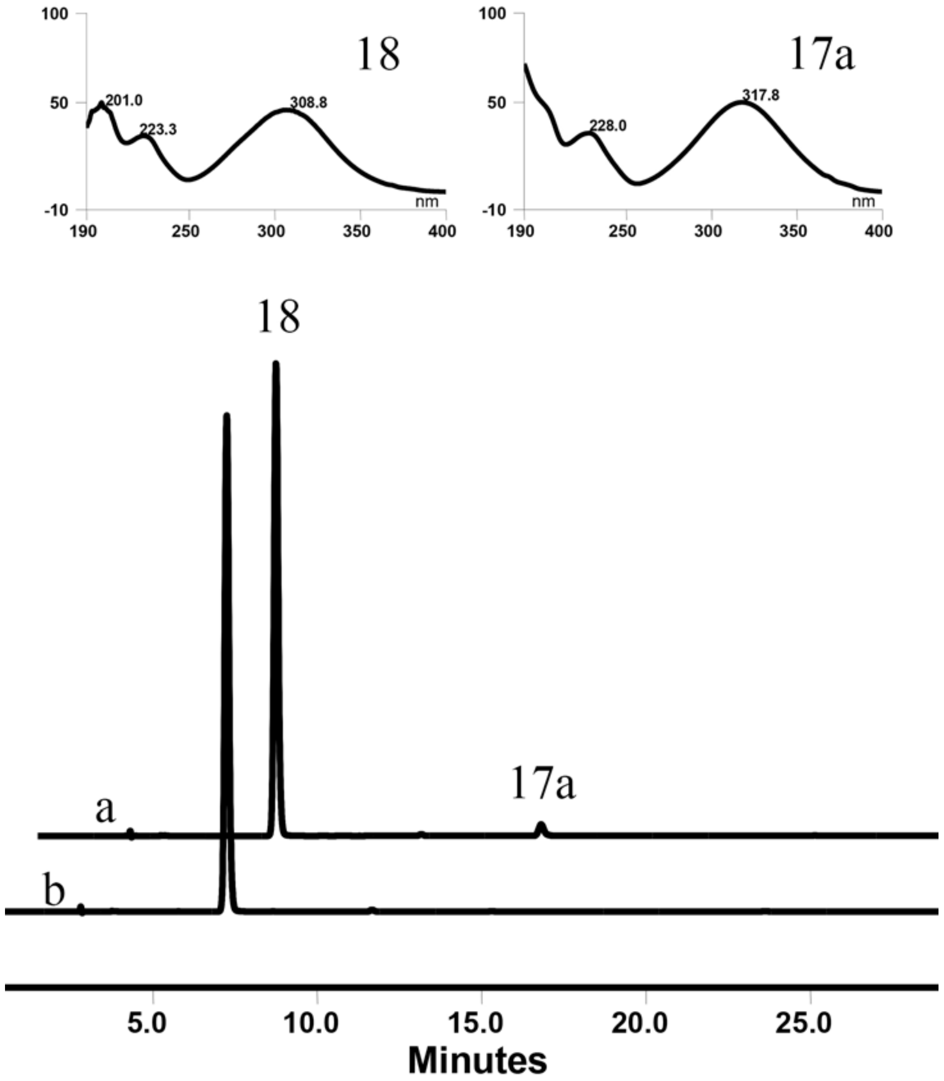
100
50
-10
190
250
300
350
400
nm
18
201.0
223.3
308.8
17a
228.0
317.8
18
17a
a
b
5.0
10.0
15.0
20.0
25.0
Minutes

Figure S11

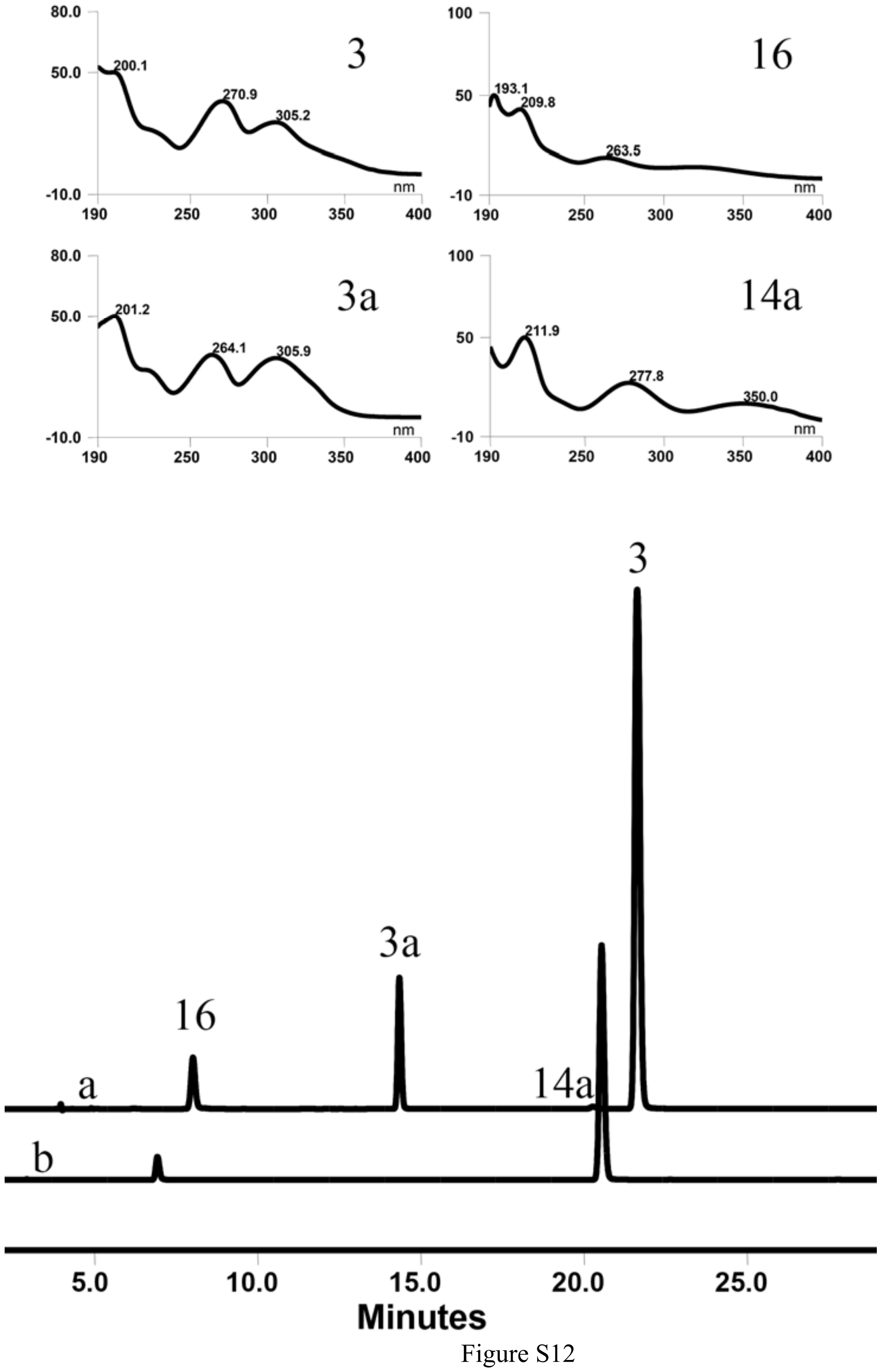

80.0
50.0
-10.0
200.1
270.9
305.2
3
nm
190
250
300
350
400
100
50
-10
193.1
209.8
263.5
16
80.0
50.0
-10.0
201.2
264.1
305.9
3a
100
50
-10
211.9
277.8
350.0
14a
3
3a
16
a
14a
b
5.0
10.0
15.0
20.0
25.0
Minutes

Figure S12

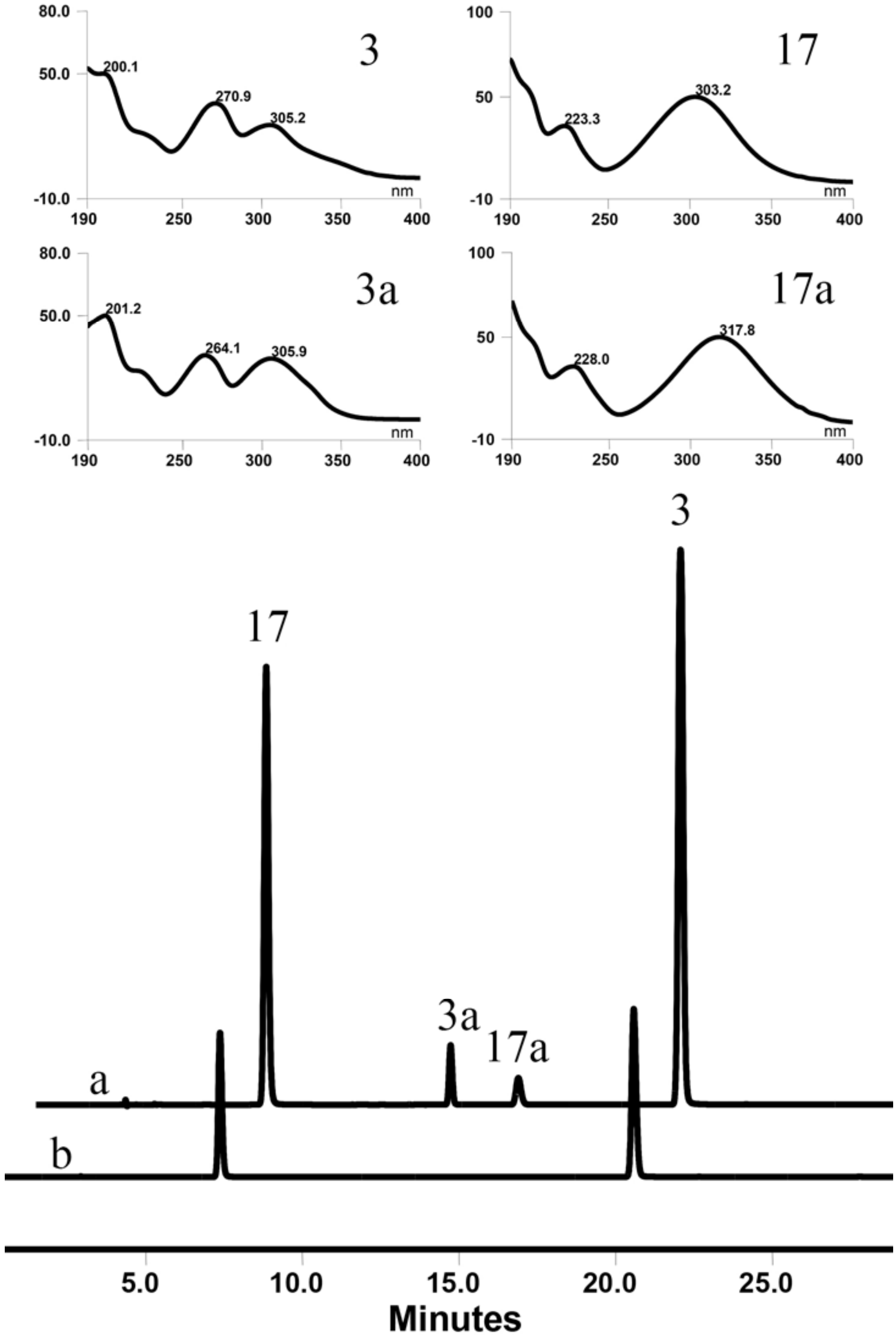

80.0
50.0
-10.0
200.1
270.9
305.2
3
nm
190
250
300
350
400
100
50
-10
223.3
303.2
17
201.2
264.1
305.9
3a
228.0
317.8
17a
3
17
3a
17a
a
b
5.0
10.0
15.0
20.0
25.0
Minutes

Figure S13

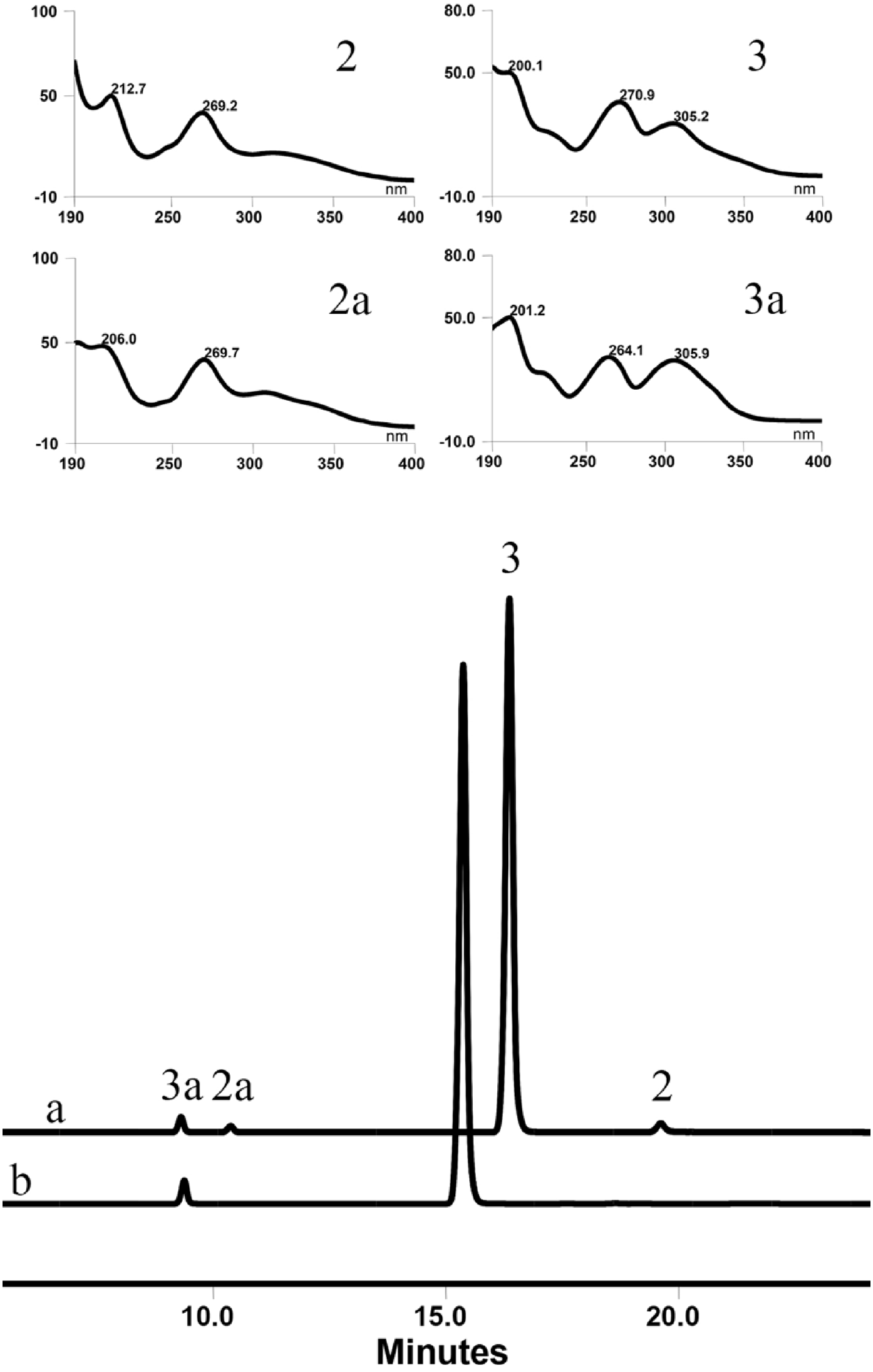

100
50
-10
190
250
300
350
400
nm
2
212.7
269.2
80.0
50.0
-10.0
3
200.1
270.9
305.2
2a
206.0
269.7
3a
201.2
264.1
305.9
3
3a 2a
2
a
b
10.0
15.0
20.0
Minutes

Figure S14

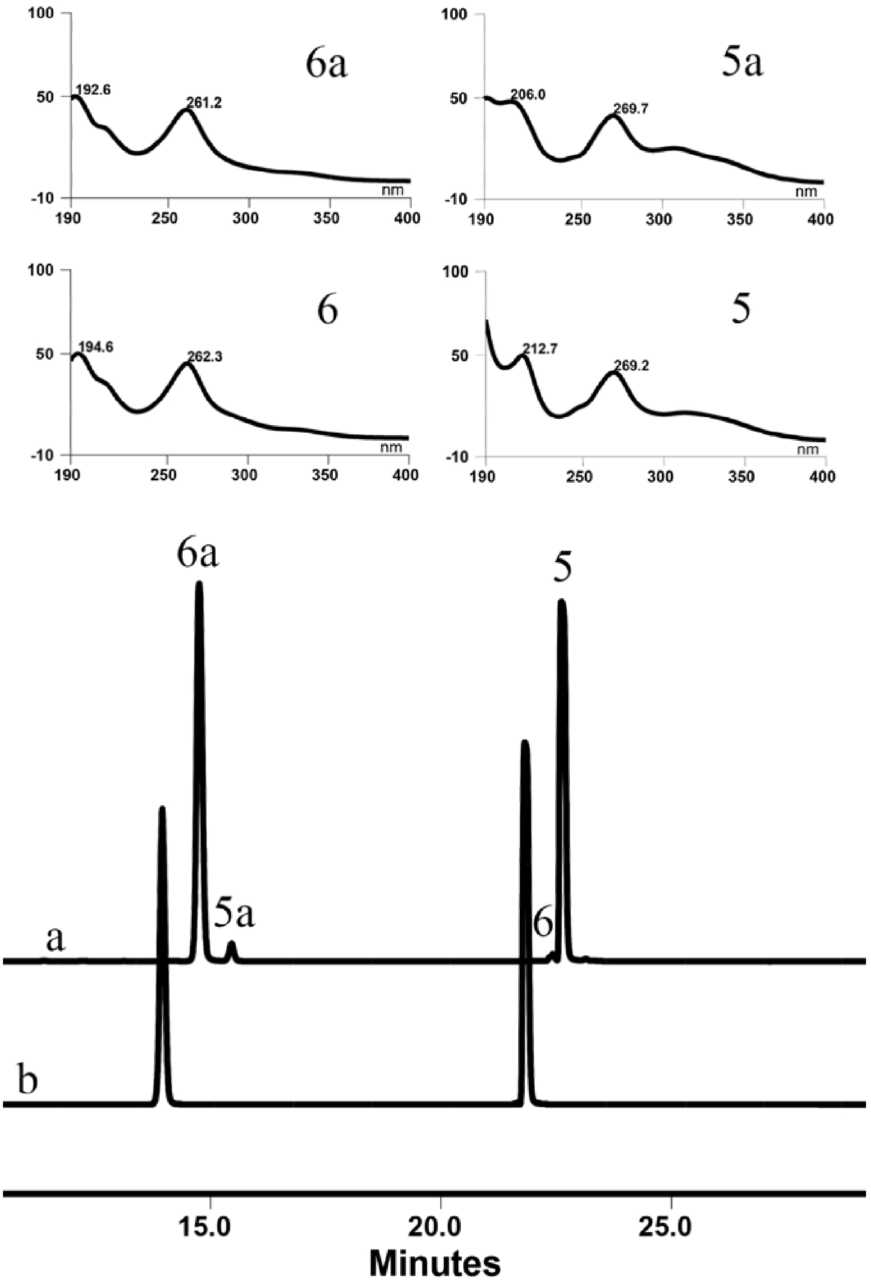

6a
192.6
261.2
5a
206.0
269.7
6
194.6
262.3
5
212.7
269.2
nm
100
50
-10
190
250
300
350
400
6a
5
5a
6
a
b
15.0
20.0
25.0
Minutes

Figure S15

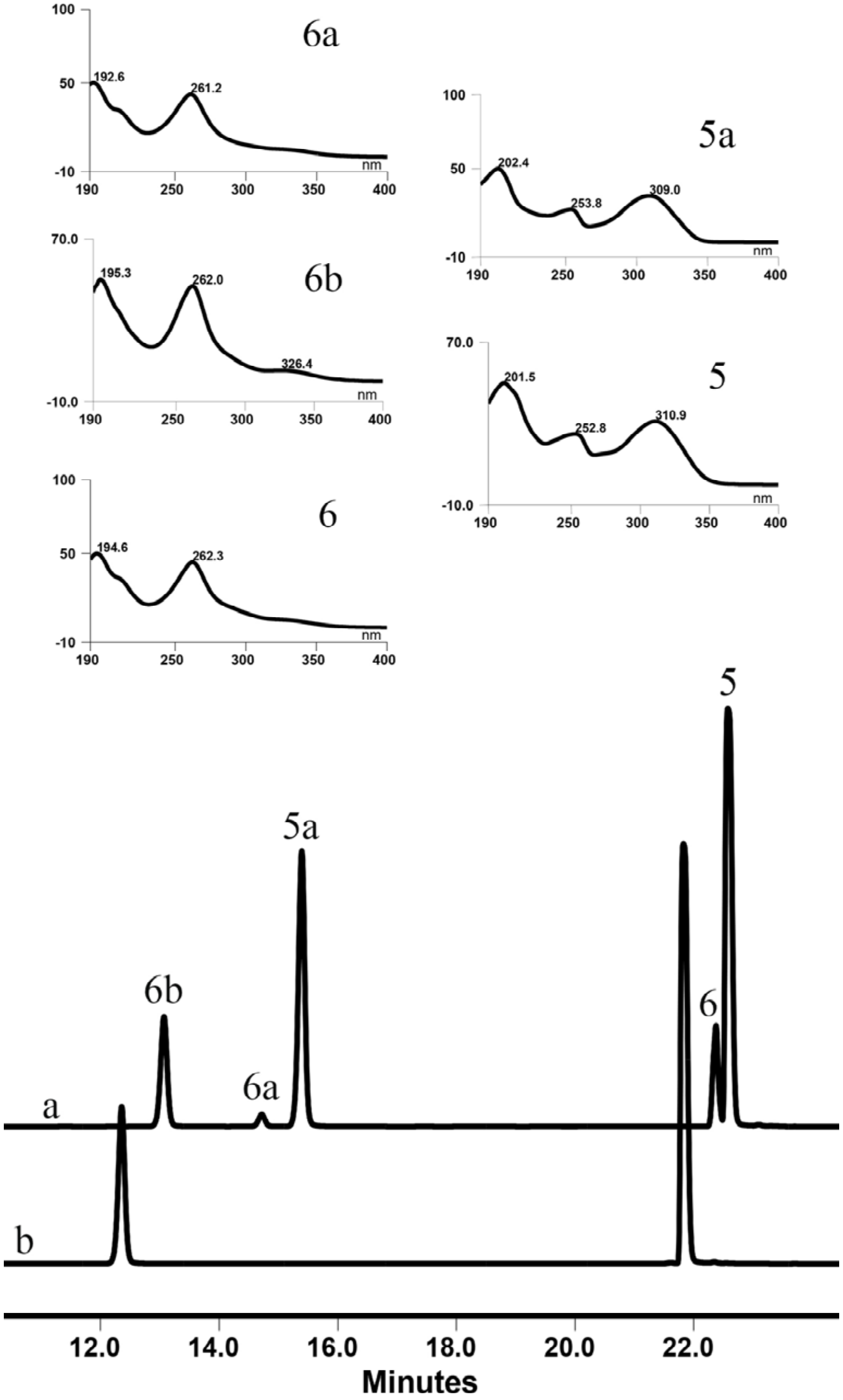

6a
192.6
261.2
5a
202.4
253.8
309.0
6b
195.3
262.0
326.4
5
201.5
252.8
310.9
6
194.6
262.3
nm
5
5a
6b
6
6a
a
b
12.0
14.0
16.0
18.0
20.0
22.0
Minutes

Figure S16

Supplementary materials

Table captions

Table S1 Primers used in this study

Table S2 Plasmids and strains used in this study

Table S3 Chromatographic conditions for HPLC-UV analyses.

Table S1

Primer	Sequence (5′- 3′)	Description
F26797-1	ATATTTCCTATCAGTAATAATT	Forward primer used for OcUGT1 isolation in the first round
R26797-1	ACAAAATTAAACCTTAATTAAG	Reverse primer used for OcUGT1 isolation in the first round
F26797-2	ATGGGCTCCGAGGCCCAT	Forward primer used for OcUGT1 isolation in the second round
R26797-2	CTAAACTGCACTTCTTTTCT	Reverse primer used for OcUGT1 isolation in the second round
FET28a26797	TCGCGGATCCGAATTCATGGGCTCCGAGGCCCAT	Forward primer used for pET28a-OcUGT1 construction
RET28a26797	CCGCAAGCTTGTCGACCTAAACTGCACTTCTTTTCT	Reverse primer used for pET28a-OcUGT1 construction
GT1F	GCATATCGTAGCATCGAGCA	Forward primer used for OcUGT1 qRT-PCR
GT1R	GTGCCCAAACTCTTCAGCTC	Reverse primer used for OcUGT1 qRT-PCR
FGAPDH2	ACTTGGTGTCCACCGACTTC	Forward primer used for GAPDH2 qRT-PCR
RGAPDH2	ATTCGTTGTCGTACCAAGCC	Reverse primer used for GAPDH2 qRT-PCR

Table S2

Strains/plasmids	Description	Source/Reference
Strain		
*Trans*1-T1	F^{-}φ80(*lac*Z)ΔM15Δ*lac*X74*hsd*R(r_k^-,m_k^+)Δ*rec*A1398*end*A1*ton*A	TransGen, Beijing, China
BL21(DE3)	F^{-} *omp*T *hsd*S($r_B^-m_B^-$) *gal dcm*(DE3)	TransGen, Beijing, China
*Trans*etta (DE3)	F^{-}*omp*T*hsd*S_B($r_B^-m_B^-$)*gal dcm* (DE3)pRARE(argU, argW,ilex,glyT,leuW,proL)Cam^r)	TransGen, Beijing, China
*Trans*etta (DE3)[pET28a-OcUGT1]	*Trans*etta (DE3) derived strain containing pET28a-OcUGT1	This study
*Trans*etta (DE3)[pET-28a(+)]	*Trans*etta (DE3) derived strain containing pET-28a(+)	This study
BL21(DE3)[pET28a-OcUGT1+pKJE7]	BL21(DE3) derived strain containing pET28a-OcUGT1 and pKJE7	This study
BL21(DE3)[pET-28^a(+)+pKJE7]	BL21(DE3) derived strain containing pET-28a (+) and pKJE7	This study
Plasmid		
pEASY™-Blunt	General cloning vector, T7 promoter, f1 ori, Amp^r and Kan^r	TransGen, Beijing, China
pET-28a(+)	General expression vector, T7 promoter, f1 ori, Kan^r	Novagen, Madison, USA
pKJE7	Chaperone plasmid, araB promoter, Cm^r	Takara Biotechnology (Dalian) Co., Ltd
pEASY-OcUGT1	*pEASY*™-Blunt derived vector containing *OcUGT1 gene*	This study
pET28a-OcUGT1	pET-28a(+) derived vector containing *OcUGT1 gene*	This study

Table S3

Method	Solvent A	Solvent B	Gradient	Substrates (Detection wavelength)
A	H_2O	Acetonitrile	5% B; 0-5 min<br>30% B; 5.5-10.5 min<br>60% B; 11-16 min<br>90% B; 16.5-21.5 min<br>100%B; 22-27 min<br>5% B; 27.5-30 min<br>Flow rate: 1ml / min	**10**; **10a** (300 nm)<br>**12**; **12a** (290 nm)
B	H_2O	Acetonitrile	15% B-35% B; 0-18 min<br>35% B-100% B; 18-19 min<br>100% B; 19-23 min<br>100% B-15% B; 23-24 min<br>15% B; 24-30 min<br>Flow rate: 1ml / min	**1; 1a; 1b; 1c; 1d; 1e; 1f; 1g; 3; 3a; 7; 7a; 7b; 8; 8a; 11; 11a; 11b** (310 nm)
C	H_2O	Acetonitrile	15% B-70% B; 0-28 min<br>70% B-100% B; 28-29 min<br>100% B; 29-33 min<br>100% B-15% B; 33-34 min<br>15% B; 34-40 min<br>Flow rate: 1ml / min	**14; 14a; 15; 16; 17; 17a; 18** (310 nm)
D	H_2O	Acetonitrile	20% B-40% B; 0-18 min<br>40% B-100% B; 18-19 min<br>100% B; 19-23 min<br>100% B-20% B; 23-24 min<br>20% B; 24-30 min<br>Flow rate: 1ml / min	**5; 5a; 6; 6a; 6b; 9; 9a; 9b** (310 nm)<br>**13; 13a** (250 nm)
E	H_2O	Acetonitrile	25% B-30% B; 0-18 min<br>30% B-100%	**4; 4a; 4b; 4c** (310 nm)

			B; 18-19 min 100% B; 19-23 min 100% B-25% B; 23-24 min 25% B; 24-30 min Flow rate: 1ml / min	
F	H_2O	Acetonitrile	25% B-45% B; 0-18 min 45% B-100% B; 18-19 min 100% B; 19-23 min 100% B-25% B; 23-24 min 25% B; 24-30 min Flow rate: 1ml / min	**2; 2a** (310 nm)
G	8mM Tetrabutylammonium hydrogen sulfate 17mM KH_2PO_4 pH 6.5	70% A 30% Methanol pH 6.5	0% B; 0-5 min 0% B-77% B; 5-32 min 77% B; 32-39 min 77% B-0% B; 39-40 min Flow rate: 1ml / min	**19; 20; 21** (260 nm)

Figure legends

Figure S1 Partial amino acids alignment (A) and phylogenetic tree analysis (B) of OcUGT1 and other known GTs. The "PSPG"motif is highlighted by black box. Following GT sequences were used for constructing phylogenetic tree. Sterol GT [*Withania somnifera*], ABC96116.1; sterol GT [*Dictyostelium discoideum*], AAD28546.1; sterol GT [*Kluyveromyces lactis*], BAG12763.1; sterol GT [*Aspergillus flavus AF70*], KOC14921.1; flavonoid UGT703B1 [*Crocus sativus*], AIF79773.1; flavonoid GT [*Allium cepa*], AAP88406.1; flavonoid GT [*Allium cepa*], AAP88406.1; flavonoid GT [*Actinidia chinensis*], AGV53046.1; anthocyanidin 3-*O*-GT [*Iris* x *hollandica*], BAD83701.1; UGT78D1[*Arabidopsis thaliana*], NM_102790.3; UGT73C6[*Arabidopsis thaliana*], NM_129234.2; anthocyanidin 3-*O*-GT [*Arabidopsis thaliana*], NM_121711.4; UGT73AE1 [*Carthamus tinctorius*], KJ956788.1; coniferyl aldehyde UGT72E1 [*Arabidopsis thaliana*], AEE78703.1; UGT 72E2 [*Arabidopsis thaliana*], NP_201470.1; UGT 72E2 [*Arabidopsis thaliana*], NP_201470.1; sterol GT [*Avena sativa*], CAB06081.1; flavonol 3-*O*-GT [*Petunia* x *hybrida*], AAD55985.1; flavonoid 3-*O*-GT [*Vitis vinifera*], AF000371.1; flavonol 3-*O*-GT [Maize], X13501.1; anthocysnin 5-*O*-GT [*Perilla frutescens*], AB013596.1; anthocysnin 5-*O*-GT [*Glandularia* x *hybrida*], BAA36423.1; flavonoid 7-*O*-GT [*Scutellaria baicalensis*], BAA83484.1; flavonoid 7-*O*-GT [*Scutellaria baicalensis*], AB031274.1; flavonoid GT [*Pyrus communis*], AY954922.1; flavonoid GT [*Malus* x domestica], AY786997.1; isoflavone 7-*O*-GT [*Pueraria Montana*], KC473566.1; isoflavone 7-*O*-GT [*Glycine max*], NM_001248232.1; anthocyanin 5-*O*-GT [*Arabidopsis thaliana*], NM_117485.3; flavonol 5-*O*-GT [*Paeonia lactiflora*], JQ070807.1; anthocyanin 5-*O*-GT [*Petunia* x *hybrid*], AB027455.1; anthocyanin 5-*O*-GT [*Perilla frutescens*], AB013597.1; anthocyanin 5-*O*-GT [*Gentiana triflora*],

AB363839.1; anthocyanin 5-*O*-GT [*Iris hollandica*], AB113664.1; *cyclo*-DOPA 5-*O*-GT [*Amaranthus tricolor*], KP174811.1; flavonoid 3-*O*-GT [*Ipomoea nil*], LC019116.1; anthocyanidin 3-*O*-GT [*Ipomoea batatas*], JN258961.1; flavonol 3-*O*-GT [*Paeonia lactiflora*], JQ070806.1; sterol 3-*O*-GT [*Eleutherococcus senticosus*], KF498591.1; phenol GT [*Homo sapiens*], M84130.1; UGT2B7 [*Homo sapiens*], NM_001074.2; Sgt1 [*Solanum tuberosum*], U82367.2; rhamnosyltransferase [*P.hybrida*], Z25802.1; Betanidin GT [*Dorotheanthus bellidiformis*],Y18871.1

Figure S2 SDS-PAGE analyses of the recombinant OcUGT1

A, SDS-PAGE analysis of crude extract of BL21(DE3)[pET28a-OcUGT1+pKJE7].

1, the crude extract of induced BL21(DE3)[pET28a-OcUGT1+pKJE7];

2, the crude extract of induced BL21(DE3)[pET-28a(+)+pKJE7];

B, SDS-PAGE analysis of the purified OcUGT1 protein.

1, the crude extract of induced BL21 (DE3) [pET28a-OcUGT1+pKJE7];

2, the column effluent,

3 the purified OcUGT1 protein, which is indicated by a red arrow.

M represents protein marker, and the migration of molecular weight markers is indicated along the left margin as kDa.

Figure S3 The effect of temperature (A, D, G), pH (B, E, H) and metal cation (C, F, I) on OcUGT1-catalyzed glycosylation (A, B, C), hydrolysis (D, E, F) and trans-glycosylation reactions (G, H, I).

Figure S4 qRT-PCR analysis of OcUGT1 in tissues of *O. caudatum*

The transcript level of OcUGT1 in leave was defined as 1, and the relative expressions in other tissues were calculated by means of 2-ΔΔCT. Each value is the mean ± SD of three determinations.

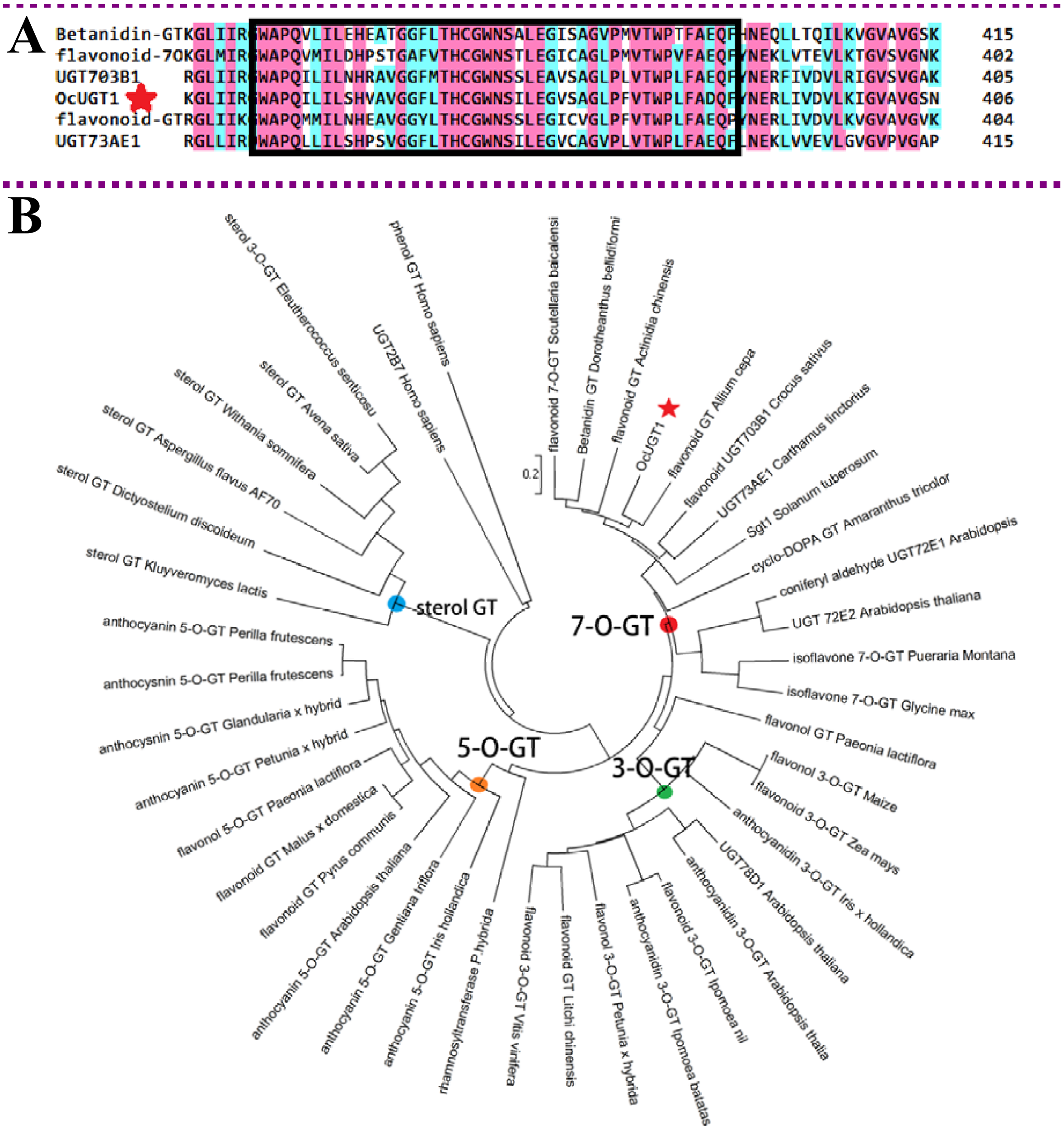
A
Betanidin-GTKGLIIRGWAPQVLILEHEATGGFLTHCGWNSALEGISAGVPMVTWPTFAEQFHNEQLLTQILKVGVAVGSK 415
flavonoid-7OKGLMIRGWAPQVMILDHPSTGAFVTHCGWNSTLEGICAGLPMVTWPVFAEQFVNEKLVTEVLKTGVSVGNK 402
UGT703B1 RGLIIRGWAPQILILNHRAVGGFMTHCGWNSSLEAVSAGLPLVTWPLFAEQFYNERFIVDVLRIGVSVGAK 405
OcUGT1 KGLIIRGWAPQILILSHVAVGGFLTHCGWNSILEGVSAGLPFVTWPLFADQFYNERLIVDVLKIGVAVGSN 406
flavonoid-GTRGLIIKGWAPQMMILNHEAVGGYLTHCGWNSSLEGICVGLPFVTWPLFAEQFYNERLIVDVLKVGVAVGVK 404
UGT73AE1 RGLLIRGWAPQLLILSHPSVGGFLTHCGWNSILEGVCAGVPLVTWPLFAEQFLNEKLVVEVLGVGVPVGAP 415
B
sterol GT
7-O-GT
5-O-GT
3-O-GT
0.2
sterol 3-O-GT Eleutherococcus senticosu
phenol GT Homo sapiens
UGT2B7 Homo sapiens
sterol GT Avena sativa
sterol GT Withania somnifera
sterol GT Aspergillus flavus AF70
sterol GT Dictyostelium discoideum
sterol GT Kluyveromyces lactis
flavonoid 7-O-GT Scutellaria baicalensi
Betanidin GT Dorotheanthus bellidiformi
flavonoid GT Actinidia chinensis
OcUGT1
flavonoid GT Allium cepa
flavonoid UGT703B1 Crocus sativus
UGT73AE1 Carthamus tinctorius
Sgt1 Solanum tuberosum
cyclo-DOPA GT Amaranthus tricolor
coniferyl aldehyde UGT72E1 Arabidopsis
UGT 72E2 Arabidopsis thaliana
isoflavone 7-O-GT Pueraria Montana
isoflavone 7-O-GT Glycine max
flavonol GT Paeonia lactiflora
flavonol 3-O-GT Maize
flavonoid 3-O-GT Zea mays
anthocyanidin 3-O-GT Iris x hollandica
UGT78D1 Arabidopsis thaliana
anthocyanidin 3-O-GT Arabidopsis thalia
flavonoid 3-O-GT Ipomoea nil
anthocyanidin 3-O-GT Ipomoea batatas
flavonol 3-O-GT Petunia x hybrida
flavonoid GT Litchi chinensis
flavonoid 3-O-GT Vitis vinifera
rhamnosyltransferase P.hybrida
anthocyanin 5-O-GT Iris hollandica
anthocyanin 5-O-GT Gentiana triflora
anthocyanin 5-O-GT Arabidopsis thaliana
flavonoid GT Pyrus communis
flavonoid GT Malus x domestica
flavonol 5-O-GT Paeonia lactiflora
anthocyanin 5-O-GT Petunia x hybrid
anthocysnin 5-O-GT Glandularia x hybrid
anthocysnin 5-O-GT Perilla frutescens
anthocyanin 5-O-GT Perilla frutescens

Figure S1

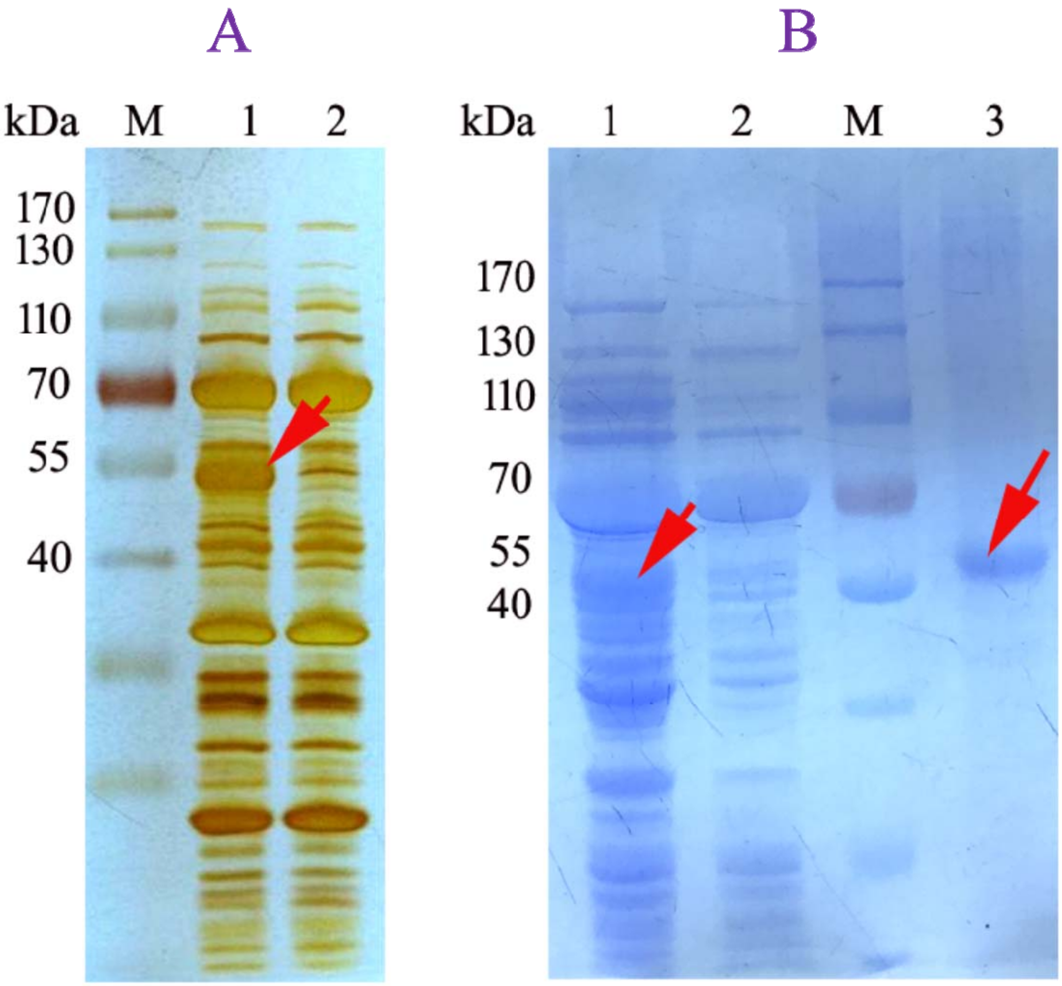

Figure S2

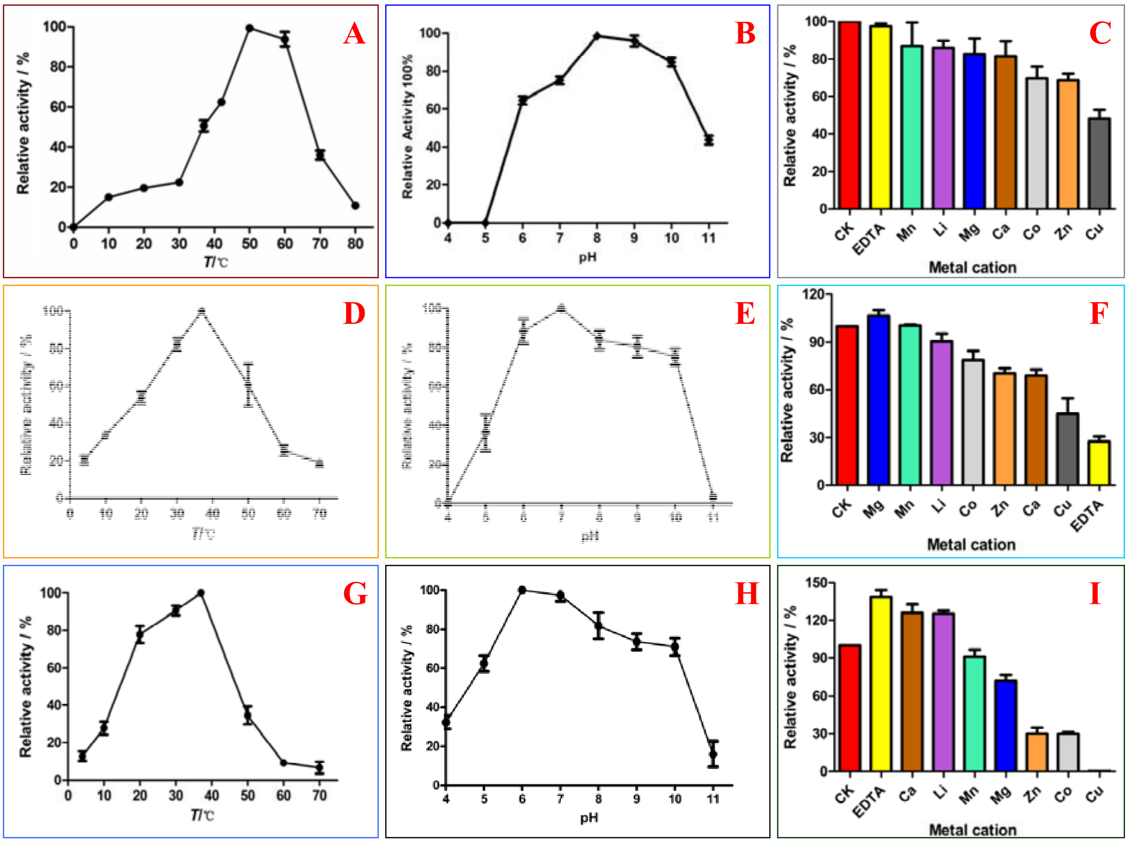
A
B
C
D
E
F
G
H
I
Relative activity / %
Relative Activity 100%
T/℃
pH
Metal cation
CK
EDTA
Mn
Li
Mg
Ca
Co
Zn
Cu

Figure S3

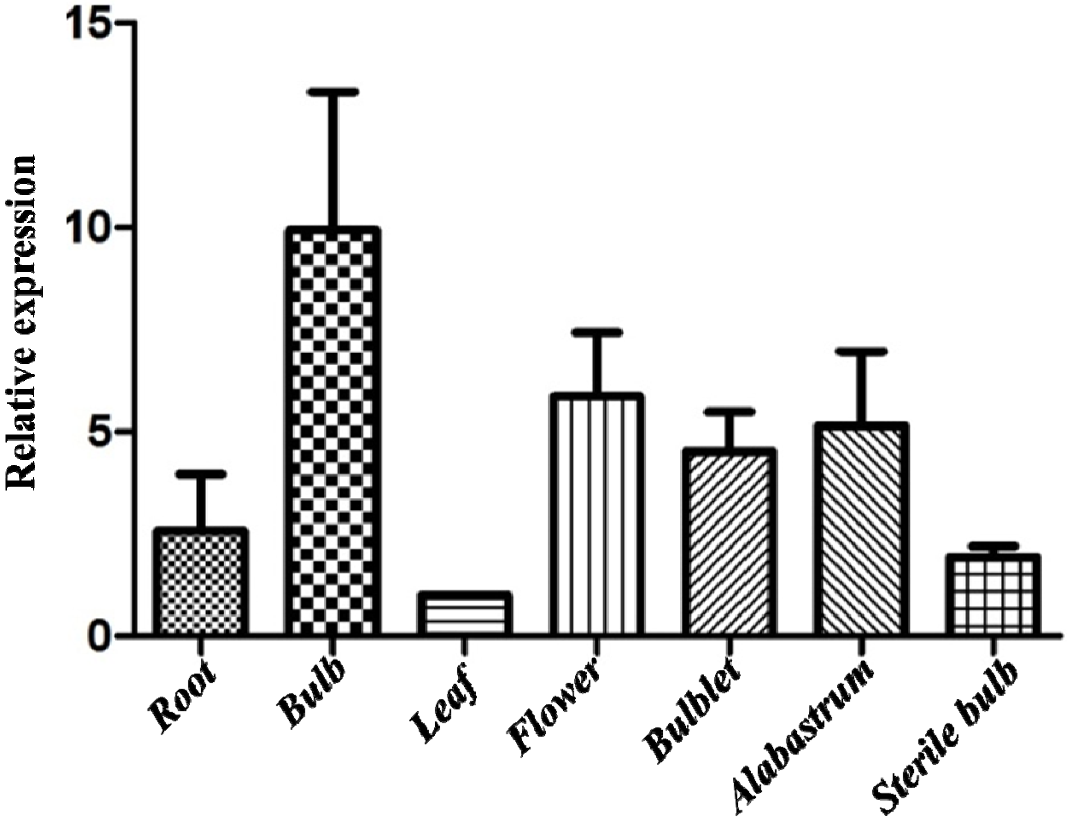
Relative expression
15
10
5
0
Root
Bulb
Leaf
Flower
Bulblet
Alabastrum
Sterile bulb

Figure S4
